# Socio-economic and environmental trade-offs in Amazonian protected areas and Indigenous territories revealed by assessing competing land uses

**DOI:** 10.1038/s41559-024-02458-w

**Published:** 2024-07-15

**Authors:** Bowy den Braber, Johan A. Oldekop, Katie Devenish, Javier Godar, Christoph Nolte, Marina Schmoeller, Karl L. Evans

**Affiliations:** 1 Ecology and Evolutionary Biology, School of Biosciences, University of Sheffield, Sheffield, UK; 2 Department of Geosciences and Natural Resource Management, University of Copenhagen, Copenhagen, Denmark; 3 Global Development Institute, The University of Manchester, Manchester, UK; 4 Stockholm Environmental Institute, Stockholm, Sweden; 5 Department of Earth & Environment, Boston University, Boston, MA USA; 6 Pós-graduação em Ecologia, Universidade Federal do Rio de Janeiro, Rio de Janeiro, Brazil; 7 International Institute for Sustainability Australia, Canberra, Australian Capital Territory Australia

**Keywords:** Sustainability, Environmental economics, Conservation biology

## Abstract

Protected area (PA) assessments rarely evaluate socio-economic and environmental impacts relative to competing land uses, limiting understanding of socio-environmental trade-offs from efforts to protect 30% of the globe by 2030. Here we assess deforestation and poverty outcomes (fiscal income, income inequality, sanitation and literacy) between 2000 and 2010 of strict PAs (SPAs), sustainable-use PAs (SUPAs) and Indigenous territories (ITs) compared with different land uses (agriculture and mining concessions) across ~5,500 census tracts in the Brazilian Legal Amazon. ITs reduced deforestation relative to all alternative land uses (48–83%) but had smaller socio-economic benefits compared with other protection types and land uses (18–36% depending on outcome), indicating that Indigenous communities experience socio-economic trade-offs. By contrast, SUPAs, and potentially SPAs, did not reduce deforestation relative to small-scale agriculture (landholdings <10 ha) but did so relative to larger agricultural landholdings (70–82%). Critically, these reductions in deforestation frequently occurred without negative socio-economic outcomes. By contrast, ITs and SUPAs protected against deforestation from mining, but at the cost of smaller improvements in income and inequality. Our results suggest that although PAs in the Brazilian Legal Amazon substantially reduced deforestation without compromising local socio-economic development, efforts to secure Indigenous rights need additional interventions to ensure these communities are not further disadvantaged.

## Abstract (web summary)

The authors find that protected areas and Indigenous territories in the Brazilian Amazon are highly effective at reducing deforestation driven by agriculture and legal mining concessions, but Indigenous territories typically generate smaller socio-economic benefits than strict and sustainable-use protected areas.

## Main

Protected areas (PAs) are key conservation interventions and are central to new ambitious international targets, such as the global target to protect 30% of the planet by 2030 (30 × 30 agenda)^1^. However, the proposed expansion of PAs has important implications for competition among alternative land uses and rural communities that depend on land and natural resources. Conservation research, policy and practice recognize the importance of socio-economic impacts^2,3^, but analyses that focus on multiple outcomes to assess environmental and socio-economic trade-offs remain scarce^4^. Furthermore, studies that assess outcomes of different protection arrangements relative to a range of specific alternative land uses are extremely limited^5–7^. These gaps hamper the ability of policymakers to understand and balance environmental and socio-economic trade-offs in conservation and development decision-making.

Our primary objective is to contrast environmental and socio-economic outcomes of different protection arrangements with outcomes from alternative land uses, using the Brazilian Legal Amazon (BLA) as a case study. Our study advances previous work in two ways. First, we compare deforestation and socio-economic outcomes (fiscal income, income inequality, literacy and sanitation) between different types of protection to matched non-protected control areas. The protection arrangements are strict PAs (SPAs; equivalent to International Union for Conservation of Nature (IUCN) categories I–IV that restrict natural resource use), sustainable-use PAs (SUPAs; IUCN categories V–VI that allow natural resource use) and Indigenous territories (ITs; which give inalienable land rights to Indigenous communities). Second, we differentiate between non-protected control areas with different land uses to assess how protection outcomes vary when compared with alternative, and often competing, land uses.

In this second comparison, we differentiate non-protected control areas into those that are sparsely populated, dominated by agricultural land uses, and those with legal mining concessions. We further differentiate the agricultural land use category based on the size of the dominant landholding (that is, very small, small, medium or large). This size distinction is important because, globally, large-scale agriculture is responsible for most deforestation^8^. Large-scale agriculture also typically generates trade with distant locations but contributes little to poverty reduction and can exacerbate local poverty by restricting small-scale agriculture^9^. Conversely, the contributions of small-scale agriculture to deforestation are typically lower^10^ and socio-economic benefits tend to be retained locally, as smallholders are often less directly connected to distant agricultural markets^11^. We also compare PA land uses with legal mining concessions. There is considerable concern regarding the impacts of mining on natural habitats, but the effect of mining activities on deforestation and its spatial extent is highly variable^12^. The impacts of mining on local poverty are contested^13^. Mining might improve income, health and education through job creation, while infrastructure development could improve accessibility to schools and hospitals. Mining can, however, also increase inequalities, reduce the economic viability of alternative livelihoods such as agriculture^13^ and adversely affect health^14^.

Several factors justify our focus on the BLA. The region is important biologically and contains the world’s largest continuous tropical forest. However, the BLA is also highly threatened by deforestation and forest degradation. Claims for land and resources are highly contested by different actors, including smallholders, Indigenous peoples and local communities (IPLCs; who historically have been economically and politically marginalized), and economic and political elites who have supported mining and large infrastructure and agro-industrial developments (Supplementary Text 1). Critically, government institutions in Brazil have facilitated the integration of environmental and socio-economic data across sources and spatial scales by making them publicly available.

Deforestation in the Amazon initially peaked in the early 2000s owing to the expansion of soy and cattle production, influenced by technological advancements and favourable market conditions^15^. Deforestation rates dropped after 2004 when the Brazilian government launched the Action Plan for the Prevention and Control of Deforestation in the Legal Amazon that included the expansion of PAs and other interventions^16^. Other initiatives have been linked to increasing deforestation, including the implementation of agrarian settlement schemes aimed at smallholder farms^17^, but large landholders remain responsible for the majority of deforestation^10^. The second largest cause of recent deforestation is mining^18^. Although deforestation has increased again since 2012, rates are half those of the 2004 peak^19^. Competition for land between conservation and alternative land uses has resulted in PA downgrading, downsizing and degazettement events in the Amazon^20^ and the increase of mining operations within PA boundaries^21^.

Effects of PAs vary according to the PA type and local and regional contexts^22,23^. PAs can reduce deforestation by restricting alternative land uses, including agriculture, logging and mining, but this can negatively affect rural livelihoods by limiting access to land and natural resources^3^. Conversely, the management regimes of PAs can support livelihoods and alleviate poverty through numerous mechanisms^22^. These include the following: (1) alternative income-generating opportunities from tourism (for example, ref. ^24^); (2) bolstering the flow of environmental services to areas adjacent to PAs, improving agricultural yields and, by extension, incomes (for example, ref. ^25^); (3) allowing communities to leverage support from governments and non-governmental organizations for development projects (for example, education and amenities)^26^; (4) offering payment for ecosystem services (for example, the Bolsa Floresta programme supporting SUPAs in the state of Amazonas)^27^; and (5) providing local communities with access and management rights to facilitate sustainable use of land and natural resources while restricting more intensive exploitation of these resources^3,28^.

Income opportunities from tourism in the BLA are generally low^29^, and SPAs limit local communities’ access and management rights to natural resources. This is in contrast to SUPAs and ITs that actively promote such rights. While communities in ITs are often autarkic and not integrated into market economies, some households and communities actively engage in commercial agriculture and the sale of forest products^30^. Evidence from the BLA suggests that local communities that can legally access forest resources within SUPAs can successfully catalyse collective action and local institutions to diversify income-generating activities and develop health, sanitation and education infrastructure, as well as other key services^31^.

To assess the environmental and socio-economic effects of each type of protection arrangement versus alternative land uses, we compiled a high-spatial-resolution, longitudinal dataset of land use, deforestation and poverty for the period 2000–2010, the two most recent population census years for which microdata are available. We generate deforestation estimates from the well-established PRODES Brazilian Amazon deforestation dataset^32^ and combine these with four poverty indicators (fiscal income, income inequality, and metrics of literacy and sanitation) from the 2000 and 2010 population censuses for 5,545 census tracts (CTs) in the BLA. CTs are the smallest spatial unit for which socio-economic data are available^33^.

Our approach contrasts treated CTs that became protected by one of our three types of protection during our study period, with unprotected control CTs dominated by specific alternative land uses. Our socio-economic metrics capture data from human populations living across each CT, which, for protected CTs, will include some people legally residing within PA boundaries (even for some SPAs^34,35^) and people living close to PAs. We compiled property sizes of landholders from agricultural census data^36^ and approved mining concessions from the Sistema de Informações Geográficas da Mineração^37^ to define different subsets of comparable control CTs. Spatial data on PAs were obtained from the National Cadastre of Protected Areas from the Brazilian Ministry of Environment^38^.

Our principal analysis assesses differences in our key outcome variables following the establishment of PAs after 2000. Our study design does not include experimental manipulation (for example, a randomized control trial), so we are unable to make definitive causal claims about the statistical associations in our observational data. To maximize the robustness of our analysis, we use statistical matching and regression techniques, which control for potential biophysical, socio-economic and political confounders that could influence our outcomes and the establishment of PAs and ITs. We support our main findings and conclusions with nine robustness checks that include, among others, hidden bias tests to ensure results are not unduly influenced by missing confounders, different matching specifications, datasets and an alternative modelling framework (Supplementary Text 3).

## Results

### Comparisons with non-PAs

**Fig. 1:**
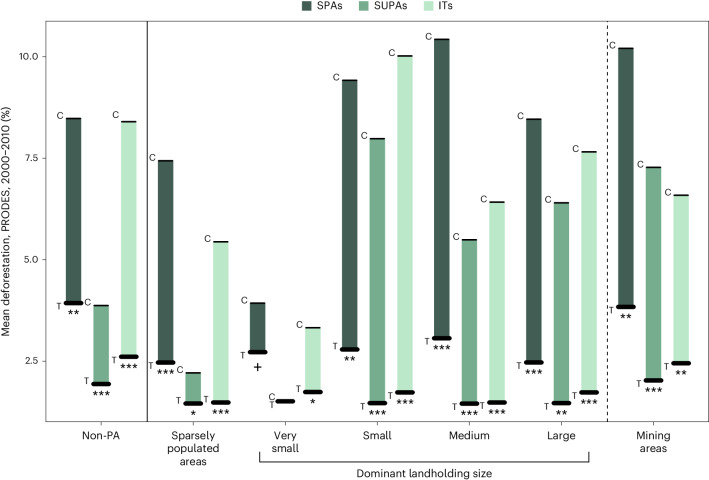
Differences in deforestation between PA types established after 2000 and alternative land uses. Non-PA controls include all the CTs with less than 1% of their land protected. Sparsely populated areas are those with less than 10% of the CT settled. Dominant agricultural landholder sizes are determined by the category containing the most properties per CT. Mining areas are defined as census tracts with licensed mining activities after 2000. Thick lines represent treated (T) CTs, and thin lines represent control (C) CTs. Significant differences (two sided) between treatment and matched controls: ***P < 0.001; **P < 0.01; *P < 0.05; ^+^*P* < 0.1. Exact *P* values and adjusted *P* values to correct for multiple testing are presented in Supplementary Table 1.

Throughout our results, we use SPAs, SUPAs and ITs to refer to census tracts protected after 2000. We first compared different forms of protection to the full set of control CTs (that is, without sub-setting controls into different land uses). We find that all forms of protection were associated with reductions in deforestation between 2000 and 2010, with ITs associated with the greatest amount of avoided deforestation (SPAs = −53.6%, *P* < 0.001; SUPAs = −46.7%, *P* < 0.001; ITs = −69.0%, *P* < 0.001; Fig. 1 and Supplementary Table 1).

**Fig. 2:**
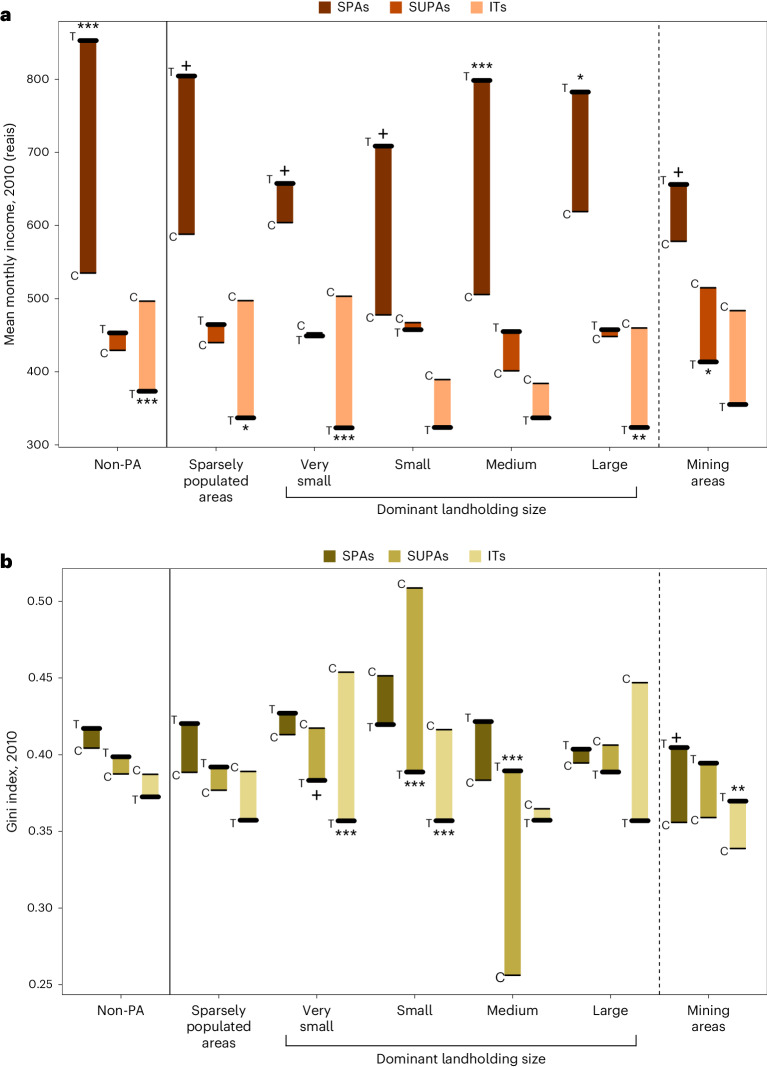
Differences in income and income inequality between PA types established after 2000 and alternative land uses. **a**, Effects on income, measured as the mean monthly income in Brazilian reais in CTs. **b**, Effects on income inequality measured as the Gini coefficient in CTs. Non-PA controls include all the CTs with less than 1% of their land protected. Sparsely populated areas are those with less than 10% of the CT settled. Dominant agricultural landholder sizes are determined by the category containing the most properties per CT. Mining areas are defined as CTs with licensed mining activities after 2000. Thick lines represent treated (T) CTs and thin lines represent control (C) census tracts. Significant differences (two sided) between treatment and matched controls: ***P < 0.001; **P < 0.01; *P < 0.05; ^+^*P* < 0.1. Exact *P* values and adjusted *P* values to correct for multiple testing are presented in Supplementary Tables 2 and 3.

**Fig. 3:**
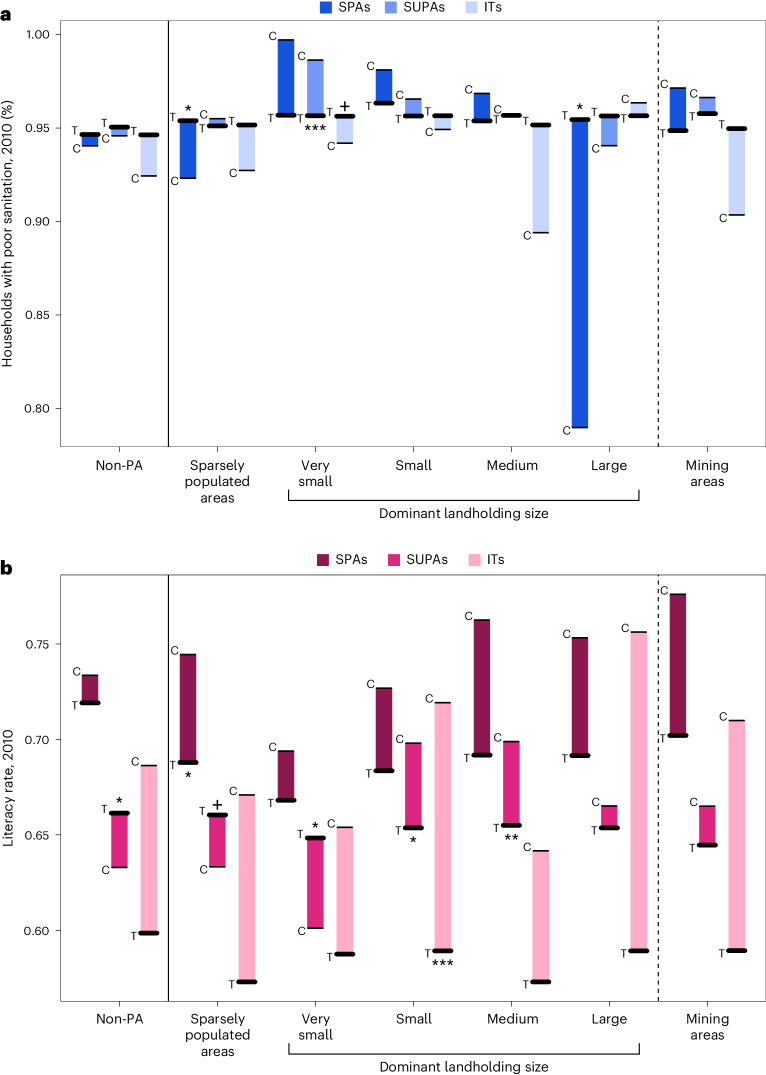
Differences in sanitation and literacy rates between PA types established after 2000 and alternative land uses. **a**, Effects on sanitation, measured as the percentage of households with poor sanitation in CTs. **b**, Effects on literacy, measured as literacy rates in CTs. Non-PA controls include all CTs with less than 1% of their land protected. Sparsely populated areas are those with less than 10% of the CT settled. Dominant agricultural landholder sizes are determined by the category containing the most properties per CT. Mining areas are defined as census tracts with licensed mining activities after 2000. Thick lines represent treated (T) CTs, and thin lines represent control (C) census tracts. Significant differences (two sided) between treatment and matched controls: ***P < 0.001; **P < 0.01; *P < 0.05; ^+^*P* < 0.1. Exact *P* values and adjusted *P* values to correct for multiple testing are presented in Supplementary Tables 4 and 5.

After being corrected for inflation, mean monthly household income increased during the study period in all types of protected CTs and unprotected controls. Increases were 59.3% (*P* < 0.001) higher in SPAs than in matched controls, with no significant difference in increases between SUPAs and controls; by contrast, increases in income in ITs were 24.8% (*P* < 0.001) lower than in matched controls (Fig. 2a and Supplementary Table 2). We found no relationship between any protection arrangement and income inequality (Fig. 2b and Supplementary Table 3) or sanitation (Fig. 3a and Supplementary Table 4). SUPAs were associated with slightly higher literacy rates than the matched controls (5.67% increase, *P* = 0.01), with other types of protection having no effect (Fig. 3b and Supplementary Table 5).

All protection arrangements thus appear effective at combatting deforestation while also improving socio-economic conditions over the study period. However, for ITs, improvements in income were below those in matched non-PAs, indicating environmental and socio-economic trade-offs. ITs were thus linked to the largest environmental gains but also the smallest socio-economic gains, suggesting that IPLCs may be missing out on economic development opportunities.

### Comparisons with agriculture and sparsely populated areas

SPAs and SUPAs were associated with reduced deforestation (between −40.5% and −81.6%) compared with agricultural land uses. This applied to all agricultural landholding sizes except when comparing SUPAs with areas dominated by very small landholders (SUPAs = −1.66%, *P* = 0.92; Fig. 1 and Supplementary Table 1). SPAs are also potentially less effective at reducing deforestation relative to areas dominated by small landholders, but our result is only marginally non-significant and the estimated effect size is much larger than that of SUPAs (SPAs = −30.7%, *P* = 0.08; Fig. 1 and Supplementary Table 1). By contrast, ITs were linked to significant reductions in deforestation compared with all five agricultural land uses (between 47.7% and 82.8% avoided deforestation; Fig. 1 and Supplementary Table 1).

SPAs were associated with larger increases in income relative to areas dominated by medium (57.9%, *P* < 0.0001) and large (26.4%, *P* = 0.03; Fig. 2a and Supplementary Table 2) agricultural landholders. By contrast, ITs were linked to smaller increases in income compared with sparsely populated areas (−32.2%, *P* = 0.02; Fig. 2a and Supplementary Table 2) and areas dominated by very small (−35.8%, *P* < 0.0001; Fig. 2a and Supplementary Table 2) and large landholders (−29.6%, *P* = 0.01; Fig. 2a and Supplementary Table 2). We find no relationship between SUPAs and income relative to any agricultural landholding size.

SUPAs were associated with significant reductions in income inequality when compared with areas dominated by small landholders (−12.3%, *P* < 0.001; Fig. 2b and Supplementary Table 3), but were linked to significant increases in inequality when compared with areas dominated by medium landholders (59.1%, *P* < 0.001; Fig. 2b and Supplementary Table 3). ITs were associated with significant reductions in income inequality when compared with areas dominated by very small (−21.4%, *P* < 0.0001; Fig. 2b and Supplementary Table 3) and small landholders (−14.3%, *P* < 0.001; Fig. 2b and Supplementary Table 3). SPAs had no significant relationship with income inequality.

SPAs were associated with significantly smaller increases in literacy rates when compared with sparsely populated areas (−7.59%, *P* = 0.03; Fig. 3b and Supplementary Table 5). SUPAs were linked to smaller increases in literacy when compared with areas dominated by small (−8.49%, *P* = 0.01; Fig. 3b and Supplementary Table 5) and medium landholders (−13.9%, *P* = 0.01; Fig. 3b and Supplementary Table 5) but were associated with greater increases in literacy compared with very small landholders (1.98%, *P* = 0.01; Fig. 3b and Supplementary Table 5). ITs were linked to smaller increases in literacy rates when compared with areas dominated by small landholders (−18.1%, *P* < 0.001; Fig. 3b and Supplementary Table 5).

SPAs were associated with lower improvements in sanitation compared with sparsely populated areas and areas dominated by large landholders (sparsely populated areas = 3.34%, *P* = 0.03; large landholders = 20.9%, *P* = 0.03; Fig. 3a and Supplementary Table 4). By contrast, SUPAs were linked to greater improvements in sanitation when compared with areas dominated by very small landholders (−2.71%, *P* < 0.001; Fig. 3a and Supplementary Table 4).

These results emphasize the importance of ITs in combatting deforestation, as they effectively reduced deforestation compared with all forms of agricultural land use. The effectiveness of SUPAs, and potentially SPAs, in reducing deforestation relative to areas dominated by very small landholders is limited. These results also show potentially contrasting socio-economic effects depending on the type of protection and the size of agricultural landholdings in the comparison. Critically, although ITs are sometimes associated with lower levels of inequality, Indigenous communities may be missing out on income-generating opportunities, especially compared with SPAs that reduced deforestation while maintaining or even increasing average incomes within the CT compared with alternative agricultural land uses.

### Comparisons of different forms of protection to mining

All forms of protection were associated with reduced deforestation compared with CTs with licensed mines (SPAs = −62.4%, *P* < 0.001; SUPAs = −76.5%, *P* < 0.001; ITs = −62.9%, *P* < 0.001). SUPAs were associated with economic trade-offs with significantly lower gains in income than in mining areas (−9.88%, *P* = 0.04). ITs were linked to significant increases in income inequality (9.13%, *P* = 0.01) relative to CTs with licensed mines. We find no relationship between protection and literacy rates or sanitation compared with mining areas. These results suggest that SPAs can prevent the adverse environmental impacts of mining without adversely affecting socio-economic conditions but provide further evidence for environmental and socio-economic trade-offs for ITs and SUPAs.

### Robustness checks

**Extended Data Fig. 1:**
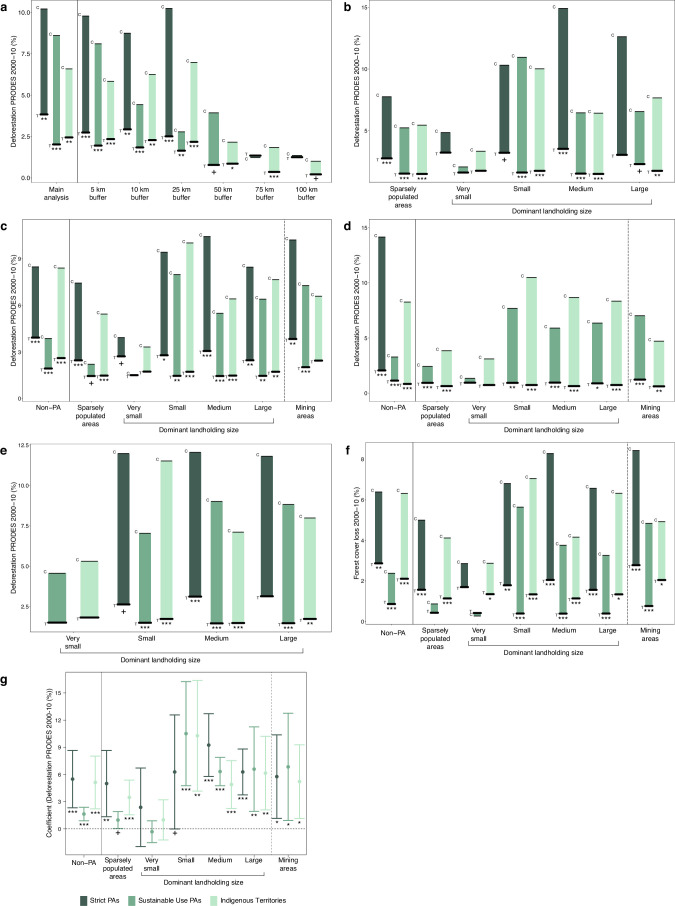
Deforestation effects of Strict Protected Areas (SPAs), Sustainable Use Protected Areas (SUPAs), and Indigenous Territories (ITs). **a** Effects of protected areas and Indigenous Territories established after 2000 in comparison to mining at buffers up to 100 km. **b** Effects of protected areas and Indigenous Territories established after 2006 (the date of the agricultural census). **c** Effects of protected areas and Indigenous Territories established after 2000 using 1.5 calipers to improve balance. **d** Protected area and Indigenous Territory effects after raising the protection threshold from 10% to 50% of census land area (using the PRODES deforestation dataset). **e** Effects of protected areas and Indigenous Territories established after 2000 in comparison to landholdings dominated by pastures. **f** Effects of protected areas and Indigenous Territories established after 2000 using Hansen et al’s high-resolution global deforestation maps^37^. **g** Fixed effects panel regression results (data are represented as coefficient values and error bars represent 95% confidence intervals calculated using heteroscedasticity robust standard errors, see Table S16 for sample sizes for each comparison and P-values corrected for multiple testing). Controls include all census tracts (CTs) with less than 1% of their land protected. Sparsely populated areas are defined as areas with less than 10% of the CTs settled. Dominant agricultural landholder size is determined by the category that contains the most properties per CT. Mining sites are defined as CTs with recorded licensed mining activities present after 2000. Thick lines represent treatment (T) CTs that are protected, and thin lines represent matched controls (C). Significant differences (two-sided) between treatment and matched controls: *** P < 0.001, ** P < 0.01, * P < 0.05, + P < 0.1. Exact P-values are presented in Table S16.

**Extended Data Fig. 2:**
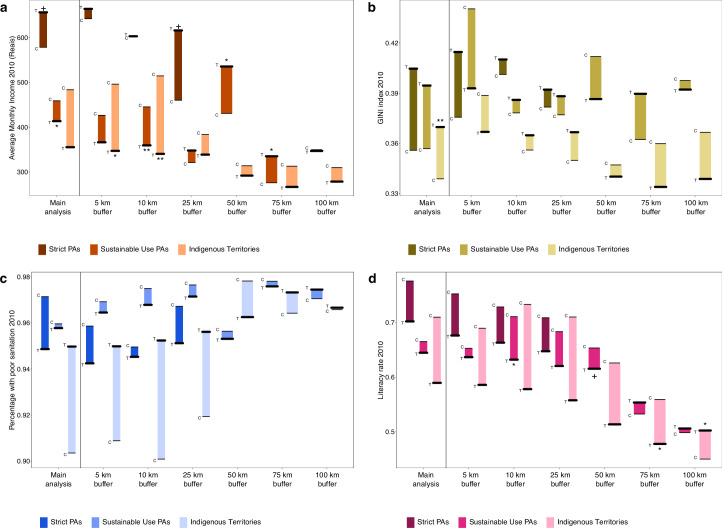
Socioeconomic effects of Strict Protected Areas (SPAs), Sustainable Use Protected Areas (SUPAs), and Indigenous Territories (ITs) in comparison to mining concessions across different buffer distances. **a** Effects on mean monthly income in census tracts (CTs). **b** Income inequality effects, measured as the GINI coefficient in CTs. **c** Effects on sanitation, measured as the percentage of households with poor sanitation in CTs. **d** Effects on literacy, measured as literacy rates in CTs. Controls include all CTs with less than 1% of their land protected. Sparsely populated areas are defined as areas with less than 10% of the CTs settled. Dominant agricultural landholder size is determined by the category that contains the most properties per CT. Mining sites are defined as CTs with recorded licensed mining activities present after 2000. Thick lines represent treatment (T) CTs that are protected, and thin lines represent matched controls (C). Significant differences (two-sided) between treatment and matched controls: *** P < 0.001, ** P < 0.01, * P < 0.05, + P < 0.1. Exact P-values are presented in Tables S17–S20.

**Extended Data Fig. 3:**
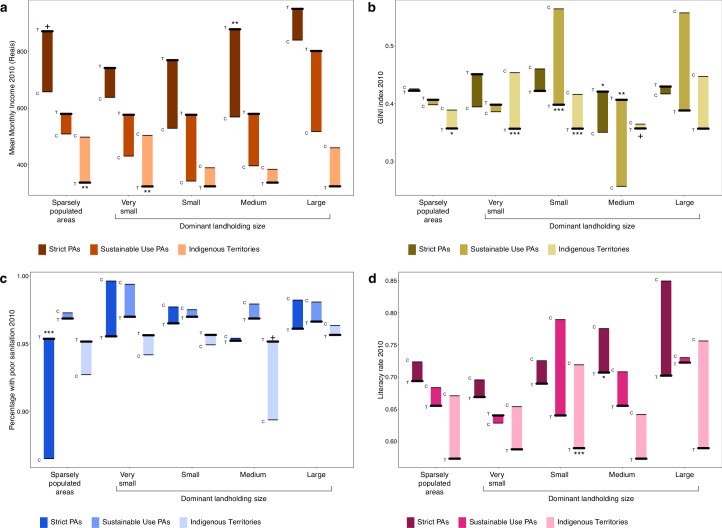
Socioeconomic effects of Strict Protected Areas (SPAs), Sustainable Use Protected Areas (SUPAs), and Indigenous Territories (ITs) established after 2006, the year the agricultural census was conducted. **a** Effects on mean monthly income in census tracts (CTs). **b** Income inequality effects, measured as the GINI coefficient in CTs**. c** Effects on sanitation, measured as the percentage of households with poor sanitation in CTs. **d** Effects on literacy, measured as literacy rates in CTs. Controls include all CTs with less than 1% of their land protected. Sparsely populated areas are defined as areas with less than 10% of the CTs settled. Dominant agricultural landholder size is determined by the category that contains the most properties per CT. Mining sites are defined as CTs with recorded licensed mining activities present after 2000. Thick lines represent treatment (T) CTs that are protected, and thin lines represent matched controls (C). Note that comparisons with non-protected areas and mining areas are excluded, because the main results are not affected by the agricultural census. Significant differences (two-sided) between treatment and matched controls: *** P < 0.001, ** P < 0.01, * P < 0.05, + P < 0.1. Exact P-values are presented in Tables S17–S20.

**Extended Data Fig. 4:**
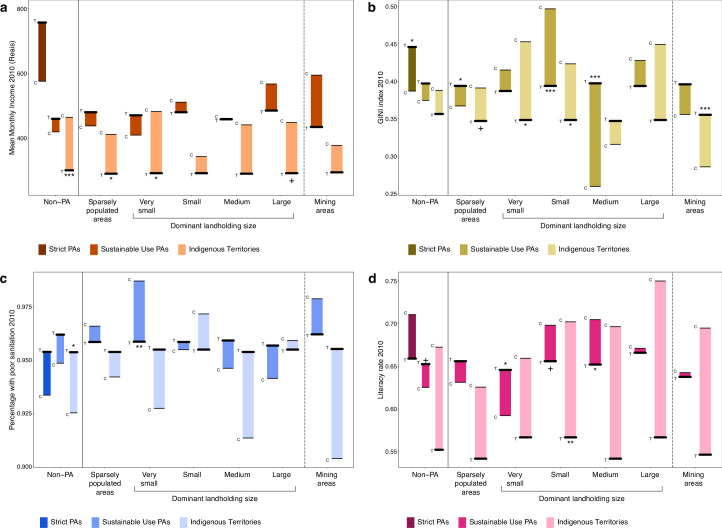
Socioeconomic effects of Strict Protected Areas (SPAs), Sustainable Use Protected Areas (SUPAs), and Indigenous Territories (ITs) after raising the threshold for treatment allocation from 10% to 50% cover of a census tract. **a** Effects on mean monthly income in census tracts (CTs). **b** Income inequality effects, measured as the GINI coefficient in CTs. **c** Effects on sanitation, measured as the percentage of households with poor sanitation in CTs. **d** Effects on literacy, measured as literacy rates in CTs. Controls include all CTs with less than 1% of their land protected. Sparsely populated areas are defined as areas with less than 10% of the CTs settled. Dominant agricultural landholder size is determined by the category that contains the most properties per CT. Mining sites are defined as CTs with recorded licensed mining activities present after 2000. Thick lines represent treatment (T) CTs that are protected, and thin lines represent matched controls (C). Significant differences (two-sided) between treatment and matched controls: *** P < 0.001, ** P < 0.01, * P < 0.05, + P < 0.1. Exact P-values are presented in Tables S17–S20.

**Extended Data Fig. 5:**
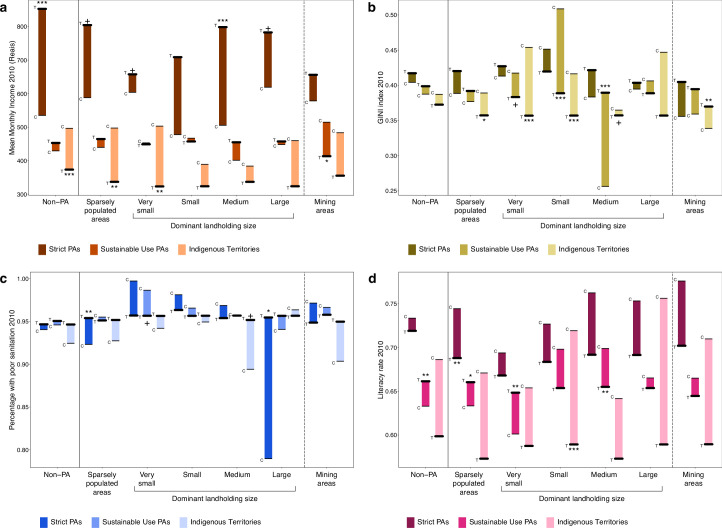
Socioeconomic effects of Strict Protected Areas (SPAs), Sustainable Use Protected Areas (SUPAs), and Indigenous Territories (ITs) after using a 1.5 caliper to improve balance. **a** Effects on mean monthly income in census tracts (CTs). **b** Income inequality effects, measured as the GINI coefficient in CTs**. c** Effects on sanitation, measured as the percentage of households with poor sanitation in CTs. **d** Effects on literacy, measured as literacy rates in CTs. Controls include all CTs with less than 1% of their land protected. Sparsely populated areas are defined as areas with less than 10% of the CTs settled. Dominant agricultural landholder size is determined by the category that contains the most properties per CT. Mining sites are defined as CTs with recorded licensed mining activities present after 2000. Thick lines represent treatment (T) CTs that are protected, and thin lines represent matched controls (C). Significant differences (two-sided) between treatment and matched controls: *** P < 0.001, ** P < 0.01, * P < 0.05, + P < 0.1. Exact P-values are presented in Tables S17–S20.

**Extended Data Fig. 6:**
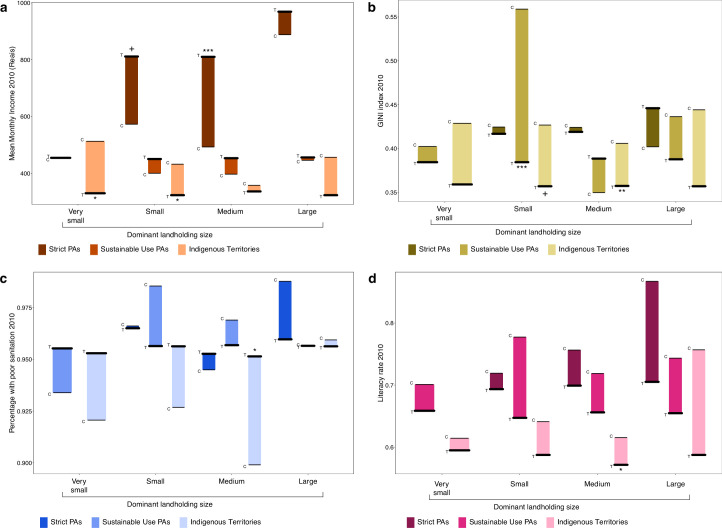
Socioeconomic effects of Strict Protected Areas (SPAs), Sustainable Use Protected Areas (SUPAs), and Indigenous Territories (ITs) in comparison to agricultural landholding areas dominated by pastures. **a** Effects on mean monthly income in census tracts (CTs)**. b** Income inequality effects, measured as the GINI coefficient in CTs. **c** Effects on on sanitation, measured as the percentage of households with poor sanitation in CTs. **d** Effects on literacy, measured as literacy rates in CTs. Controls include all CTs with less than 1% of their land protected. Sparsely populated areas are defined as areas with less than 10% of the CTs settled. Dominant agricultural landholder size is determined by the category that contains the most properties per CT (very small: <10 ha, small: 10–50 ha, medium: 50–200 ha, large: >200 ha). Mining sites are defined as CTs with recorded licensed mining activities present after 2000. Thick lines represent treatment (T) CTs that are protected, and thin lines represent matched controls (C). Significant differences (two-sided) between treatment and matched controls: *** P < 0.001, ** P < 0.01, * P < 0.05, + P < 0.1. Exact P-values are presented in Tables S17-S20.

**Extended Data Fig. 7:**
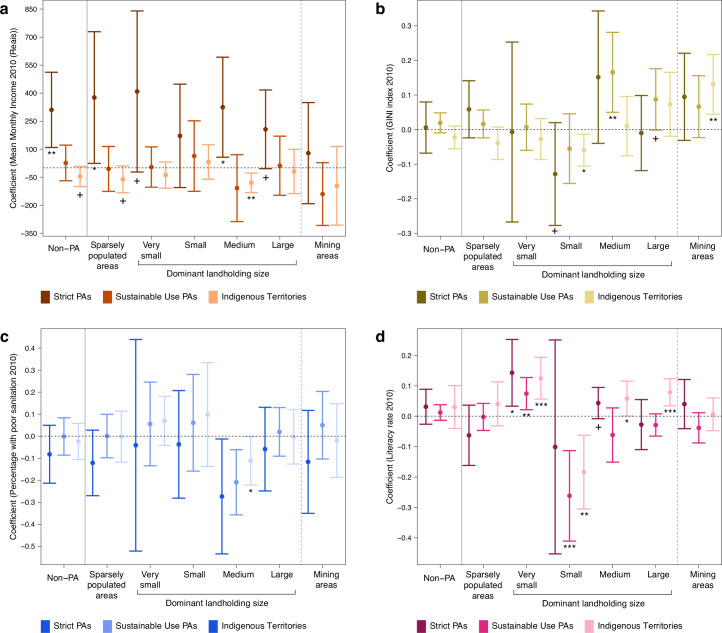
Socioeconomic effects of Strict Protected Areas (SPAs), Sustainable Use Protected Areas (SUPAs), and Indigenous Territories (ITs) using fixed effects panel models. **a** Effects on mean monthly income in census tracts (CTs). **b** Income inequality effects, measured as the GINI coefficient in CTs**. c** Effects on sanitation, measured as the percentage of households with poor sanitation in CTs. **d** Effects on literacy, measured as literacy rates in CTs. Controls include all CTs with less than 1% of their land protected. Sparsely populated areas are defined as areas with less than 10% of the CTs settled. Dominant agricultural landholder size is determined by the category that contains the most properties per CT. Mining sites are defined as CTs with recorded licensed mining activities present after 2000. Data are represented as coefficient values and error bars represent 95% confidence intervals calculated using heteroscedasticity robust standard errors. Significant differences (two-sided) between treatment and matched controls: *** P < 0.001, ** P < 0.01, * P < 0.05, + P < 0.1. Exact P-values and P-values corrected for multiple testing are presented in Tables S17–S20.

**Extended Data Fig. 8:**
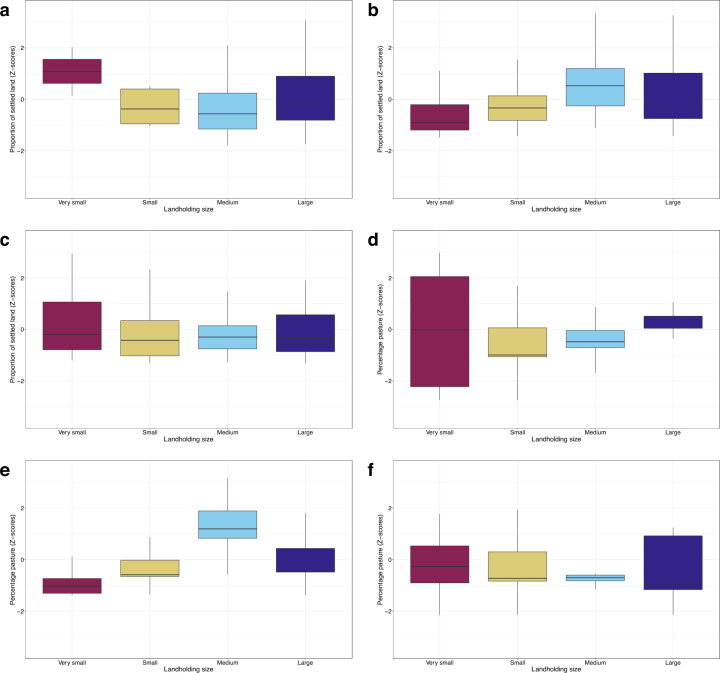
Differences between control census tracts, dominated by different landholding sizes, in the amount of settled land (a-c) and pasture land (d-e) - assessed using (two-sided) ANOVAs. **a** Settled land in matched control census tracts (CTs) used to analyse Strict Protected Area (SPA) effects (F = 0.15, df = 3, P = 0.93). **b** Settled land in matched control CTs used to analyse Sustainable Use Protected Area (SUPA) effects (F = 0.67, df = 3, P = 0.57). **c** Settled land in matched control CTs used to analyse Indigenous Territory (IT) effects (F = 0.32, df = 3, P = 0.81). **d** Pasture land in matched control CTs used to analyse SPA effects (F = 0.05, df = 3, P = 0.98). **e** Pasture land in matched control CTs used to analyse SUPA effects (F = 0.42, df = 3, P = 0.74). **f** Pasture land in matched control CTs used to analyse IT effects (F = 0.41, df = 3, P = 0.73). Dominant landholder size is determined by the category that contains the most properties per CT (very small: <10 ha, small: 10–50 ha, medium: 50–200 ha, large: >200 ha). Box boundaries represent upper and lower quantiles, horizontal lines inside the box represent Z-scores, vertical lines represent range.

We conducted a suite of different robustness checks (Supplementary Text 3 and Extended Data Figs. 1–8): (1) stricter matching criteria (calipers), (2) stricter spatial definitions of protection (raising the threshold for defining protected CTs from 10% PA or IT coverage in our main analysis to 50%), (3) restricting our analysis to deforestation from 2006 to 2010 and PAs that were established after 2006, (4) examining the potential presence of unmeasured confounders by conducting hidden bias sensitivity analyses (Oster bounds) and testing for spatial autocorrelation in our regression models, (5) ensuring that statistical differences between landholding size are not driven by differing amounts of settled land, (6) ensuring that our results are consistent across agricultural land dominated by different agricultural activities (for example, pasture), (7) using the high-resolution global deforestation maps generated by a previous study^39^ as an alternative dataset to PRODES, (8) examining the spatial extent of potential mining effects and (9) using an alternative modelling framework (fixed-effects panel regression). These tests confirm that our analyses are not adversely influenced by hidden bias from unmeasured confounders and validate the conclusions that we draw from our main results.

## Discussion

We make two important contributions to our understanding of the effects of different protection arrangements. First, we add to our understanding of the joint environmental and socio-economic outcomes of different protection arrangements and their trade-offs. Second, we contribute to the advancement of evaluation frameworks that assess protection outcomes relative to alternative land uses.

We find that across all analyses, ITs are the governance arrangement most consistently associated with reductions in deforestation relative to agricultural land use and mining in our study period. These results highlight the conservation benefits that can arise from recognizing the rights of IPLCs to natural resources in Brazil and potentially other regions^40^. However, our results also suggest that ITs are more likely to experience socio-economic trade-offs than other protection arrangements. ITs were associated with smaller increases in income and, in some instances, lower literacy rates, compared with other land uses. Indigenous communities in Brazil and elsewhere are often more autarkic (for example, because of reduced access to markets and other institutions; Supplementary Information). Yet, as global efforts to return land and resource rights to IPLCs ramp up in the wake of the 2021 United Nations Climate Change Conference (COP 26; Glasgow Leaders’ Declaration on Forests and Land Use), it is critical that interventions to secure land rights are accompanied by targeted development programmes and initiatives designed to remove access barriers to other forms of support (for example, existing social protection programmes^41^).

SUPAs in the BLA appear not to be effective at reducing deforestation compared with areas dominated by very small (<10 ha) landholdings (which comprise a similar proportion of the BLA as agricultural land dominated by other landholding size categories; Supplementary Fig. 1a). These results point to the current limited environmental gains of designating SUPAs in areas dominated by small-scale agriculture. However, the history of the BLA highlights the risk of the consolidation of smaller landholdings by larger commercial agricultural enterprises (Supplementary Text 1). Designating SUPAs could thus reduce the risk of such consolidation and be more effective when considering future land use transitions. These dynamics should be explicitly considered in efforts to increase PA networks in both the BLA and beyond.

More importantly, we find that the environmental gains from SPAs and SUPAs rarely trade off against socio-economic development when compared with agricultural land uses, including areas dominated by large agricultural landholdings. This result is compatible with suggestions that the socio-economic benefits of large agricultural business development in the BLA can be limited for large proportions of the rural population^11^. Our study thus indicates that agricultural business development of the BLA is unlikely to provide socio-economic benefits for local people to a greater extent than protection-focused alternatives that preserve forest cover.

Our results suggest that all protection arrangements are effective at reducing deforestation arising from mining land uses (although we are able to assess only legal mining concessions and note that illegal mining can affect deforestation and communities both outside and within ITs and PAs). This effect extends up to 50 km from mining sites for SUPAs and up to 75 km for ITs (Extended Data Fig. 1a). However, results suggest that this may involve socio-economic trade-offs in the case of SUPAs and ITs. ITs experienced greater increases in inequality relative to mining, and there are indications that SUPAs experienced smaller increases in income. Critically, these negative socio-economic effects extended over much smaller distances (5–10 km; Extended Data Fig. 2) than the positive environmental effects of protection, that is, reduced deforestation rates. This emphasizes the importance of considering spatial scale in balancing trade-offs between environmental protection and socio-economic development goals.

Our approach reveals important heterogeneity in the environmental and socio-economic outcomes of protection arrangements in the BLA and provides a better understanding of synergies and trade-offs across sustainability objectives that arise from alternative land use decisions. The magnitude to which the outcomes of protection arrangements extend beyond the boundaries of ITs and PAs, and whether protection displaces deforestation to other locations in the BLA (and elsewhere), remain an important research frontier. Considering alternative land use options in future evaluations of PAs and other effective area-based conservation measures can further help us better understand socio-environmental trade-offs with important implications for conservation policy and practice.

The target of protecting 30% of terrestrial land surface by 2030 has been enshrined in the United Nations Convention on Biological Diversity^1^. As plans develop to meet the 30 × 30 target, it is critical that the expansion of protection efforts is not biased to areas with limited additionality, where opportunity costs are lower but conservation effectiveness is reduced. Agriculture and mining frequently compete against efforts to protect natural habitats and the provision of rights to rural communities^9,12,22^. Gaining a better understanding of socio-economic and environmental synergies and trade-offs in other regions and contexts, and the specific processes through which these occur (for example, changes in local wages^42^), will be essential for better policy design.

## Methods

We first compare deforestation and multiple socio-economic outcomes (fiscal income, income inequality, literacy and sanitation) between three different types of protection arrangements (SPAs, SUPAs and ITs) and matched non-PAs from the full set of control CTs. We then compare the outcomes of these three protection treatments to control CTs dominated by specific alternative land uses (sparsely populated areas, areas dominated by different agricultural landholding sizes and mining).

### Unit of analysis

Our analysis focuses on 5,545 CTs (mean size ± s.e., 977 ± 44.7 km^2^; Supplementary Fig. 1b) within the BLA derived from CTs identified in Brazil’s 2000 human population census. Focusing on CTs rather than municipalities (of which there are 772 in the BLA as of 2021^43^) allowed us to assess patterns at finer spatial scales and substantially increase our sample size and thus the statistical power of our analysis. We merged CTs that were smaller than 50 km^2^ (mean size ± s.e., 3.5 ± 0.04 km^2^; total area covered is less than 1% of the BLA area) as the boundaries of these CTs are tightly delimiting settled areas (villages) from the surrounding rural landscape. This merging of CTs increases the ability to detect impacts of land use decisions on the residents of these small CTs. Our algorithm selected the smallest CT, then merged it with the neighbouring CT situated in the same municipality with whom it shared the longest border—although, in practice, most of the small census tracts are surrounded by much larger census tracts. The algorithm repeated this procedure until the area of the smallest remaining CT was larger than 50 km^2^.

The boundaries of some census tracts changed between 2000 and 2010. We thus followed an approach^44^ that assumes that people are homogeneously distributed within the census tract. We consistently reconstructed data to the 2000 CT boundaries by first overlapping the 2010 CT boundaries with the 2000 CT boundaries and then calculating a weighted average of the 2010 metric for the 2000 CT boundary. Agricultural census data also experienced boundary changes between 2000 and 2006, and the same procedure was followed to calculate a 2006 metric allocated to the 2000 CT boundaries.

### Outcomes

#### Forest cover loss metric

We used the Brazilian PRODES^29^ to estimate forest cover loss for our main analysis. The PRODES dataset is used nationally and internationally in the assessment of deforestation in the Amazon^45^ and quantifies cumulative deforestation in patches of at least 6.25 ha. PRODES detects only loss of old-growth forest and ignores secondary forest gains and losses, including those associated with shifting cultivation systems (see Supplementary Text 3 for a robustness test using the data of a previous study^39^, which measures deforestation at 30 m resolution).

#### Poverty metrics

We use four indicators of poverty and well-being available in the national census microdata, which aggregates individual household data at the CT level: (1) mean household income per month in Brazilian reais (R$), corrected for inflation using the Índice Nacional de Preços ao Consumidor (INPC)^46^; (2) income inequality, measured as the Gini coefficient and calculated at the household level (for each CT) using data on the numbers of households within each income category; (3) literacy rates as a measure of education, calculated as the percentage of literate household heads (parental education is a positive indicator of child education in Global South countries and a good indicator of social mobility^47^) and (4) percentage of households with poor sanitation (that is, without toilets that drain into a sewage system or septic tank).

We focus on these four indicators for several reasons. Income is considered a key measure of poverty and well-being in global sustainability agendas (for example, Sustainable Development Goal 1, Target 1.1) and is used by Brazilian state agencies to measure poverty^33^ and eligibility for socio-economic protection programmes (for example, Bolsa Familia^48^). Similarly, reducing income inequality is also considered a key sustainability goal and target (Sustainable Development Goal 10, Target 10.1). In addition, there is widespread recognition that poverty and well-being are multi-dimensional issues. We therefore also include two variables available in the census data: literacy (as a proxy of education) and sanitation (as a proxy of health). Our indicators thus cover a wide range of nationally and globally relevant poverty and well-being measures that are also closely linked to commonly used measures to assess PA impacts in other contexts^44,49^. More importantly, our indicators probably capture changes in dimensions of poverty and well-being of large swathes of the rural population of the BLA, including populations in ITs. This is because, although more autarkic and often marginalized politically and economically, Indigenous peoples in the BLA (1) do engage in market economies (at least to some extent), (2) are eligible for socio-economic protection programmes and receive conditional cash transfers (despite structural difficulties in accessing them) and (3) have constitutional rights to bilingual education (although these rights are often only partially met)^50^.

### Intervention and control definitions

#### Defining interventions

We define each of our three protection arrangement types and their locations in the BLA using the Brazilian National Protected Area System (SNUC)^51^ (Supplementary Fig. 1c). We define SPAs as the five categories of Integral Protected Units (Unidades de Proteção Integral), that is, ecological research stations, biological reserves, national parks, natural monuments and wildlife reserves. Environmental protection is the principal aim of each of these five categories, and they equate to IUCN PA categories I to IV. Resource use in these areas is prohibited, but they can be accessed for research, education and, in some instances, tourism (for example, in national parks). The Brazilian National Protected Area System defines seven categories of PAs within the sustainable use group, each with a specific set of use and occupation rules. The SUPAs are, from the least to the most restrictive, environmental protection areas (APA), areas of relevant ecological interest (ARIE), national or state forests (FLONA or FLOES), extractivist reserves (ResEx), fauna reserves (RF), sustainable development reserves (RDS) and natural heritage private reserves (RPPN). All categories are represented in the BLA, except for RFs, which were never implemented anywhere in the country^34^. For this study, we define SUPAs as FLONAs and FLOES, ResEx and RDS (all of which equate to IUCN PA categories V and VI). APAs and ARIEs were not included because they represent very large areas that encompass multiple landscape contexts (for example, urban, mining, agriculture, other PAs) and are subject to zoning rules instead of rules that apply throughout the PA, and they do not equate to IUCN PA categories. Natural heritage private reserves (RPPNs) were not included because they are privately owned and cover only approximately 4% of the BLA (mostly in the state of Mato Grosso)^52^. Critically, although their restrictive-use rules can resemble those of full-protection categories, the mechanisms through which private protected areas can influence broader socio-economic outcomes remain unclear^53^. The 1988 Brazilian Constitution provided inalienable rights to land for Indigenous peoples. Indigenous peoples in Brazil face continued social and economic injustices, yet the 20 years between 1990 and 2010 saw the largest declaration (725,531 km^2^) and homologation (903,260 km^2^) of Indigenous territories in the Legal Amazon region^54^. Population densities within SPAs and SUPAs are low compared with those of immediately adjacent areas^34^, but residence within PA boundaries is legally permitted in some SPA and SUPA categories (for example, wildlife reserves and RDS)^55^.

To make our assessments more conservative, we excluded CTs overlapping other protection arrangements than the one of interest; for example, when analysing SUPAs, we removed CTs that also overlapped an SPA or IT. We define CTs as protected when protection was established between our baseline (2000) and endline (2010) years; note that we conduct a robustness check focusing only on protection after 2006 (the year of the agricultural census) that limits the variation in the timing of protection establishment (Supplementary Text 3 and Extended Data Figs. 1b and 3). We defined CTs as being protected based on the proportion of the CT that overlapped an SPA, SUPA or IT. For our main analysis, we use CTs with at least 10% of their area overlapping a protection arrangement due to the frequent use of this threshold in other studies^44^, although percentage overlap was generally higher (Supplementary Fig. 2a–c; see Extended Data Figs. 1d and 4 for a robustness test using CTs with at least 50% of the census tract overlapping with protection, a higher threshold that is also used in other studies^24^). We defined CTs as being not protected when <1% of their area was protected. We thus excluded CTs with more than 1%, but less than 10%, overlap with a protection arrangement to ensure a clear distinction in the amount of protection between control and treatment CTs.

#### Choice of control land uses

We first compare deforestation and poverty outcomes of protected CTs to matched non-protected CTs. We then compared differences in outcomes between each type of protection arrangement (SPAs, SUPAs and ITs) and a subset of control CTs dominated by specific alternative land uses (sparsely populated areas, areas dominated by four classes of agricultural landholding size and mining). We focus on agriculture and mining because they are globally key competitors with PAs for land. Moreover, they are the two major drivers of deforestation in the BLA^18,56^. We did not define forest concessions as an additional type of alternative land use owing to their rarity in the BLA^57^. We did, however, classify unprotected CTs with no licenced mines and less than 10% of their area occupied by agricultural areas as sparsely populated areas as an additional alternative land use.

#### Defining controls

##### Agriculture

First, we compared differences in outcomes between protection arrangements and agricultural areas. We defined a CT as an agricultural control if at least 10% of the CT was settled by agricultural properties, although in most instances the proportion of area settled by different landholding CTs was substantially higher (Supplementary Fig. 2d–g). To ensure a contrast between protected CTs and agricultural control CTs, we excluded protected CTs that also incorporated more than 10% settled land from this part of the analysis. Following a previous study^10^, we defined agricultural controls based on the property size of landholdings. We calculated the dominant landholding size for each CT from the 2006 Brazilian agricultural census^36^ and used these data for the entire 2000–2010 study period. This is an inevitable shortcoming due to data availability, but we believe that the 2006 survey data are sufficient to analyse spatial heterogeneity in landholder distribution in the Amazon. Indeed, we found qualitatively identical patterns to our main analysis when we assessed how PAs established after 2006 influenced deforestation rates between 2000 and 2010 across the gradient in landholding size (Extended Data Fig. 1b).

We determined which landholder size category (very small, small, medium and large landholders) dominated individual census tracts by identifying which landholder size category had the most properties per census tract. This approach ensures that our categorization reflects the type of landowner that contributes most to our census-based poverty metrics (Supplementary Fig. 1a). We classified properties smaller than 10 ha as ‘very small’, properties between 10 ha and 50 ha as ‘small’, properties between 50 ha and 200 ha as ‘medium’ and properties bigger than 200 ha as ‘large’. This classification largely follows global assessments of farm size^58^, but is adjusted for the Brazilian Amazon because farm sizes in Latin America are generally bigger than the global average. Our classification can tease apart differences in agricultural property size at lower scales than previous assessments conducted in the Amazon that generally treat all properties smaller than 100 ha as smallholder agriculture^59^. Teasing apart differences in property size below this threshold could be important because 100 ha is substantially higher than the size of the average family farm in Brazil (18.37 ha)^60^ and the BLA (57 ha)^61^.

After matching, we use analyses of variance to confirm that our control CTs dominated by different agricultural landholding sizes are similar in their amount of settled land (Extended Data Fig. 8a–c). We conduct a similar post-matching analysis to ensure that the amount of pasture land does not differ between different landholding sizes (Extended Data Fig. 8d–f).

##### Mining

Our approach for comparing protection arrangements to mining areas follows the same approach as our comparison to agricultural areas. We followed previous studies^15^ and classified areas influenced by mining as sites with mines classified in the Sistema de Informações Geográficas da Mineração that have been officially approved and licensed for mining by the Brazilian Ministry of Mines and Energy^37^. These categories included ‘concessão de lavra’, ‘concessão de lavra garimpeira’, ‘licenciamento’, and ‘registro de extração’. We identified mining controls based on the presence and absence of at least one licensed mine within the CT (Supplementary Fig. 1d). When comparing protected CTs with unprotected CTs with mining, we ensure that all CTs used in our analysis lack mines at baseline (2000). Thus, unprotected CTs with mining are those in which mining commenced after 2000, and protected CTs lack mines throughout the treatment period. We excluded mines established at baseline because they could have differential impacts to recently licensed mines. In many cases, infrastructure development occurs around the time of licensing and it is this infrastructure development that influences deforestation rates^18^. For this part of the analysis, we also excluded protected CTs with mines established after 2000 to ensure that control and treatment groups differ in the occurrence of mines. Note that the number of protected CTs with mining expansion is very small (two CTs protected by ITs also have mines within the boundaries of the CT; no CTs protected by SUPAs have mines and six CTs protected by SPAs have mines). Due to small sample sizes and insufficient variation, and following ref. ^18^, we did not differentiate between different types of mines that vary in their size or nature of the mined resources (for example, gold mining or other polymetallic mines). Deposits for these mined materials are widely distributed across the BLA and overlap substantially with locations with protection arrangements^62^. There is considerable interest in exploiting these resources. For example, even though mining is not currently allowed in ITs, the Brazilian National Mining Agency has received over 3,500 requests to mine in the lands of 90 isolated Indigenous groups^63^—we thus consider it feasible that mining could occur across protected CTs.

### Confounders

We selected biophysical and socio-economic confounders based on their potential to influence the outcome or the relationship between treatment and outcomes^22^. These confounders were baseline levels of our poverty measures, size of the CT, baseline forest cover, indicators of suitability for agriculture and mining development (slope, elevation, flood risk and travel time from the census tract to major cities (population size > 50,000)), population density, mines established before baseline and state (see Supplementary Text 2 and Supplementary Table 6 for data sources and justification, Supplementary Table 7 for confounders included in different analyses and Supplementary Tables 8–10 for summary statistics).

For our analyses comparing protection arrangements to agricultural CTs and sparsely populated areas, in addition to matching on mines established before our baseline, we also matched on mines established during our study period. We do this to control for mining activities that could confound our comparison between protection and agriculture. Similarly, we include the proportion of settled land as a covariate to isolate the impacts of PAs relative to mining in our analysis comparing the effects of PAs with mining. We do this to control for agricultural activities that could confound our comparison between protection and mining. The individual covariates included in our different comparisons are included in Supplementary Table 7.

### Matching

We used a combined matching and ordinary least square (OLS) regression approach to assess the differences in poverty and deforestation outcomes between PAs, agriculture and mining. Combining matching and regressions is a well-developed method for assessing PA outcomes^64^. Matching is a data pre-processing technique to balance covariate distributions across treated and control units and is useful when imbalance between treatment and control hinders causal inference^65^. We used full matching for all covariates, except state and mining for which we used exact matching, and used post-matching standardized mean differences of <0.25 as an acceptable balance between treatment and control groups for each covariate^66^. We used the ‘MatchIt’ package in R for all our matching analyses. Following a previous study^67^, we iteratively removed covariates from the propensity score model to optimize balance across all covariates in our theory of change. Once balance was optimized, we included all covariates in our theory of change in our final post-matching regression models (Supplementary Table 7).

We used full matching^68^ because it allows us to maximize our sample sizes, particularly the number of CTs that meet our requirements for inclusion as control units. Compared with matching with replacement, full matching maximizes the use of the data by generating subclasses in which each subclass contains one treated unit matched to one or more control units, or one control unit matched to one or more treatment units. However, to confirm that full matching is an appropriate choice, we compared the full matching and genetic match balance results of our worst matched comparisons (that is, where average post-matching standardized mean differences across unbalanced (>0.25) confounders was highest). We found that full matching outperformed genetic matching in all instances (Supplementary Fig. 3).

Full matching substantially improved the balance of our confounders across all our analyses (Supplementary Figs. 4–6), although for some confounders in some instances the standardized mean difference exceeded the generally accepted threshold of 0.25 (ref. ^66^). We tested whether using a caliper of 1.5 would further improve the balance. We found that although a caliper of 1.5 significantly reduced our sample size, we nonetheless observe the same patterns as in our main results (Supplementary Text 1 and Extended Data Figs. 1c and 5).

### Post-matching analysis

Following matching, we conducted OLS regressions on the matched samples. Our post-matching OLS regressions model our outcomes measured at endline (that is, values in 2010) as a function of our treatment and our matched covariates, including outcome baseline conditions (that is, values in 2000) to control for any residual differences between treatment and control^60^. We use endline outcome values, rather than a change value between endline and baseline, for our response variable to avoid problems of spurious correlations caused by including baseline values in our response variable and as a predictor variable^69^.

Our regression model takes the form:

$${Y}_{i}=\,{\beta }_{0}\,+{{\beta }_{1}\mathrm{Tr}}_{i}+\,{{\beta }_{2}{\mathbf{X}}}_{i}+{\epsilon }_{i}$$

where *Y*_*i*_ represents the outcome variables (percentage of forest cover, income, income inequality, literacy or sanitation) in census tract *i* in 2010, *β*_0_ is the intercept, *β*_1_ is the treatment effect, Tr is a binary variable indicating the treatment (Tr = 1 for census tracts that became protected between 2000 and 2010, Tr = 0 for census tracts that remained unprotected), **X**_*i*_ is a vector of covariates we controlled for in the matching (Supplementary Table 7) and *ε*_*i*_ represents the composite error.

Our post-matching OLS regressions also included the matching weights obtained from the ‘MatchIt’ package. We used Breusch–Pagan tests using the ‘bptest’ function in the ‘lmtest’ package^70^ in R to assess heteroscedasticity in our post-matching regressions. We find substantial amounts of heteroscedasticity in our regression models (Supplementary Table 11) and therefore computed heteroscedasticity robust standard errors (HC1) using the ‘sandwich’ package^71^ in R. Given the number of models in our analysis, we also present false discovery rate^72^ adjusted *P* values, which in almost all cases show very similar levels of statistical significance as our heteroscedasticity-corrected values (Supplementary Tables 1–5).

We first compare deforestation and socio-economic indicators between protected CTs and the matched unprotected control CTs, without differentiating the latter by land cover type. We then split our control group into the different control land use types (sparsely populated areas, agricultural areas dominated by different landholding sizes and mining). We repeat the matching procedure for each control land use type to obtain an acceptable covariate balance between treatment and control CTs in each comparison.

### Reporting summary

Further information on research design is available in the Nature Portfolio Reporting Summary linked to this article.

### Supplementary information

Supplementary Information Supplementary Text 1–3, Tables 1–20, Figs. 1–6 and References.

Reporting Summary

## Data Availability

All of the data that support the findings of this study are available via Harvard Dataverse at 10.7910/DVN/AGVZAT.

## Code availability

The analysis code that supports the findings of this study is available via Harvard Dataverse at 10.7910/DVN/AGVZAT.
