# Supplementary material for: Socio-economic and environmental trade-offs in Amazonian protected areas and Indigenous territories revealed by assessing competing land uses
Source: Nat Ecol Evol. 2024 Jul 15;8(8):1482–92. doi: 10.1038/s41559-024-02458-w (PMC11310078; doi:10.1038/s41559-024-02458-w)
Supplement: Supplementary file 1 — Supplementary Text 1–3, Tables 1–20, Figs. 1–6 and References. [file 41559_2024_2458_MOESM1_ESM.pdf]

nature ecology & evolution

Supplementary information

https://doi.org/10.1038/s41559-024-02458-w

Socio-economic and environmental trade-offs in Amazonian protected areas and Indigenous territories revealed by assessing competing land uses

In the format provided by the authors and unedited

Table of Contents for Supplementary Information

Supplementary Text 2
  1. Socioeconomic development and conservation in the Brazilian Legal Amazon 2
  2. Covariates 5
  3. Robustness checks 5
Supplementary Tables 10
  Supplementary Table 1 10
  Supplementary Table 2 11
  Supplementary Table 3 12
  Supplementary Table 4 13
  Supplementary Table 5 14
  Supplementary Table 6 15
  Supplementary Table 7 16
  Supplementary Table 8 17
  Supplementary Table 9 18
  Supplementary Table 10 19
  Supplementary Table 11 20
  Supplementary Table 12 21
  Supplementary Table 13 22
  Supplementary Table 14 23
  Supplementary Table 15 24
  Supplementary Table 16. 25
  Supplementary Table 17 26
  Supplementary Table 18 27
  Supplementary Table 19 28
  Supplementary Table 20 29
Supplementary Figures 30
  Supplementary Figure 1 30
  Supplementary Figure 2 31
  Supplementary Figure 3. 32
  Supplementary Figure 4 33
  Supplementary Figure 5 34
  Supplementary Figure 6 35
References for Supplementary Information 36

Supplementary Text

1. Socioeconomic development and conservation in the Brazilian Legal Amazon

Socioeconomic development, environmental degradation, and efforts to conserve biodiversity in the Brazilian Legal Amazon (BLA) are the result of historic national development plans, market forces, and neoliberal interventions, alongside area- and incentive-based conservation policies (see ref[1] for a recent review). Protected Areas (PAs) in the BLA were initially established in remote and relatively inaccessible areas[2]; they covered approximately 44% of the region by 2010[3] (Supplementary Figure 1c). The principal aim of Strict PAs (Unidades de Proteção Integral that comprise five different categories) is environmental protection. Resource use in these areas is prohibited but they can be accessed for research, education, and in some instance tourism (e.g., National Parks)[4]. In contrast, Sustainable Use PAs (Unidades de Uso Sustentável that comprise seven different categories) aim to combine conservation with sustainable natural resource use and permit varying levels of extraction and agricultural production (e.g., Extractivist Reserves)[4]. Residence within some SPAs and SUPAs is legally permitted[4], although population densities within PA boundaries are generally low compared to adjacent areas[5]. For example, wild reserves (SPAs) allow private properties within their boundaries and National Forests and Sustainable Development Reserves (FONAs and RDS' – both SUPAs) allow traditional communities to reside within PA boundaries.

Between the 1970's and 80's, under Brazil's military government, colonization schemes (e.g., the National Integration Programme) and road construction (e.g., the Transamazonian highway) coupled with agrarian land reforms and easier access to credit and other investment incentives (e.g., tax breaks and international loans, primarily to financial elites) led to rapid deforestation. This created natural resource extraction (timber and mining, ref[6]) and agricultural frontiers. The latter were primarily cattle ranches across large land holdings but also comprised smaller more diverse agricultural production systems - although these were often bought out by larger landowners, pushing smallholders along the deforestation frontier[7].

Globalisation-driven neoliberal reforms during the 1990's and 2000's fomented large-scale infrastructure development (roads and dams) and further expansion of extractive industries (mining and oil)[1]. Mining focuses on a diverse range of resources with iron, tin, nickel, gold and copper being particularly important in official mining concessions which, whilst concentrated in eastern regions, occur across large parts of the BLA[1,8]. Illegal mining is also responsible for substantial levels

of deforestation, especially central and northern parts of the BLA[1]. Deposits of these mined resources are distributed across the BLA and there is considerable interest in gaining access to these resources and mining could occur across most of our study region[9,10].

The 1990's and 2000's also witnessed the development and consolidation of export-oriented agricultural production, predominantly by a handful of large national and international soy and beef agro-industrial companies (e.g., Bunge, Cargill and JBS). In contrast, the Brazilian Constitution of 1988 provided inalienable rights to land for Indigenous peoples. Indigenous Peoples in Brazil have and continue to face social and economic injustices, yet the 20 years between 1990 and 2010 saw the largest declaration (725,531 km^2) and homologation (903,260 km^2) of Indigenous Territories in the Legal Amazon region[11]. Indigenous Territories are found across the Amazon region, although Territories in the eastern part of the BLA form smaller contiguous areas and are less connected to PAs. Many Indigenous communities traditionally practice forms of shifting cultivation and are often autarkic, although some households and communities readily engage in commercial agriculture, the sale of forest products (including timber), and wage labour[12],

Between, 1988[1†] and 2004, deforestation in the Brazilian Legal Amazon was high with an average 18,439 km^2 of forest lost per year[13]. Deforestation decreased by approximately 75% percent between 2004 and 2012. This dramatic decrease was preceded by a number of important environmental reforms in the late 90's and early 2000's. These include: i) the establishment and implementation of sanctions and penalties for environmental infractions in 1998 (Environmental Crimes Law); the expansion of protected areas under the National System of Conservation Units in 2000; iii) a pledge to reduce greenhouse gas emissions as part of the Kyoto Protocol in 2002; and iv) the Action Plan for the Prevention and Control of Deforestation in the Legal Amazon that was implemented in 2004 by the then (and since re-elected) president, Luiz Inácio Lula da Silva. Lula's Action Plan saw an expansion of conservation units, stricter enforcement of existing anti-deforestation policies within Brazil's Forest Code (e.g., by designating some municipalities as priority areas for the prevention, monitoring and control of deforestation[14]), access to REDD+ finance mechanisms (e.g., the Bolsa Floresta programme in the state of Amazonas), and the signing

[1†] The year that the Brazilian Institute for Space Studies (INPE) launched the PRODES deforestation monitoring tool.

of the Soy Moratorium in 2006[15]. This moratorium committed traders to not trade soy grown in areas deforested after 2006. Similar moratoriums for beef and sugarcane[2‡] were signed in 2009[17].

Lula's presidency was also accompanied by the implementation of a comprehensive set of social protection initiatives under the flagship "Zero Hunger" programme. These included conditional cash transfers (Bolsa Familia) as well as low interest agricultural credit (PRONAF) for small farmers[18]. The period after 2004 saw the relative deforestation contributions of smallholders increase[19], suggesting some successes of policies aimed at large landholders (moratoriums)[15] but also the need for policies targeting different types of landholders.

Enforcement of the Forest Code on private properties has remained a challenge, even after the creation of the rural environmental cadastre system (Cadastro Ambiental Rural – CAR) designed to monitor adherence to the Forest Code[20]. Similarly, illegal logging (including in PAs and ITs) has been a substantial contributor to deforestation in the BLA and difficult to monitor and control due to corruption and fraud[21.] Enforcement of the Forest Code and other environmental legislation was substantially compromised in the run-up to and after the 2016 impeachment of Lula's successor, Dilma Rousseff. Michel Temer, who succeeded Dilma, cut and froze the Ministry of Environment's budget, and in 2018 Jair Bolsonaro was elected on an anti-environmental agenda. Deforestation, between 2012 and 2021 (after our study period), rose again by approximately 65%, including in PAs due to weakened environmental regulations[22]. Lula's re-election in October 2022 raises the potential for reversal of much of Bolsonaro's agenda and new policies that reduce deforestation and improve socioeconomic fortunes of disadvantaged groups.

In addition to socioeconomic drivers of change, the region is increasingly threatened by anthropogenic-induced biophysical and climatic changes. Deforestation-mediated seasonal changes[23] and global climate change[24] are increasing the frequency of drought events, which in turn are increasing the risk of forest fires[25] and tree mortality[26]. These long-term biophysical changes are likely to drive environmental regime shifts[27] and have substantial effects on local livelihoods and economies[24].

[2‡] Sugarcane is responsible for relatively little direct deforestation in the Brazilian Amazon but its control has been linked to the substantial expansion of sugarcane plantations in other regions of Brazil[16].

2. Covariates

We select covariates based on their potential to affect both treatment assignment (designation of protection arrangements) and our outcomes of interest (deforestation and indicators of poverty and inequality). Our selection of covariates follows other research on protected areas[28,29]. See Supplementary Tables 6 and 7 for the confounding factors used in our analysis, and the rationale for including them.

Supplementary Tables 8-10 present baseline levels of covariates for our treatment (SPAs, SUPAs, ITs) and control groups before matching. As expected, protected units show higher baseline forest cover than the unprotected controls (when defined regardless of their land use). Protected units also show lower baseline literacy levels and lower baseline income, especially for ITs. In addition, all three types of protection arrangements are established in areas with lower population density, longer travel time to major cities, and larger census tracts.

When we tease apart different land uses in our control group, before matching we observed significant differences in baseline conditions across control CTs depending on their land use. As expected, areas dominated by smallholders show higher tree cover, lower average income, and lower literacy levels at baseline than agricultural areas dominated by larger-sized landholders. However, baseline inequality before matching in these areas is also lower than in the other agricultural controls.

Prior to matching mining sites have lower forest cover at baseline compared to control non-protected CTs that are defined regardless of their land use. In contrast, baseline levels of income, literacy, and inequality are higher in control CTs with mines than non-protected CTs.

3. Robustness checks

We conducted nine robustness checks to validate our main results. For our first three robustness checks we: *i*) restricted our analysis to deforestation from 2006-2010 and PAs that were established after 2006 as 2006 is the year of the agricultural census; *ii*) raised the threshold for defining protected CTs from 10% (of the CT being protected) to 50%; and *iii*) repeated analyses using 1.5 calipers to further improve balance between treatment and control groups (we did not adopt this approach in our main analysis because it reduces sample sizes substantially – see Supplementary Table 12). In all instances, the inference from these additional analyses match those from our main analysis (Extended Data Figure 1b-d and Extended Data Figures 3-5).

For our fourth robustness check, we conduct two analyses to check for hidden bias from unmeasured confounders potentially affecting our OLS models. Potential unmeasured confounders in our analysis might include social protection programmes to curb rural poverty[18] and other interventions to halt deforestation[15,30] as well as potential political, economic and biophysical drivers of forest loss, such as election results[31], the establishment of slaughterhouses[32] and precipitation[33]. Note, that some of these factors will be associated with state, which we control for with exact matching in our main analysis.

Because unmeasured confounders can be spatially heterogeneous (e.g. precipitation and biophysical drivers of forest loss), we first test for spatial autocorrelation in our models by calculating Moran's *I* values (Supplementary Table 13). To do so, we first calculated a weight matrix based on the inverted distance between census tracts. Census tracts that were closer together, were given greater weight than census tracts further apart. We then calculated Moran's *I* index of the model residuals to detect spatial autocorrelation in our data. We used the "spdep" package in R for all spatial analyses[34]. Although spatial autocorrelation is present within our models, our estimated value of spatial autocorrelation (Moran's *I*) is often insignificant and when significant is very low (Supplementary Table 13). We thus conclude that our model inference is not biased by spatial autocorrelation and that there is negligible evidence that spatially determined unmeasured confounders influence our outcomes variables.

We also calculate Oster's regression coefficient stability estimates (also known as Oster bounds)[35] to conduct a specific hidden bias sensitivity analysis (Supplementary Table 14). We calculate Oster's regression coefficient stability estimates[35] using the "robomit" package in R. We calculate both ™ and ® values. ™ values represent the effect relative to the treatment allocation that an unmeasured confounder would need to have to flip the regression coefficient to zero. Positive and negative ™ values close to 0 represent larger relative effects and ™ values away from zero represent smaller relative effects (positive ™ values suggest a positive correlation between an unmeasured confounder and treatment selection, and negative ™ values suggest a negative correlation between unmeasured confounder and treatment selection). ™ values of 1 or -1 are considered thresholds for the presence of substantial amounts of hidden bias (i.e., the ratio of the effect of the unmeasured confounder is equal to the treatment selection). ® values represent adjusted regression coefficients when ™ = 1 or -1. Our stability estimates show that ™ > 1 or < -1 for the 41 models showing significant treatment effects (Supplementary Table 14). Similarly, ® values are comparable to our original model coefficients. Together, these results strongly indicate

that our regression models are robust to the existence of potential unmeasured confounders. Results from our final robustness check (use of a fixed effects panel analysis) further supports this conclusion (see below).

For our fifth robustness check, we consider deforestation effects of protection arrangements relative to very small landholders in greater detail by considering the amount of settled land. Results from our main analysis suggest that very small landholders have no impacts on deforestation rates contrasting with the impacts of CTs dominated by larger landholding sizes. This result could have been driven by very small landholders occupying less land (although census tracts with less than 10% agricultural land were counted as sparsely populated areas). However, in our robustness check we find that there is no significant difference in the amount of settled land occupied by very small landholders relative to small and medium landholders (Extended Data Figure 8a-c), who significantly increase deforestation rates. Similarly, we find no substantial differences in population density growth rates between different protection arrangements and alternative land uses (Supplementary Table 15), suggesting that our results are not unduly influenced by differences in population growth.

For our sixth robustness check, we consider that agriculture in the BLA is dominated by pasture, followed by cash crops (such as soy). Both types of farming are important drivers of deforestation, although their relative contributions have varied over time with pasture being more important during our focal time period[36]. Although we find that deforestation rates are similar in SPAs, SUPAs, ITs and census tracts dominated by very small agricultural land holdings, there is generally slightly less pasture in these census tracts than those dominated by larger land holdings (Extended Data Figure 8d-e). However, when we restrict analyses to CTs dominated by pasture (>70%) the results match those of our main analyses, i.e. deforestation rates remain similar across protected CTs and CTs dominated by very small agricultural land holdings, but not other landholding sizes (Extended Data Figure 1e). When we consider socioeconomic outcomes, we observe a broadly similar pattern to our main analysis (Extended Data Figure 6). Our findings thus appear to be consistent across agricultural land dominated by pasture and those dominated by other forms of agriculture.

For our seventh robustness check, we repeat our deforestation analysis using the high-resolution global deforestation maps generated by Hansen et al[37]. We do this because PRODES cumulatively measures deforestation events greater than 6.25 ha. PRODES detects loss of old growth forest and ignores secondary forest gains (e.g., those associated with shifting cultivation

systems and losses) but might fail to register small deforestation events (e.g., deforestation events linked to smallholders and small mining operations). Results using Hansen et al's[41] data, which at 30m[2] resolution has been used to measure small-scale deforestation across the Amazon[38], are equivalent to those using the PRODES dataset (Extended Data Figure 1f).

Mining effects on deforestation have been reported up to 75km from the mining site[13]. For our eight robustness check, we thus repeated analyses when changing the definition of mined CTs to any CT within 100 km, 75 km, 50 km, 25 km, 10 km and 5 km from a mine to assess the spatial extent of mining impacts. Mining associations with deforestation extended up to 75 km of a mine (Extended Data Figure 1a) while impacts on socio-economic variables extended over a much smaller distance (Extended Data Figure 2).

For our final check we tested the robustness of our results to an alternative modelling framework. We re-analysed our data using a fixed effects panel regression with two periods of data (2000 and 2010) to control for any remaining unobserved time-invariant heterogeneity between treated and control units (this is an additional alternative to testing for hidden bias). The fixed effects panel regression takes the form:

$$Y_{i,t} = \beta_0 + \beta_1 Tr_{i,t} + X_{it} + \propto_i + \gamma_t + \epsilon_{it}$$

where $Y_{i,t}$ represents outcomes (% forest cover, income, literacy, sanitation or income inequality) in census tract *i* in year *t*. Tr is a binary measure indicating whether census tract *i* is protected in year *t* (Tr = 1 for census tracts with a protected area in 2010, and 0 for all other observations). X_{it} is a matrix of time-varying covariates (outcomes other than the one of interest, population density, and, for comparisons with agricultural land use and sparsely populated areas, a binary indicator of whether a mine is present in the census tract). $\propto_i$ and γ_t represent census tract and year fixed effects respectively and ϵ_{it} represents the composite error. Observations are weighted using the matching weights employed in our main analysis. For forest outcomes the measure differs from our main analysis (which used the deforestation rate 2000-2010) as we needed data for both periods (2000 and 2010) but did not have deforestation data from before 2000. Therefore, for this analysis we used percentage forest cover in 2000 and 2010 as our measure forest change. Breusch-Pagan tests revealed that our data was heteroscedastic, so we generated heteroscedastic-robust standard errors. We ran the fixed effects panel regression separately for each outcome, protection arrangement and control land use, resulting in 105 different models. Given the large number of

models we, therefore, also generate false discovery rate[39] adjusted P values. Results from these analyses (Extended Data Figure 1g and Extended Data Figure 7) largely reflect the results from our principal analysis and/or other robustness checks.

Supplementary Tables

Supplementary Table 1: Deforestation rates (%) predicted by matched OLS regression analyses in protected CTs and unprotected controls with alternative land-uses, baseline values and regression results (two-sided) for the effect of treatment. Adjusted P-values use the false discovery rate to correct for multiple testing.

	Non-PA	Sparsely populated	Very small	Small	Medium	Large	Mining
**SPAs**							
Endline							
Treatment	3.93	2.46	2.72	2.79	3.06	2.47	3.84
Control	8.48	7.44	3.93	9.42	10.4	8.46	10.2
Difference relative to control (%)	-53.6	-66.9	-30.8	-70.4	-70.6	-70.8	-62.4
Baseline							
Treatment	0	0	0	0	0	0	0
Control	0	0	0	0	0	0	0
Regression results							
Coefficient	-4.54	-6.05	-2.93	-6.96	-9.10	-5.35	-4.50
SE	1.47	1.78	1.53	2.12	1.91	1.25	1.35
P-value	2.0E-3	7.8E-4	0.084	2.6E-3	3.1E-6	4.3E-5	1.3E-3
Adjusted P-value	8.5E-3	4.1E-4	0.19	0.011	4.1E-5	4.1E-4	6.1E-3
**SUPAs**							
Endline							
Treatment	1.94	1.45	1.51	1.47	1.45	1.47	2.02
Control	3.63	2.44	1.48	6.90	7.14	7.96	8.60
Difference relative to control (%)	-46.7	-40.5	1.66	-78.8	-79.6	-81.6	-76.5
Baseline							
Treatment	0	0	0	0	0	0	0
Control	0	0	0	0	0	0	0
Regression results							
Coefficient	-1.93	-0.76	-0.03	-6.52	-4.04	-4.94	-5.25
SE	0.30	0.38	0.29	1.59	0.49	1.76	1.53
P-value	1.8E-10	0.045	0.92	4.7E-5	1.6E-14	5.6E-3	7.4E-4
Adjusted P-value	4.6E-9	0.12	0.95	4.1E-4	1.7E-12	0.022	4.1E-3
**ITs**							
Endline							
Treatment	2.61	1.48	1.74	1.73	1.48	1.73	2.45
Control	8.40	5.44	3.32	10.02	6.42	7.65	6.59
Difference relative to control (%)	-69.0	-72.8	-47.7	-82.8	-77.0	-77.4	-62.9
Baseline							
Treatment	0	0	0	0	0	0	0
Control	0	0	0	0	0	0	0
Regression results							
Coefficient	-5.23	-3.09	-1.40	-6.73	-5.41	-6.78	-3.49
SE	0.75	0.65	0.58	1.28	0.72	2.02	1.12
P-value	4.8E-12	2.6E-6	0.016	2.5E-7	1.2E-13	8.8E-4	2.0E-3
Adjusted P-value	1.7E-10	3.9E-5	0.048	4.3E-6	6.3E-12	4.2E-3	8.5E-3

Supplementary Table 2: Income (R$), controlled for inflation, predicted by matched OLS regression analyses in protected CTs and unprotected controls with alternative land-uses, baseline values and regression results (two-sided) for the effect of treatment. Adjusted P-values use the false discovery rate to correct for multiple testing.

	Non-PA	Sparsely populated	Very small	Small	Medium	Large	Mining
**SPAs**							
Endline							
Treatment	853	804	658	709	798	783	656
Control	535	588	604	478	506	619	578
Difference relative to control (%)	59.4	36.8	8.86	48.3	57.9	26.4	13.5
Baseline							
Treatment	508	466	427	478	461	508	450
Control	527	524	650	322	458	620	591
Regression results							
Coefficient	386	131	470	219	400	261	122
SE	98.9	71.3	222	125	116	119	62.3
P-value	1.0E-6	0.066	0.060	0.089	6.1E-4	0.030	0.054
Adjusted P-value	8.5E-4	0.15	0.14	0.19	3.6E-3	0.086	0.13
**SUPAs**							
Endline							
Treatment	453	465	449	458	455	458	414
Control	417	425	441	400	439	466	459
Difference relative to control (%)	8.55	9.29	1.74	14.5	3.74	-1.78	-9.88
Baseline							
Treatment	416	411	399	405	404	405	363
Control	431	365	414	262	312	510	366
Regression results							
Coefficient	23.8	24.7	-4.12	-9.75	53.7	9.28	-101
SE	35.1	52.2	78.5	52.3	72.7	74.3	48.1
P-value	0.50	0.64	0.96	0.85	0.46	0.90	0.037
Adjusted P value	0.64	0.73	0.97	0.91	0.59	0.93	0.96
**ITs**							
Endline							
Treatment	373	337	323	324	337	324	355
Control	497	497	503	389	384	460	484
Difference relative to control (%)	-24.8	-32.2	-35.8	-16.7	-12.3	-29.6	-26.5
Baseline							
Treatment	353	310	290	290	310	290	342
Control	397	366	472	256	293	472	506
Regression results							
Coefficient	-101	-82.9	-98.4	-32.1	-8.38	-149	-22.6
SE	27.2	35.8	29.3	39.8	28.9	55.5	46.6
P-value	2.0E-4	0.021	8.6E-4	0.42	0.77	7.7E-3	0.63
Adjusted P-value	1.5E-3	0.063	4.2E-3	0.56	0.86	0.027	0.72

Supplementary Table 3: Income inequality (GINI index) predicted by matched OLS regression analyses in protected CTs and unprotected controls with alternative land-uses, baseline values and regression results (two-sided) for the effect of treatment. Adjusted P-values use the false discovery rate to correct for multiple testing.

	Non-PA	Sparsely populated	Very small	Small	Medium	Large	Mining
**SPAs**							
Endline							
Treatment	0.42	0.42	0.43	0.42	0.42	0.40	0.40
Control	0.40	0.39	0.41	0.45	0.38	0.39	0.36
Difference relative to control (%)	3.17	8.17	3.39	-7.04	9.95	2.27	13.71
Baseline							
Treatment	0.35	0.33	0.33	0.33	0.33	0.35	0.32
Control	0.39	0.37	0.38	0.29	0.38	0.43	0.37
Regression results							
Coefficient	0.04	0.02	-0.14	-0.06	0.03	0.03	0.06
SE	0.03	0.03	0.10	0.05	0.04	0.05	0.03
P-value	0.18	0.51	0.21	0.18	0.45	0.56	0.060
Adjusted P-value	0.34	0.62	0.34	0.31	0.59	0.67	0.14
**SUPAs**							
Endline							
Treatment	0.40	0.39	0.38	0.39	0.39	0.39	0.39
Control	0.39	0.39	0.43	0.44	0.24	0.43	0.36
Difference (%)	1.70	1.39	-11.35	-12.3	59.1	-9.01	10.6
Baseline							
Treatment	0.37	0.36	0.36	0.36	0.36	0.36	0.36
Control	0.39	0.38	0.41	0.33	0.34	0.48	0.35
Regression results							
Coefficient	0.01	0.02	-0.03	-0.12	0.13	-0.02	0.04
SE	0.01	0.01	0.02	0.02	0.03	0.02	0.02
P-value	0.29	0.28	0.10	1.7E-7	9.3E-6	0.40	0.12
Adjusted P-value	0.43	0.42	0.20	3.5E-6	1.1E-4	0.56	0.25
**ITs**							
Endline							
Treatment	0.37	0.36	0.36	0.36	0.36	0.36	0.37
Control	0.39	0.39	0.45	0.42	0.36	0.45	0.34
Difference (%)	-3.78	-8.17	-21.4	-14.3	-2.05	-20.1	-9.13
Baseline							
Treatment	0.36	0.35	0.32	0.32	0.35	0.32	0.35
Control	0.33	0.35	0.41	0.30	0.38	0.50	0.43
Regression results							
Coefficient	-0.02	-0.03	-0.11	-0.08	-0.03	-0.02	0.09
SE	0.01	0.02	0.03	0.02	0.02	0.03	0.03
P-value	0.18	0.12	3.5E-5	3.6E-4	0.18	0.47	8.9E-3
Adjusted P-value	0.31	0.24	3.7E-4	2.3E-3	0.31	0.60	0.031

Supplementary Table 4: Proportion of households with poor sanitation predicted by matched OLS regression analyses in protected CTs and unprotected controls with alternative land-uses, baseline values and regression results (two-sided) for the effect of treatment. Adjusted P-values use the false discovery rate to correct for multiple testing.

	Non-PA	Sparsely populated	Very small	Small	Medium	Large	Mining
**SPAs**							
Endline							
Treatment	0.95	0.95	0.96	0.96	0.95	0.95	0.95
Control	0.94	0.92	1.00	0.98	0.97	0.79	0.97
Difference relative to control (%)	0.64	3.34	-4.04	-1.81	-1.52	20.9	-2.34
Baseline							
Treatment	0.96	0.96	0.96	0.96	0.96	0.96	0.98
Control	0.93	0.82	0.91	0.99	0.88	0.80	0.98
Regression results							
Coefficient	-0.02	0.08	0.05	-0.01	-0.01	0.17	-0.02
SE	0.02	0.04	0.04	0.02	0.02	0.08	0.02
P-value	0.21	0.031	0.23	0.55	0.50	0.028	0.13
Adjusted P-value	0.34	0.086	0.35	0.66	0.62	0.082	0.26
**SUPAs**							
Endline							
Treatment	0.95	0.95	0.96	0.96	0.96	0.96	0.96
Control	0.94	0.95	0.98	0.96	0.96	0.96	0.96
Difference relative to control (%)	0.99	0.58	-2.71	-0.14	-0.57	-0.08	-0.19
Baseline							
Treatment	0.96	0.97	0.97	0.97	0.97	0.97	0.97
Control	0.94	0.96	0.96	0.98	0.97	0.95	0.93
Regression results							
Coefficient	0.00	0.00	-0.03	-0.01	0.00	0.02	-0.01
SE	0.01	0.01	0.01	0.01	0.01	0.02	0.01
P-value	0.60	0.73	5.6E-4	0.46	0.97	0.32	0.44
Adjusted P-value	0.71	0.82	3.4E-3	0.59	0.97	0.48	0.59
**ITs**							
Endline							
Treatment	0.95	0.95	0.96	0.96	0.95	0.96	0.95
Control	0.92	0.93	0.94	0.95	0.89	0.96	0.90
Difference relative to control (%)	2.37	2.62	1.53	0.77	6.45	-0.72	5.11
Baseline							
Treatment	0.97	0.97	0.99	0.99	0.97	0.99	0.98
Control	0.98	0.95	0.98	1.00	0.98	1.00	0.92
Regression results							
Coefficient	0.01	0.01	-0.04	0.00	0.03	-0.04	-0.01
SE	0.01	0.01	0.02	0.02	0.02	0.03	0.02
P-value	0.15	0.37	0.053	0.82	0.11	0.17	0.84
Adjusted P-value	0.029	0.53	0.13	0.90	0.22	0.30	0.91

Supplementary Table 5: Literacy rates predicted by matched OLS regression analyses in protected CTs and unprotected controls with alternative land-uses, baseline values and regression results (two-sided) for the effect of treatment. Adjusted P-values use the false discovery rate to correct for multiple testing.

	Non-PA	Sparsely populated	Very small	Small	Medium	Large	Mining
**SPAs**							
Endline							
Treatment	0.72	0.69	0.67	0.68	0.69	0.69	0.70
Control	0.73	0.74	0.69	0.73	0.76	0.75	0.78
Difference relative to control (%)	-1.97	-7.59	-3.71	-5.95	-9.28	-8.20	-9.54
Baseline							
Treatment	0.60	0.58	0.52	0.58	0.58	0.60	0.58
Control	0.65	0.57	0.64	0.53	0.66	0.65	0.69
Regression results							
Coefficient	0.00	0.07	0.10	0.04	0.04	0.01	0.02
SE	0.02	0.03	0.06	0.04	0.03	0.03	0.02
P-value	0.86	0.034	0.15	0.34	0.22	0.80	0.35
Adjusted P-value	0.92	0.091	0.28	0.49	0.35	0.88	0.50
**SUPAs**							
Endline							
Treatment	0.66	0.66	0.65	0.65	0.65	0.65	0.64
Control	0.63	0.60	0.64	0.71	0.76	0.76	0.67
Difference relative to control (%)	5.67	9.18	1.98	-8.49	-13.9	-13.5	-3.09
Baseline							
Treatment	0.55	0.55	0.54	0.54	0.54	0.54	0.54
Control	0.54	0.49	0.60	0.43	0.62	0.67	0.53
Regression results							
Coefficient	0.03	0.03	0.05	0.04	0.04	0.01	0.02
SE	0.01	0.02	0.02	0.02	0.02	0.02	0.01
P-value	0.011	0.09	0.012	0.013	0.0063	0.63	0.16
Adjusted P-value	0.038	0.19	0.039	0.041	0.024	0.72	0.29
**ITs**							
Endline							
Treatment	0.60	0.57	0.59	0.59	0.57	0.59	0.59
Control	0.69	0.67	0.65	0.72	0.64	0.76	0.71
Difference relative to control (%)	-12.8	-14.6	-10.2	-18.1	-10.7	-22.1	-17.00
Baseline							
Treatment	0.47	0.43	0.39	0.39	0.43	0.39	0.46
Control	0.54	0.53	0.58	0.35	0.53	0.67	0.61
Regression results							
Coefficient	-0.02	0.00	0.03	-0.11	0.02	-0.01	0.04
SE	0.02	0.02	0.02	0.03	0.03	0.04	0.03
P-value	0.40	0.94	0.21	2.4E-4	0.41	0.90	0.17
Adjusted P-value	0.55	0.97	0.34	1.6E-3	0.56	0.94	0.31

Supplementary Table 6. Description of covariates used in analyses.

Covariate	Definition and data source	Rationale for inclusion
Baseline forest cover	PRODES[13]	Forests have a direct, by providing valuable forest products, and indirect effect (e.g. by providing ecosystem services) on socioeconomic status of households[40]. Note, in all cases and following standard practice, we match on baseline values of our outcome variable
Baseline income	Average income of respondents in the census tract in 2000 in Reais per month (IBGE)[41]	Socioeconomic status plays an important role in deforestation decisions[40] and we match on other socio-economic indicators when modelling socio-economic outcomes to capture their interactions e.g. literacy can influence income.
Baseline literacy	Average literacy rate of respondents in the census tract in 2000 (IBGE)[41]	
Baseline sanitation	Access to improved sanitation (see MDG guidelines in ref[42]) of respondents in the census tract in 2000 (IBGE)[41]. Categories "piped sewer system" (*rede geral de esgoto ou pluvial*) and "septic tanks" (*fossa séptica*) were considered improved. Categories "pit latrine" (*fossa rudimentar*), "open pit" (*vala*), "river, lake or sea" (*rio, lago o mar*), and "other" (*outro escoadouro*) were considered unimproved.	
Baseline GINI index	GINI index, calculated from income levels of respondents in the census tract in 2000 (IBGE)[41]	
Area	Size of the census tract (km^2). Data from IBGE[43]	Size of the administrative area is linked to deforestation rates and socioeconomic outcomes[28]
Population density	Population density year 2000 (IBGE)[41]	Population pressure affects socioeconomic outcomes, deforestation and establishment of protection arrangements[44]
Proportion settled area	Proportion of settled land in the census tract, based on calculations from agricultural census (IBGE)[45].	Land settlement influences land use choices, socioeconomic status, and deforestation[46]
Mining	Binary exact match based on the presence/absence of legal mining concessions (SIGMINE)[47].	Mining influences land use choices, socioeconomic and deforestation outcomes, and establishment of protection arrangements[48,49]
Travel time to a population centre (>50,000 inhabitants)	Travel time to major cities using calculations from Nolte et al.[50], based on ref[51]	Access to markets and services (e.g., technical assistance, health centres) significantly affects livelihoods, decisions about establishing protection arrangements, and other related land-use choices[52].
Slope (degrees)	International Institute for Applied Systems Analysis[53]	Slope and elevation are important factors in determining agricultural suitability, and also affect where PAs are located as PAs are generally located at higher elevation and on steeper slopes[2]. Slope and elevation could also affect the development of mines[54].
Elevation (meters)		
Flood risk (percentage)	Percentage of census tract that is seasonally flooded, according to the ESA land cover map[55]	Flood risk is an important factor in determining agricultural suitability and mining[54].
State	Exact match based on the state the CT is located in.	Federal states have significant autonomy in decision-making and planning that can influence deforestation and development.

Supplementary Table 7. Covariates used in different model specifications comparing protection arrangements (SPAs, SUPAs and ITs) to alternative land uses. When comparing protected CTs to CTs with mining, we excluded CTs with mines that were established before 2000 and excluded protected CTs with mines established after 2000. See methods text in the main manuscript for rationales about the use of proportion settled area and mining as covariates in specific analyses.

	Protection comparison to:		
**Covariate**	**Non-PA**	**Agricultural landholding sizes and sparsely populated areas**	**Mining concessions**
Baseline forest cover	•	•	•
Baseline income	•	•	•
Baseline literacy	•	•	•
Baseline sanitation	•	•	•
Baseline GINI index	•	•	•
Area	•	•	•
Population density	•	•	•
Travel time to pop. centre (>50,000 inhabitants)	•	•	•
Slope (degrees)	•	•	•
Elevation (meters)	•	•	•
Flood risk (percentage)	•	•	•
Proportion settled area			•
Mines established before 2000	•	•	
Mines established after 2000		•	
State	•	•	•

Supplementary Table 8. **Mean values and standard errors for confounders, prior to matching, in census tracts protected with Strict Protected Areas (SPAs) and control census tracts.** Note that proportion settled land was only included as a covariate in analysis comparing SPAs to mining.

	SPAs est. after 2000	Non-PA	Sparsely populated (<10% of land settled)	Very small (<10 ha)	Small (10-50 ha)	Medium (50-200 ha)	Large (>200 ha)	Mining
Baseline forest cover (%)	0.78 ± 0.03	0.37 ± 0.01	0.58 ± 0.01	0.31 ± 0.01	0.29 ± 0.01	0.36 ± 0.01	0.39 ± 0.01	0.33 ± 0.01
Travel time to major cities (minutes)	4473 ± 340	1421 ± 27	2113 ± 94	1060 ± 60	1126 ± 30	1408 ± 48	1595 ± 67	1353 ± 81
Baseline population density (pop. per km^2)	0.54 ± 0.20	13.58 ± 2.34	30.65 ± 12.99	20.99 ± 2.26	9.05 ± 0.87	6.30 ± 0.90	10.63 ± 5.56	24.12 ± 7.76
Area of census tract (km^2)	4175 ± 557	469 ± 17	806 ± 74	180 ± 15	329 ± 15	379 ± 17	861 ± 56	575 ± 57
Baseline income (Reais)	538.77 ± 45.69	651.46 ± 13.69	427.13 ± 14.61	540.77 ± 25.29	607.25 ± 16.98	678.04 ± 18.51	1125.51 ± 82.04	859.15 ± 77.67
Baseline literacy (%)	0.62 ± 0.03	0.66 ± 0.00	0.58 ± 0.01	0.62 ± 0.01	0.66 ± 0.01	0.68 ± 0.01	0.75 ± 0.01	0.71 ± 0.01
Baseline GINI index	0.36 ± 0.02	0.43 ± 0.00	0.38 ± 0.01	0.43 ± 0.01	0.44 ± 0.00	0.45 ± 0.00	0.45 ± 0.01	0.46 ± 0.01
Baseline good sanitation (%)	0.11 ± 0.02	0.14 ± 0.00	0.19 ± 0.01	0.20 ± 0.02	0.10 ± 0.01	0.12 ± 0.01	0.13 ± 0.01	0.15 ± 0.02
Slope (degree)	2.87 ± 0.17	3.29 ± 0.10	2.51 ± 0.10	2.84 ± 0.10	3.64 ± 0.08	3.82 ± 0.08	2.84 ± 0.11	3.22 ± 0.10
Elevation (m)	179.95 ± 14.14	175.66 ± 2.59	82.33 ± 4.35	137.09 ± 7.90	191.00 ± 84.28	192.60 ± 3.97	266.53 ± 7.69	215.38 ± 9.14
Flood risk (%)	0.78 ± 0.03	0.37 ± 0.01	0.58 ± 0.01	0.31 ± 0.01	0.29 ± 0.01	0.36 ± 0.01	0.39 ± 0.01	0.33 ± 0.01
Proportion settled land	0.04 ± 0.02	NA	NA	NA	NA	NA	NA	0.48 ± 0.03
**Sample size**	**58**	**2000**	**336**	**250**	**602**	**569**	**243**	**174**

Supplementary Table 9. Mean and standard errors for confounders, prior to matching, in census tracts protected with Sustainable Use Protected Areas (SUPAs) and control census tracts. Note that proportion settled land was only included as a covariate in analysis comparing SUPAs to mining.

	SUPAs est. after 2000	Non-PA	Sparsely populated (<10% of land settled)	Very small (<10 ha)	Small (10-50 ha)	Medium (50-200 ha)	Large (>200 ha)	Mining
Baseline forest cover (%)	0.73 ± 0.02	0.38 ± 0.01	0.59 ± 0.01	0.32 ± 0.01	0.29 ± 0.01	0.36 ± 0.01	0.39 ± 0.01	0.34 ± 0.01
Travel time to major cities (minutes)	3695 ± 208	1489 ± 28	2338 ± 99	1086 ± 61	1138 ± 30	1429 ± 48	1601 ± 64	1380 ± 79
Baseline population density (pop. per km2)	7.70 ± 2.96	13.07 ± 2.22	26.94 ± 11.34	20.79 ± 2.20	9.06 ± 0.86	6.15 ± 0.87	10.09 ± 5.25	23.35 ± 11.34
Area of census tract (km2)	2086 ± 309	510 ± 18	894 ± 70	179 ± 15	337 ± 16	402 ± 18	925 ± 62	610 ± 60
Baseline income (Reais)	416.19 ± 33.79	641.63 ± 13.13	413.35 ± 13.25	536.87 ± 24.78	608.62 ± 16.81	670.68 ± 18.03	1103.83 ± 77.87	844.95 ± 75.35
Baseline literacy (%)	0.55 ± 0.01	0.66 ± 0.00	0.57 ± 0.01	0.62 ± 0.01	0.66 ± 0.01	0.67 ± 0.01	0.75 ± 0.01	0.71 ± 0.01
Baseline GINI index	0.37 ± 0.01	0.43 ± 0.00	0.38 ± 0.01	0.42 ± 0.01	0.43 ± 0.00	0.45 ± 0.01	0.44 ± 0.01	0.46 ± 0.01
Baseline good sanitation (%)	0.17 ± 0.02	0.14 ± 0.00	0.19 ± 0.01	0.20 ± 0.02	0.10 ± 0.01	0.12 ± 0.01	0.13 ± 0.01	0.15 ± 0.02
Slope (degree)	2.87 ± 0.17	3.29 ± 0.10	2.51 ± 0.10	2.84 ± 0.10	3.64 ± 0.08	3.82 ± 0.08	2.84 ± 0.11	3.22 ± 0.10
Elevation (m)	89.48 ± 5.69	173.98 ± 2.52	83.19 ± 3.94	134.59 ± 7.78	192.00 ± 4.24	192.20 ± 3.90	264.37 ± 7.48	215.50 ± 9.08
Flood risk (%)	0.12 ± 0.02	0.06 ± 0.00	0.12 ± 0.01	0.09 ± 0.01	0.04 ± 0.00	0.02 ± 0.00	0.04 ± 0.01	0.03 ± 0.00
Proportion settled land	0.08 ± 0.01	NA	NA	NA	NA	NA	NA	0.43 ± 0.03
**Sample size**	**158**	**2102**	**385**	**256**	**616**	**588**	**257**	**180**

Supplementary Table 10. Mean difference and standard errors for confounders, prior to matching, in census tracts protected with Indigenous Territories (ITs) and control census tracts. Note that proportion settled land was only included as a covariate in analysis comparing ITs to mining.

	**ITs est. after 2000**	**Non-PA**	**Sparsely populated (<10% of land settled)**	**Very small (<10 ha)**	**Small (10-50 ha)**	**Medium (50-200 ha)**	**Large (>200 ha)**	**Mining**
Baseline forest cover (%)	0.74 ± 0.02	0.38 ± 0.01	0.59 ± 0.01	0.32 ± 0.01	0.29 ± 0.01	0.36 ± 0.01	0.39 ± 0.01	0.34 ± 0.01
Travel time to major cities (minutes)	5256 ± 239	1489 ± 28	2338 ± 99	1086 ± 61	1138 ± 30	1429 ± 48	1601 ± 64	1380 ± 79
Baseline population density (pop. per km^2)	1.93 ± 0.53	13.07 ± 2.22	26.94 ± 11.34	20.79 ± 2.21	9.06 ± 0.86	6.15 ± 0.87	10.09 ± 5.26	23.35 ± 7.51
Area of census tract (km^2)	2041 ± 174	510 ± 18	894 ± 70	179 ± 15	337 ± 16	402 ± 18	925 ± 62	610 ± 60
Baseline income (Reais)	353.45 ± 21.03	641.63 ± 13.13	413.35 ± 13.25	536.87 ± 24.78	608.62 ± 16.81	670.68 ± 18.03	1103.83 ± 77.87	844.95 ± 75.35
Baseline literacy (%)	0.47 ± 0.02	0.66 ± 0	0.57 ± 0.01	0.62 ± 0.01	0.66 ± 0.01	0.67 ± 0.01	0.75 ± 0.01	0.71 ± 0.01
Baseline GINI index	0.36 ± 0.01	0.43 ± 0.00	0.38 ± 0.01	0.42 ± 0.01	0.43 ± 0.00	0.45 ± 0.00	0.44 ± 0.01	0.46 ± 0.01
Baseline good sanitation (%)	0.10 ± 0.01	0.14 ± 0.00	0.19 ± 0.01	0.20 ± 0.02	0.10 ± 0.01	0.12 ± 0.01	0.13 ± 0.01	0.15 ± 0.02
Slope (degree)	4.12 ± 0.22	3.23 ± 0.07	3.72 ± 0.10	2.84 ± 0.10	3.64 ± 0.08	3.82 ± 0.08	2.84 ± 0.11	3.22 ± 0.13
Elevation (m)	220.99 ± 12.2	173.98 ± 2.52	83.19 ± 3.94	134.59 ± 7.78	192.00 ± 4.24	192.20 ± 3.90	264.37 ± 7.48	215.50 ± 9.09
Flood risk (%)	0.74 ± 0.02	0.38 ± 0.01	0.59 ± 0.01	0.32 ± 0.01	0.29 ± 0.01	0.36 ± 0.01	0.39 ± 0.01	0.34 ± 0.01
Proportion settled land	0.05 ± 0.01	NA	NA	NA	NA	NA	NA	0.46 ± 0.03
**Sample size**	**202**	**2102**	**385**	**256**	**616**	**588**	**257**	**180**

Supplementary Table 11. Breusch-Pagan tests (one-sided) for heteroscedasticity

PA type	Control	Deforestation		Average Monthly Income 2010 (Reais)		Literacy rate 2010		Percentage with poor sanitation 2010		GINI index 2010	
		BP	P-value	BP	P-value	BP	P-value	BP	P-value	BP	P-value
SPA	Non-PA	143	<0.001	33.0	0.017	391	<0.001	138	<0.001	126	<0.001
	Sparsely populated	132	<0.001	33.9	0.013	54.1	<0.001	25.9	0.101	38.4	0.003
	Very small	12.3	0.582	15.5	0.347	15.8	0.328	12.6	0.560	13.8	0.467
	Small	5.6	0.995	20.6	0.246	17.8	0.401	1.15	1.000	16.1	0.520
	Medium	11.9	0.889	23.9	0.199	47.8	<0.001	8.19	0.985	31.7	0.034
	Large	2.5	1.000	13.9	0.677	10.5	0.880	3.39	1.000	16.2	0.511
	Mining	6.1	0.986	7.33	0.966	23.6	0.100	1.54	1.000	6.52	0.981
SUPA	Non-PA	29.9	0.038	36.1	0.007	176	<0.001	7.68	0.983	48.3	<0.001
	Sparsely populated	93.1	<0.001	26.3	0.093	37.7	0.004	16.4	0.566	22.4	0.215
	Very small	5.3	0.994	32.6	0.008	25.4	0.063	2.50	1.000	19.3	0.252
	Small	19.2	0.319	64.0	<0.001	43.7	<0.001	2.08	1.000	24.5	0.105
	Medium	10.1	0.967	31.8	0.045	13.5	0.853	7.00	0.997	11.8	0.922
	Large	7.0	0.990	35.5	0.008	7.95	0.979	4.26	1.000	5.11	0.999
	Mining	21.0	0.225	33.9	0.009	14.4	0.641	3.32	1.000	6.5	0.983
IT	Non-PA	218	<0.001	36.0	0.010	194	<0.001	44.7	0.001	153	<0.001
	Sparsely populated	83.8	<0.001	46.6	<0.001	60.5	<0.001	100	0.000	71.7	<0.001
	Very small	31.3	0.026	18.0	0.453	52.3	<0.001	19.4	0.369	67.9	<0.001
	Small	30.5	0.033	73.8	0.000	61.3	<0.001	6.79	0.992	57.3	<0.001
	Medium	26.4	0.118	32.6	0.026	168	<0.001	13.1	0.831	80.1	<0.001
	Large	26.9	0.082	7.3	0.987	79.5	<0.001	2.58	1.000	36.8	0.006
	Mining	17.2	0.574	3.7	1.000	20.2	0.384	89.2	<0.001	56.8	<0.001

Supplementary Table 12. Number of treatment (T) and control (C) census tracts used in analyses with and without calipers when comparing effects of Strict Protected Areas (SPAs), Sustainable Use Protected Areas (SUPAs) and Indigenous Territories (ITs) relative to alternative land uses.

		Non-PA		Sparsely settled (<10% of land settled)		Very small (<10 ha)		Small (10-50 ha)		Medium (50-200 ha)		Large (>200 ha)		Mining	
		T	C	T	C	T	C	T	C	T	C	T	C	T	C
SPA	No caliper	48	1510	40	218	22	3	34	13	41	265	44	66	33	55
	1.5 caliper	39	844	34	175			19	10	29	162	12	6	33	40
SUPA	No caliper	158	1077	123	349	111	109	117	381	118	120	117	82	134	50
	1.5 caliper	151	929	117	326	94	91	95	205	109	136	72	42	118	40
IT	No caliper	202	1948	172	359	131	194	132	325	172	537	132	211	188	162
	1.5 caliper	176	1807	155	331	105	109	102	369	136	348	70	177	155	144

Supplementary Table 13. Spatial autocorrelation tests (two-sided) for different outcomes for Strict Protected Areas (SPAs), Sustainable Use Protected Areas (SUPAs), Indigenous Territories (ITs) and their respective control census tracts.

PA type	Control	Deforestation		Average Monthly Income 2010 (Reais)		Literacy rate 2010		Percentage with poor sanitation 2010		GINI index 2010	
		Moran's *I*	P-value	Moran's *I*	P-value	Moran's *I*	P-value	Moran's *I*	P-value	Moran's *I*	P-value
SPA	Non-PA	0.088	0.001	0.011	0.632	0.013	0.573	-0.037	0.212	0.057	0.032
	Sparsely populated	-0.177	0.598	0.224	0.112	-0.171	0.588	-0.072	0.421	0.063	0.245
	Very small	-0.022	0.962	-0.299	0.242	0.030	0.868	-0.128	0.635	-0.096	0.729
	Small	0.141	0.016	0.008	0.763	-0.022	0.864	-0.012	0.991	-0.016	0.936
	Medium	0.084	0.341	-0.006	0.821	-0.259	0.069	0.112	0.237	-0.049	0.909
	Large	0.085	0.160	-0.162	0.038	0.122	0.053	0.053	0.341	-0.046	0.654
	Mining	-0.030	0.980	0.028	0.724	0.047	0.630	0.054	0.601	0.187	0.162
SUPA	Non-PA	0.067	0.020	0.059	0.038	0.011	0.640	0.014	0.562	0.061	0.034
	Sparsely populated	0.040	0.495	0.055	0.380	-0.022	0.911	0.024	0.634	0.029	0.585
	Very small	0.082	0.093	0.062	0.194	0.062	0.197	-0.023	0.757	0.056	0.239
	Small	-0.002	0.887	0.038	0.535	-0.005	0.918	-0.022	0.920	0.003	0.847
	Medium	0.012	0.792	-0.063	0.568	0.092	0.253	-0.095	0.355	0.144	0.046
	Large	0.068	0.133	0.020	0.588	0.002	0.855	0.002	0.862	0.000	0.893
	Mining	0.092	0.230	0.009	0.773	-0.038	0.820	-0.086	0.449	-0.039	0.808
IT	Non-PA	0.072	0.001	-0.011	0.688	0.021	0.300	-0.014	0.597	0.042	0.049
	Sparsely populated	-0.007	0.971	0.006	0.816	0.076	0.172	-0.021	0.849	0.043	0.409
	Very small	0.157	0.003	-0.059	0.356	0.028	0.514	-0.086	0.157	0.062	0.221
	Small	0.064	0.092	0.021	0.519	0.094	0.016	0.072	0.061	0.052	0.161
	Medium	0.041	0.463	-0.024	0.809	0.115	0.065	-0.054	0.486	0.052	0.363
	Large	0.046	0.285	-0.027	0.700	0.057	0.200	0.002	0.853	0.063	0.161
	Mining	0.216	0.001	0.007	0.787	0.006	0.804	-0.008	0.968	0.119	0.057

Supplementary Table 14. Oster δ and β values for post-matching regression models

PA type	Control	Deforestation		Average Monthly Income 2010 (Reais)		Literacy rate 2010		Percentage with poor sanitation 2010		GINI index 2010	
		δ	β (δ = 1 | -1)	δ	β (δ = 1 | -1)	δ	β (δ = 1 | -1)	δ	β (δ = 1 | -1)	δ	β (δ = 1 | -1)
SPA	Non-PA	30.2	-4.54	-28.2	363	-0.65	-0.002	-2.43	-0.011	-5.28	0.028
	Sparsely populated	57.2	-6.52	2.90	95.1	62.6	-0.073	-4.64	0.061	3.62	0.017
	Very small	29.8	-5.49	-4.10	244	-10.8	-0.058	-0.94	-0.003	-1.99	-0.054
	Small	3.60	-7.25	4.61	213	4.54	-0.037	3.48	-0.012	-10.5	-0.051
	Medium	5.87	-10.4	6.45	492	1.38	-0.013	3.68	-0.012	3.36	0.035
	Large	1.35	-3.73	7.90	358	0.21	0.042	2.36	0.172	-1.84	-0.014
	Mining	2.19	-3.20	-61.4	145	-0.53	-0.019	6.97	-0.024	12.5	0.067
SUPA	Non-PA	-36.5	-1.86	5.86	20.0	10.8	0.026	3.06	0.003	-8.60	0.010
	Sparsely populated	6.18	-0.67	3.79	19.2	2.34	0.016	-1.25	-0.001	-5.18	0.012
	Very small	-1.47	-0.01	-0.92	0.34	-4.79	0.034	10.9	-0.031	2.38	-0.025
	Small	26.8	-7.04	-0.38	14.7	3.73	-0.037	-4.08	-0.006	-2.28	-0.094
	Medium	1.64	-2.26	-5.52	39.6	1.29	-0.012	-0.18	-0.002	2.14	0.123
	Large	1.05	-0.64	-1.10	0.63	0.17	0.078	-2.98	0.008	0.77	0.009
	Mining	2.48	-4.29	-6.89	-125	11.0	-0.020	-4.59	-0.006	6.45	0.034
IT	Non-PA	9.69	-5.01	7.51	-93.2	0.70	0.008	4.43	0.012	-35.7	-0.017
	Sparsely populated	3.46	-2.63	1.85	-44.2	-0.05	-0.037	1.71	0.005	5.87	-0.028
	Very small	2.48	-1.23	1.05	-7.04	-0.54	-0.020	-1.65	-0.012	1.33	-0.148
	Small	2.32	-5.48	1.34	-10.4	2.54	-0.102	2.00	0.003	8.81	-0.096
	Medium	10.4	-5.67	0.51	9.0	-0.65	-0.012	2.56	0.023	-4.64	-0.024
	Large	2.06	-8.26	3.02	-163	0.04	0.121	-4.65	-0.025	0.36	0.052
	Mining	12.9	-3.26	0.67	10.8	-0.81	16.0	-0.33	-5.138	-1.23	5.86

Supplementary Table 15. Population density growth rates in Strict Protected Areas (SPAs), Sustainable Use Protected Areas (SUPAs) and Indigenous Territories (ITs) and their respective control census tracts. P-values are based on two-sided t-tests.

PA type	Control	Pop. density growth rate in protected CTs Mean (SE)	Pop. density growth rate in control CTs Mean (SE)	P-value
SPA	Non-PA	3.89 (1.66)	2.03 (1.10)	0.27
	Sparsely populated	3.32 (2.03)	0.19 (0.21)	0.13
	Very small	3.32 (2.01)	2.52 (1.54)	0.70
	Small	3.53 (1.70)	0.48 (0.31)	0.08
	Medium	3.32 (2.01)	0.83 (0.66)	0.22
	Large	3.53 (1.70)	0.88 (0.62)	0.12
	Mining	2.64 (1.06)	0.45 (0.19)	0.04
SUPA	Non-PA	0.84 (0.44)	0.35 (0.06)	0.27
	Sparsely populated	0.45 (0.34)	0.16 (0.20)	0.41
	Very small	0.49 (0.32)	1.52 (1.01)	0.003
	Small	0.49 (0.32)	0.42 (0.34)	0.84
	Medium	0.49 (0.32)	0.85 (0.55)	0.31
	Large	0.54 (0.31)	0.71 (0.24)	0.59
	Mining	0.89 (0.52)	0.52 (0.27)	0.49
IT	Non-PA	3.89 (1.66)	2.03 (1.10)	0.27
	Sparsely populated	3.33 (2.03)	0.19 (0.21)	0.13
	Very small	3.32 (2.01)	2.52 (1.54)	0.70
	Small	3.53 (1.70)	0.48 (0.31)	0.075
	Medium	3.32 (2.01)	0.83 (0.66)	0.22
	Large	3.53 (1.70)	0.88 (0.62)	0.12
	Mining	2.64 (1.06)	0.45 (0.19)	0.042

Supplementary Table 16. Regression results (two-sided) of robustness tests for the effect of treatment on deforestation. Adjusted P-values use the false discovery rate to correct for multiple testing. Please note that we only show adjusted P-values for the fixed effects panel model to mimic our main analysis.

Robustness test (Figure)	PA type	Control						
		Main analysis	5 km buffer	10 km buffer	25 km buffer	50 km buffer	75 km buffer	100 km buffer
Mining buffers (Ext. Data Fig. 2a)	SPA	Coef. = -4.68<br>SE = 1.44<br>P = 1.8E-3	Coef. = -5.39<br>SE = 1.10<br>P = 3.3E-6	Coef. = -3.82<br>SE = 1.13<br>P = 1.0E-3	Coef. = -5.05<br>SE = 0.97<br>P = 4.9E-7	NA	NA	NA
	SUPA	Coef. = -5.26<br>SE = 1.48<br>P = 4.7E-4	Coef. = -4.69<br>SE = 0.95<br>P = 1.6E-6	Coef. = -2.17<br>SE = 0.42<br>P = 7.2E-7	Coef. = -1.74<br>SE = 0.59<br>P = 3.6E-3	Coef. = -2.22<br>SE = 1.25<br>P = 0.080	Coef. = 0.17<br>SE = 0.43<br>P = 0.68	Coef. = -0.03<br>SE = 0.51<br>P = 0.95
	IT	Coef. = -3.47<br>SE = 1.13<br>P = 2.4E-3	Coef. = -2.43<br>SE = 0.57<br>P = 2.3E-5	Coef. = -2.27<br>SE = 0.73<br>P = 2.0E-3	Coef. = -3.46<br>SE = 0.83<br>P = 3.9E-5	Coef. = -1.09<br>SE = 0.49<br>P = 0.025	Coef. = -0.93<br>SE = 0.24<br>P = 1.4E-4	Coef. = -0.41<br>SE = 0.24<br>P = 0.094
		**Non-PA**	**Sparsely Populated**	**Very small**	**Small**	**Medium**	**Large**	**Mining**
Est. after 2006 (Ext. Data Fig. 2b)	SPA	NA	Coef. = -4.63<br>SE = 1.73<br>P = 7.8E-3	Coef. = -1.88<br>SE = 3.45<br>P = 0.61	Coef. = -6.94<br>SE = 2.08<br>P = 2.9E-3	Coef. = -14.11<br>SE = 2.06<br>P = 5.8E-11	Coef. = -2.83<br>SE = 3.33<br>P = 0.41	NA
	SUPA	NA	Coef. = -1.81<br>SE = 0.49<br>P = 2.4E-4	Coef. = -0.68<br>SE = 0.53<br>P = 0.20	Coef. = -9.26<br>SE = 2.99<br>P = 2.9E-3	Coef. = -3.96<br>SE = 0.78<br>P = 2.2E-6	Coef. = -6.39<br>SE = 1.70<br>P = 1.4E-3	NA
	IT	NA	Coef. = -3.09<br>SE = 0.65<br>P = 2.6E-6	Coef. = -1.40<br>SE = 0.58<br>P = 0.016	Coef. = -6.73<br>SE = 1.28<br>P = 2.5E-7	Coef. = -5.41<br>SE = 0.72<br>P = 1.2E-13	Coef. = -6.78<br>SE = 2.02<br>P = 8.8E-4	NA
1.5 calipers (Ext. Data Fig. 2c)	SPA	Coef. = -5.77<br>SE = 1.45<br>P = 7.7E-5	Coef.= -5.79<br>SE = 1.68<br>P = 6.9E-4	Coef. = -1.03<br>SE = 0.78.<br>P = 0.076	Coef. = -8.69<br>SE = 2.85<br>P = 9.3E-3	Coef. = -11.61<br>SE = 1.88<br>P = 4.9E-9	Coef. = -64.67<br>SE = 36.13<br>P = 0.22	Coef. = -4.47<br>SE = 1.41<br>P = 2.4E-3
	SUPA	Coef. = -1.68<br>SE = 0.30<br>P = 2.0E-8	Coef. = -1.25<br>SE = 0.35<br>P=4.5E-4	Coef. = 0.03<br>SE = 0.31<br>P = 0.92	Coef. = -6.66<br>SE = 1.82<br>P=3.1E-4	Coef. = -3.62<br>SE = 0.79<br>P = 7.7E-6	Coef. = -2.72<br>SE = 1.12<br>P = 0.017	Coef. = -4.27<br>SE = 1.56<br>P = 6.8E-3
	IT	Coef. = -4.73<br>SE = 0.73<br>P = 9.6E-11	Coef. = -1.92<br>SE = 0.57<br>P = 9.3E-4	Coef. = -1.53<br>SE = 0.55<br>P = 0.0057	Coef. = -5.37<br>SE = 1.44<br>P = 2.1E-4	Coef. = -5.33<br>SE = 0.66<br>P = 8.6E-15	Coef. = -7.31<br>SE = 2.68<br>P = 0.0069	Coef. = -2.61<br>SE = 0.87<br>P = 0.0030
50% threshold (Ext. Data Fig. 2d)	SPA	Coef. = -10.36<br>SE = 2.70<br>P = 1.5E-4	NA	NA	NA	NA	NA	NA
	SUPA	Coef. = -2.14<br>SE = 0.40<br>P = 1.4E-7	Coef. = -1.49<br>SE = 0.39<br>P = 1.8E-4	Coef. = -0.39<br>SE = 0.34<br>P = 0.26	Coef. = -6.75<br>SE = 2.13<br>P = 1.8E-3	Coef. = -4.94<br>SE = 0.56<br>P = 1.5E-14	Coef. = -5.46<br>SE = 2.32<br>P = 0.021	Coef. = -5.79<br>SE = 1.57<br>P = 3.8E-4
	IT	Coef. = -6.75<br>SE = 0.80<br>P = 7.4E-17	Coef. = -3.20<br>SE = 0.69<br>P = 5.5E-6	Coef. = -0.73<br>SE = 1.04<br>P = 0.48	Coef. = -11.37<br>SE = 1.65<br>P = 2.4E-11	Coef. = -7.25<br>SE = 0.70<br>P = 1.3E-22	Coef. = -9.86<br>SE = 2.19<br>P = 1.4E-5	Coef. = -2.91<br>SE = 1.08<br>P = 7.5E-3
Dominated by pasture (Ext. Data Fig. 2e)	SPA	NA	NA	NA	Coef. = -10.08<br>SE = 1.60<br>P = 3.5E-7	Coef. = -10.84<br>SE = 2.14<br>P = 8.4E-7	Coef. = -3.17<br>SE = 3.53<br>P = 0.39	NA
	SUPA	NA	NA	Coef. = 0.21<br>SE = 1.18<br>P = 0.86	Coef. = -4.74<br>SE = 1.23<br>P = 1.6E-4	Coef. = -6.29<br>SE = 1.03<br>P = 5.7E-9	Coef. = -5.94<br>SE = 1.82<br>P = 1.3E-3	NA
	IT	NA	NA	Coef. = -1.77<br>SE = 1.43<br>P = 0.22	Coef. = -11.69<br>SE = 1.58<br>P = 7.8E-13	Coef. = -5.070<br>SE = 0.66<br>P = 8.2E-14	Coef. = -7.32<br>SE = 1.71<br>P = 2.5E-5	NA
Hansen data (Ext. Data Fig. 2f)	SPA	Coef. = -3.28<br>SE = 1.16<br>P = 4.7E-3	Coef. = -4.58<br>SE = 1.30<br>P = 5.3E-4	Coef. = -1.11<br>SE = 1.38<br>P = 0.44	Coef. = -5.86<br>SE = 1.65<br>P = 1.3E-3	Coef. = -6.97<br>SE = 1.47<br>P = 3.2E-6	Coef. = -4.59<br>SE = 1.07<br>P = 4.1E-5	Coef. = -3.80<br>SE = 1.09<br>P = 8.3E-4
	SUPA	Coef. = -1.52<br>SE = 0.29<br>P = 1.6E-7	Coef. = -0.44<br>SE = 0.29<br>P = 0.14	Coef. = 0.15<br>SE = 0.29<br>P = 0.60	Coef. = -5.25<br>SE = 1.40<br>P = 2.0E-4	Coef. = -3.37<br>SE = 0.46<br>P = 7.0E-12	Coef. = -2.87<br>SE = 0.79<br>P = 3.9E-4	Coef. = -4.08<br>SE = 1.03<br>P = 1.1E-4
	IT	Coef. = -3.53<br>SE = 0.57<br>P = 7.0E-10	Coef. = -2.12<br>SE = 0.51<br>P = 3.5E-5	Coef. = -1.27<br>SE = 0.59<br>P = 0.033	Coef. = -4.33<br>SE = 1.00<br>P = 1.7E-5	Coef. = -3.47<br>SE = 0.58<br>P = 3.6E-9	Coef. = -3.17<br>SE = 1.39<br>P = 0.023	Coef. = -1.76<br>SE = 0.75<br>P = 0.020
Fixed effects panel model (Ext. Data Fig. 2g)	SPA	Coef. = 5.49<br>SE = 1.61<br>P = 6.8E-4<br>Adj. P = 0.01<br>N = 1,558	Coef. = 5.00<br>SE = 1.86<br>P = 0.0076<br>Adj. P = 0.03<br>N = 258	Coef. = 2.38<br>SE = 2.06<br>P = 0.26<br>Adj. P = 0.45<br>N = 25	Coef. = 6.28<br>SE = 3.11<br>P = 0.050<br>Adj. P = 0.15<br>N = 47	Coef. = 9.24<br>SE = 1.76<br>P = 2.8E-7<br>Adj. P = 0.00<br>N = 306	Coef. = 6.28<br>SE = 1.27<br>P = 3.2E-6<br>Adj. P = 0.00<br>N = 110	Coef. = 5.77<br>SE = 2.31<br>P = 0.015<br>Adj. P = 0.06<br>N = 88
	SUPA	Coef. = 1.64<br>SE = 0.38<br>P = 1.8E-5<br>Adj. P = 0.00<br>N = 1,235	Coef. = 0.97<br>SE = 0.48<br>P = 0.042<br>Adj. P = 0.14<br>N = 472	Coef. = -0.31<br>SE = 0.61<br>P = 0.61<br>Adj. P = 0.74<br>N = 220	Coef. = 10.51<br>SE = 2.92<br>P = 3.6E-4<br>Adj. P = 0.01<br>N = 498	Coef. = 6.32<br>SE = 0.79<br>P = 7.2E-14<br>Adj. P = 0.00<br>N = 238	Coef. = 6.59<br>SE = 2.36<br>P = 0.0059<br>Adj. P = 0.03<br>N = 199	Coef. = 6.84<br>SE = 2.99<br>P = 0.024<br>Adj. P = 0.09<br>N = 184
	IT	Coef. = 5.13<br>SE = 1.48<br>P = 5.5E-4<br>Adj. P = 0.01<br>N = 2,150	Coef. = 3.47<br>SE = 0.97<br>P = 4.1E-4<br>Adj. P = 0.01<br>N = 531	Coef. = 0.99<br>SE = 1.13<br>P = 0.38<br>Adj. P = 0.56<br>N = 325	Coef. = 10.27<br>SE = 3.11<br>P = 0.0010<br>Adj. P = 0.01<br>N = 457	Coef. = 4.89<br>SE = 1.35<br>P = 2.9E-4<br>Adj. P = 0.01<br>N = 709	Coef. = 6.15<br>SE = 2.06<br>P = 0.0031<br>Adj. P = 0.02<br>N = 343	Coef. = 5.21<br>SE = 2.05<br>P = 0.012<br>Adj. P = 0.05<br>N = 350

Supplementary Table 17. Regression results (two-sided) of robustness tests for the effect of treatment on income (R$), controlled for inflation. Adjusted P-values use the false discovery rate to correct for multiple testing. Please note that we only show adjusted P-values for the fixed effects panel model to mimic our main analysis.

Robustness test (Figure)	PA type	Control						
		**Non-PA**	**Sparsely populated**	**Very small**	**Small**	**Medium**	**Large**	**Mining**
1.5 calipers (Ext. Data Fig. 5a)	SPA	Coef. = 266<br>SE = 118<br>P = 0.025	Coef. = 131<br>SE = 71<br>P = 0.066	Coef.= -75<br>S.E.= 69<br>P = 0.081	Coef. = 345<br>SE = 93<br>P = 2.6E-3	Coef. = 322<br>SE = 112<br>P = 4.7E-3	Coef. = 4055<br>SE = 2457<br>P = 0.24	Coef. = 129<br>SE = 73<br>P = 0.080
	SUPA	Coef. = 0.50<br>SE = 17<br>P = 0.98	Coef. = -28<br>SE = 24<br>P = 0.24	Coef. = -60<br>SE = 73<br>P = 0.41	Coef. = -68<br>SE = 47<br>P = 0.15	Coef. = -41<br>SE = 31<br>P = 0.18	Coef. = -53<br>SE = 46<br>P = 0.25	Coef. = -71<br>SE = 26<br>P = 6.1E-3
	IT	Coef. = -82<br>SE = 30<br>P = 5.7E-3	Coef. = -47<br>SE = 36<br>P = 0.19	Coef. = -110<br>SE = 30<br>P = 2.7E-4	Coef. = -10<br>SE = 38.51<br>P = 0.80	Coef. = 5.6<br>SE = 34<br>P = 0.87	Coef. = -102<br>SE = 49<br>P = 0.04	Coef. = -10<br>SE = 63<br>P = 0.87
50% threshold (Ext. Data Fig. 6a)	SPA	Coef. = 143<br>SE = 91<br>P = 0.12	NA	NA	NA	NA	NA	NA
	SUPA	Coef. = 40<br>SE = 59<br>P = 0.49	Coef. = 42<br>SE = 62<br>P = 0.50	Coef. = 61<br>SE = 125<br>P = 0.63	Coef. = -31<br>SE = 79<br>P = 0.69	Coef. = -3.2<br>SE = 75<br>P = 0.97	Coef. = -82<br>SE = 165<br>P = 0.62	Coef. = -160<br>SE = 102<br>P = 0.12
	IT	Coef. = -137<br>SE = 28<br>P = 8.2E-7	Coef. = -80<br>SE = 32<br>P = 0.015	Coef. = -95<br>SE = 40<br>P = 0.020	Coef. = 9.8<br>SE = 42<br>P = 0.81	Coef. = -37<br>SE = 35<br>P = 0.29	Coef. = -107<br>SE = 60<br>P = 0.081	Coef. = -49<br>SE = 50<br>P = 0.34
Est. after 2006 (Ext. Data Fig. 7a)	SPA	NA	Coef. = 140<br>SE = 83<br>P = 0.092	Coef. = 240<br>SE = 362<br>P = 0.53	Coef. = 201<br>SE = 136<br>P = 0.15	Coef. = 342<br>SE = 108<br>P = 0.00	Coef. = 225<br>SE = 665<br>P = 0.74	NA
	SUPA	NA	Coef. = 115<br>SE = 98<br>P = 0.24	Coef. = 99<br>SE = 177<br>P = 0.58	Coef. = -26<br>SE = 177<br>P = 0.89	Coef. = 75<br>SE = 99<br>P = 0.45	Coef. = -687<br>SE = 339<br>P = 0.06	NA
	IT	NA	Coef. = -83<br>SE = 36<br>P = 0.021	Coef. = -98<br>SE = 29<br>P = 8.6E-4	Coef. = -32<br>SE = 40<br>P = 0.42	Coef. = -8.4<br>SE = 29<br>P = 0.77	Coef. = -149<br>SE = 55<br>P = 0.0077	NA
Dominated by pasture (Ext. Data Fig. 8a)	SPA	NA	NA	NA	Coef. = 315<br>SE = 140<br>P = 0.031	Coef. = 424<br>SE = 113<br>P = 2.2E-4	Coef. = 252<br>SE = 580<br>P = 0.67	NA
	SUPA	NA	NA	Coef. = 85<br>SE = 116<br>P = 0.46	Coef. = -25<br>SE = 63<br>P = 0.69	Coef. = 170<br>SE = 123<br>P = 0.17	Coef. = 2.7<br>SE = 78<br>P = 0.97	NA
	IT	NA	NA	Coef. =<br>-133.49<br>SE = 43.75<br>P = 2.7E-3	Coef. =<br>-107.43<br>SE = 38.27<br>P = 5.2E-3	Coef. =<br>21.49<br>SE = 34.41<br>P = 0.53	Coef. =<br>-126.96<br>SE = 45.49<br>P = 5.6E-3	NA
Fixed effects panel model (Ext. Data Fig. 10a)	SPA	Coef. = 310<br>SE = 103<br>P = 0.0025<br>Adj. P = 0.02<br>N = 1,558	Coef. = 377<br>SE = 179<br>P = 0.036<br>Adj. P = 0.13<br>N = 258	Coef. = 409<br>SE = 205<br>P = 0.062<br>Adj. P = 0.17<br>N = 25	Coef. = 171<br>SE = 137<br>P = 0.22<br>Adj. P = 0.39<br>N = 47	Coef. = 325<br>SE = 136<br>P = 0.018<br>Adj. P = 0.07<br>N = 306	Coef. = 206<br>SE = 106<br>P = 0.055<br>Adj. P = 0.16<br>N = 110	Coef. = 79<br>SE = 136<br>P = 0.56<br>Adj. P = 0.72<br>N = 88
	SUPA	Coef. = 26<br>SE = 49<br>P = 0.59<br>Adj. P = 0.73<br>N = 1,235	Coef. = -5.6<br>SE = 61<br>P = 0.93<br>Adj. P = 0.98<br>N = 472	Coef. = 4.4<br>SE = 55<br>P = 0.94<br>Adj. P = 0.98<br>N = 220	Coef. = 63<br>SE = 96<br>P = 0.51<br>Adj. P = 0.70<br>N = 498	Coef. = -109<br>SE = 91<br>P = 0.23<br>Adj. P = 0.41<br>N = 238	Coef. = 12<br>SE = 80<br>P = 0.88<br>Adj. P = 0.96<br>N = 199	Coef. = -141<br>SE = 85<br>P = 0.10<br>Adj. P = 0.24<br>N = 184
	IT	Coef. = -46<br>SE = 27<br>P = 0.091<br>Adj. P = 0.24<br>N = 2,150	Coef. = -61<br>SE = 36<br>P = 0.093<br>Adj. P = 0.24<br>N = 531	Coef. = -39<br>SE = 36<br>P = 0.28<br>Adj. P = 0.46<br>N = 325	Coef. = 31<br>SE = 47<br>P = 0.51<br>Adj. P = 0.70<br>N = 457	Coef. = -80<br>SE = 27<br>P = 0.0027<br>Adj. P = 0.02<br>N = 709	Coef. = -20<br>SE = 60<br>P = 0.75<br>Adj. P = 0.89<br>N = 343	Coef. = -96<br>SE = 107<br>P = 0.37<br>Adj. P = 0.56<br>N = 350
		**Main analysis**	**5 km buffer**	**10 km buffer**	**25 km buffer**	**50 km buffer**	**75 km buffer**	**100 km buffer**
Mining buffers (Ext. Data Fig. 11a)	SPA	Coef. = 115<br>SE = 61<br>P = 0.062	Coef. = 78<br>SE = 73<br>P = 0.28	Coef. = 94<br>SE = 71<br>P = 0.19	Coef. = 172<br>SE = 88<br>P = 0.051	NA	NA	NA
	SUPA	Coef. = 112<br>SE = 48<br>P = 0.021	Coef. = -53<br>SE = 44<br>P = 0.23	Coef. = -84<br>SE = 32<br>P = 0.0096	Coef. = 20<br>SE = 24<br>P = 0.40	Coef. = 126<br>SE = 55<br>P = 0.027	Coef. = 58<br>SE = 23<br>P = 0.013	Coef. = 15<br>SE = 32<br>P = 0.63
	IT	Coef. = -17<br>SE = 47<br>P = 0.71	Coef. = -75<br>SE = 30<br>P = 0.012	Coef. = -96<br>SE = 33<br>P = 3.4E-3	Coef. = 21<br>SE = 23<br>P = 0.37	Coef. = 19<br>SE = 26<br>P = 0.45	Coef. = 20<br>SE = 34<br>P = 0.55	Coef. = 1.7<br>SE = 44<br>P = 0.97

Supplementary Table 18. Regression results (two-sided) of robustness tests for the effect of treatment on income inequality (GINI coefficient). Adjusted P-values use the false discovery rate to correct for multiple testing. Please note that we only show adjusted P-values for the fixed effects panel model to mimic our main analysis.

Robustness test (Figure)	PA type	Control						
		**Non-PA**	**Sparsely populated**	**Very small**	**Small**	**Medium**	**Large**	**Mining**
1.5 calipers (Ext. Data Fig. 5b)	SPA	Coef. = -0.016<br>SE = 0.029<br>P = 0.57	Coef. = 0.018<br>SE = 0.030<br>P = 0.58	Coef. = -0.35<br>SE = 0.46<br>P = 0.29	Coef. = -0.13<br>SE = 0.052<br>P = 0.023	Coef. = 8.0E-3<br>SE = 0.055<br>P = 0.89	Coef. = 0.79<br>SE = 1.44<br>P = 0.64	Coef. = 0.051<br>SE = 0.035<br>P = 0.15
	SUPA	Coef. = 0.013<br>SE = 0.011<br>P = 0.23	Coef. = 0.017<br>SE = 0.013<br>P = 0.59	Coef. = -0.030<br>SE = 0.024<br>P = 0.16	Coef. = -0.13<br>SE = 0.025<br>P = 4.3E-7	Coef. = 0.056<br>SE = 0.028<br>P = 0.049	Coef. = -0.065<br>SE = 0.038<br>P = 0.090	Coef. = 0.040<br>SE = 0.022<br>P = 0.071
	IT	Coef. = -0.011<br>SE = 0.014<br>P = 0.43	Coef. = -0.042<br>SE = 0.018<br>P = 0.024	Coef. = -0.080<br>SE = 0.038<br>P = 0.037	Coef. = -0.085<br>SE = 0.025<br>P = 7.4E-4	Coef. = -0.016<br>SE = 0.022<br>P = 0.78	Coef. = -0.041<br>SE = 0.038<br>P = 0.28	Coef. = 0.085<br>SE = 0.041<br>P = 0.037
50% threshold (Ext. Data Fig. 6b)	SPA	Coef. = 0.697<br>SE = 0.04<br>P = 0.040	NA	NA	NA	NA	NA	NA
	SUPA	Coef. = 0.020<br>SE = 0.014<br>P = 0.10	Coef. = 0.027<br>SE = 0.013<br>P = 0.037	Coef. = -0.028<br>SE = 0.029<br>P = 0.33	Coef. = -0.10<br>SE = 0.027<br>P = 2.5E-4	Coef. = 0.14<br>SE = 0.033<br>P = 6.4E-5	Coef. = -0.034<br>SE = 0.029<br>P = 0.24	Coef. = 0.040<br>SE = 0.043<br>P = 0.35
	IT	Coef. = -0.031<br>SE = 0.020<br>P = 0.12	Coef. = -0.044<br>SE = 0.026<br>P = 0.090	Coef. = -0.091<br>SE = 0.040<br>P = 0.025	Coef. = -0.076<br>SE = 0.027<br>P = 0.012	Coef. = 0.016<br>SE = 0.030<br>P = 0.60	Coef. = 0.014<br>SE = 0.053<br>P = 0.79	Coef. = 0.14<br>SE = 0.032<br>P = 2.7E-5
Est. after 2006 (Ext. Data Fig. 7b)	SPA	NA	Coef. = 0.01<br>SE = 0.03<br>P = 0.67	Coef. = -0.23<br>SE = 0.14<br>P = 0.15	Coef. = -0.08<br>SE = 0.05<br>P = 0.14	Coef. = 0.09<br>SE = 0.04<br>P = 0.05	Coef. = -0.21<br>SE = 0.19<br>P = 0.29	NA
	SUPA	NA	Coef. = 0.03<br>SE = 0.02<br>P = 0.13	Coef. = 0.02<br>SE = 0.03<br>P = 0.58	Coef. = -0.21<br>SE = 0.03<br>P = 0.00	Coef. = 0.14<br>SE = 0.04<br>P = 0.00	Coef. = -0.18<br>SE = 0.07<br>P = 0.02	NA
	IT	NA	Coef. = -0.03<br>SE = 0.02<br>P = 0.12	Coef. = -0.11<br>SE = 0.03<br>P = 3.5E-5	Coef. = -0.08<br>SE = 0.02<br>P = 3.6E-4	Coef. = -0.03<br>SE = 0.02<br>P = 0.18	Coef. = -0.02<br>SE = 0.03<br>P = 0.47	NA
Dominated by pasture (Ext. Data Fig. 8b)	SPA	NA	NA	NA	Coef. = -0.07<br>SE = 0.06<br>P = 0.28	Coef. = -0.01<br>SE = 0.04<br>P = 0.86	Coef. = -0.22<br>SE = 0.21<br>P = 0.31	NA
	SUPA	NA	NA	Coef. = 0.02<br>SE = 0.04<br>P = 0.56	Coef. = -0.19<br>SE = 0.03<br>P = 6.5E-13	Coef. = 0.02<br>SE = 0.04<br>P = 0.62	Coef. = -0.03<br>SE = 0.02<br>P = 0.055	NA
	IT	NA	NA	Coef. = 0.00<br>SE = 0.04<br>P = 0.90	Coef. = -0.06<br>SE = 0.03<br>P = 0.028	Coef. = -0.05<br>SE = 0.02<br>P = 0.024	Coef. = -0.02<br>SE = 0.03<br>P = 0.59	NA
Fixed effects panel model (Ext. Data Fig. 10b)	SPA	Coef. = 0.01<br>SE = 0.04<br>P = 0.87<br>Adj. P = 0.96	Coef. = 0.06<br>SE = 0.04<br>P = 0.16<br>Adj. P = 0.33	Coef. = -0.01<br>SE = 0.12<br>P = 0.95<br>Adj. P = 0.99	Coef. = -0.13<br>SE = 0.07<br>P = 0.088<br>Adj. P = 0.24	Coef. = 0.15<br>SE = 0.10<br>P = 0.12<br>Adj. P = 0.26	Coef. = -0.01<br>SE = 0.05<br>P = 0.86<br>Adj. P = 0.95	Coef. = 0.09<br>SE = 0.06<br>P = 0.13<br>Adj. P = 0.29
	SUPA	Coef. = 0.02<br>SE = 0.01<br>P = 0.18<br>Adj. P = 0.35	Coef. = 0.02<br>SE = 0.02<br>P = 0.43<br>Adj. P = 0.60	Coef. = 0.01<br>SE = 0.03<br>P = 0.84<br>Adj. P = 0.94	Coef. = -0.06<br>SE = 0.05<br>P = 0.28<br>Adj. P = 0.46	Coef. = 0.17<br>SE = 0.06<br>P = 0.0052<br>Adj. P = 0.03	Coef. = 0.09<br>SE = 0.04<br>P = 0.054<br>Adj. P = 0.16	Coef. = 0.07<br>SE = 0.05<br>P = 0.14<br>Adj. P = 0.30
	IT	Coef. = -0.02<br>SE = 0.02<br>P = 0.18<br>Adj. P = 0.34	Coef. = -0.04<br>SE = 0.02<br>P = 0.10<br>Adj. P = 0.24	Coef. = -0.03<br>SE = 0.03<br>P = 0.36<br>Adj. P = 0.55	Coef. = -0.06<br>SE = 0.02<br>P = 0.011<br>Adj. P = 0.05	Coef. = 0.01<br>SE = 0.04<br>P = 0.82<br>Adj. P = 0.94	Coef. = 0.07<br>SE = 0.05<br>P = 0.12<br>Adj. P = 0.26	Coef. = 0.13<br>SE = 0.04<br>P = 0.0031<br>Adj. P = 0.02
		**Main analysis**	**5 km buffer**	**10 km buffer**	**25 km buffer**	**50 km buffer**	**75 km buffer**	**100 km buffer**
Mining buffers (Ext. Data Fig. 11b)	SPA	Coef. = 0.05<br>SE = 0.03<br>P = 0.11	Coef. = 0.02<br>SE = 0.04<br>P = 0.52	Coef. = 0.05<br>SE = 0.04<br>P = 0.23	Coef. = -0.01<br>SE = 0.04<br>P = 0.89	NA	NA	NA
	SUPA	Coef. = 0.04<br>SE = 0.02<br>P = 0.12	Coef. = -0.03<br>SE = 0.03<br>P = 0.28	Coef. = 0.01<br>SE = 0.02<br>P = 0.58	Coef. = 0.00<br>SE = 0.02<br>P = 0.91	Coef. = -0.07<br>SE = 0.06<br>P = 0.30	Coef. = 0.03<br>SE = 0.02<br>P = 0.15	Coef. = 0.01<br>SE = 0.03<br>P = 0.66
	IT	Coef. = 0.09<br>SE = 0.03<br>P = 0.012	Coef. = -0.02<br>SE = 0.02<br>P = 0.39	Coef. = 0.02<br>SE = 0.02<br>P = 0.25	Coef. = 0.03<br>SE = 0.02<br>P = 0.14	Coef. = 0.00<br>SE = 0.02<br>P = 0.89	Coef. = 0.01<br>SE = 0.03<br>P = 0.75	Coef. = -0.02<br>SE = 0.03<br>P = 0.56

Supplementary Table 19. Regression results (two-sided) of robustness tests for the effect of treatment on the proportion of households with poor sanitation. Adjusted P-values use the false discovery rate to correct for multiple testing. Please note that we only show adjusted P-values for the fixed effects panel model to mimic our main analysis.

Robustness test (Figure)	PA type	Control						
		**Non-PA**	**Sparsely populated**	**Very small**	**Small**	**Medium**	**Large**	**Mining**
1.5 calipers (Ext. Data Fig. 5c)	SPA	Coef. = 0.05<br>SE = 0.03<br>P = 0.084	Coef. = 0.13<br>SE = 0.05<br>P = 0.0066	Coef. = 0.01<br>S.E. = 0.03<br>P = 0.27	Coef. = -0.02<br>SE = 0.03<br>P = 0.52	Coef. = -0.01<br>SE = 0.01<br>P = 0.48	Coef. = 0.27<br>SE = 0.49<br>P = 0.63	Coef. = -0.03<br>SE = 0.02<br>P = 0.037
	SUPA	Coef. = 0.01<br>SE = 0.01 P<br>= 0.46	Coef. = 0.00<br>SE = 0.01<br>P = 0.77	Coef. = -0.03<br>SE = 0.01<br>P = 0.015	Coef. = 0.01<br>SE = 0.02<br>P = 0.44	Coef. = 0.00<br>SE = 0.01<br>P = 0.69	Coef. = 0.01<br>SE = 0.02<br>P = 0.55	Coef. = -0.02<br>SE = 0.01<br>P = 0.12
	IT	Coef. = 0.01<br>SE = 0.01<br>P = 0.34	Coef. = 0.01<br>SE = 0.01<br>P = 0.60	Coef. = -0.04<br>SE = 0.02<br>P = 0.076	Coef. = -0.02<br>SE = 0.02<br>P = 0.36	Coef. = 0.03<br>SE = 0.02<br>P = 0.18	Coef. = -0.05<br>SE = 0.04<br>P = 0.13	Coef. = -0.04<br>SE = 0.02<br>P = 0.085
50% threshold (Ext. Data Fig. 6c)	SPA	Coef. =<br>0.031<br>SE = 0.032<br>P = 0.29	NA	NA	NA	NA	NA	NA
	SUPA	Coef. =<br>0.013<br>SE = 0.011<br>P =0.24	Coef. = -0.01<br>SE = 0.012<br>P = 0.52	Coef. =<br>-0.028<br>SE = 0.01<br>P = 7.2E-3	Coef. =<br>4.0E-3<br>SE = 0.012<br>P = 0.80	Coef. =<br>0.013<br>SE = 0.013<br>P = 0.32	Coef. =<br>0.015<br>SE = 0.018<br>P = 0.40	Coef. =<br>-0.017<br>SE = 0.013<br>P = 0.20
	IT	Coef. =<br>0.029<br>SE = 0.013<br>P = 0.026	Coef. = 0.00<br>SE = 0.015<br>P = 0.74	Coef. =<br>-0.019<br>SE = 0.021<br>P = 0.37	Coef. =<br>-0.028<br>SE = 0.020<br>P = 0.15	Coef. =<br>0.017<br>SE = 0.023<br>P = 0.46	Coef. =<br>-0.011<br>SE = 0.036<br>P = 0.85	Coef. =<br>-4.0E-3<br>SE = 0.017<br>P = 0.83
Est. after 2006 (Ext. Data Fig. 7c)	SPA	NA	Coef. = 0.14<br>SE = 0.05<br>P = 0.0053	Coef. = 0.01<br>SE = 0.03<br>P = 0.79	Coef. = -0.01<br>SE = 0.02<br>P = 0.53	Coef. = -0.01<br>SE = 0.02<br>P = 0.38	Coef. = -0.04<br>SE = 0.03<br>P = 0.21	NA
	SUPA	NA	Coef. = -0.01<br>SE = 0.01<br>P = 0.46	Coef. = -0.02<br>SE = 0.01<br>P = 0.08	Coef. = 0.02<br>SE = 0.01<br>P = 0.053	Coef. = 0.00<br>SE = 0.01<br>P = 0.64	Coef. = 0.03<br>SE = 0.03<br>P = 0.29	NA
	IT	NA	Coef. = 0.01<br>SE = 0.01<br>P = 0.37	Coef. = -0.04<br>SE = 0.02<br>P = 0.053	Coef. = 0.00<br>SE = 0.02<br>P = 0.82	Coef. = 0.03<br>SE = 0.02<br>P = 0.11	Coef. = -0.04<br>SE = 0.03<br>P = 0.17	NA
Dominated by pasture (Ext. Data Fig. 8c)	SPA	NA	NA	NA	Coef. = -0.01<br>SE = 0.02<br>P = 0.52	Coef. = 0.00<br>SE = 0.03<br>P = 0.92	Coef. = -0.03<br>SE = 0.03<br>P = 0.40	NA
	SUPA	NA	NA	Coef. = -0.01<br>SE = 0.03<br>P = 0.76	Coef. = -0.01<br>SE = 0.01<br>P = 0.31	Coef. = -0.01<br>SE = 0.01<br>P = 0.37	Coef. = 0.01<br>SE = 0.02<br>P = 0.65	NA
	IT	NA	NA	Coef. = -0.02<br>SE = 0.03<br>P = 0.50	Coef. = 0.00<br>SE = 0.02<br>P = 0.94	Coef. = 0.03<br>SE = 0.02<br>P = 0.15	Coef. = -0.05<br>SE = 0.03<br>P = 0.084	NA
Fixed effects panel model (Ext. Data Fig. 10c)	SPA	Coef. = -0.08<br>SE = 0.07<br>P = 0.22<br>Adj. P = 0.39	Coef. = -0.12<br>SE = 0.08<br>P = 0.11<br>Adj. P = 0.26	Coef. = -0.04<br>SE = 0.23<br>P = 0.86<br>Adj. P = 0.95	Coef. = -0.04<br>SE = 0.12<br>P = 0.76<br>Adj. P = 0.90	Coef. = -0.27<br>SE = 0.13<br>P = 0.040<br>Adj. P = 0.14	Coef. = -0.06<br>SE = 0.10<br>P = 0.54<br>Adj. P = 0.72	Coef. = -0.12<br>SE = 0.12<br>P = 0.32<br>Adj. P = 0.51
	SUPA	Coef. = 0.00<br>SE = 0.04<br>P = 0.98<br>Adj. P = 0.99	Coef. = 0.00<br>SE = 0.05<br>P = 0.99<br>Adj. P = 0.99	Coef. = 0.06<br>SE = 0.10<br>P = 0.56<br>Adj. P = 0.72	Coef. = 0.06<br>SE = 0.11<br>P = 0.58<br>Adj. P = 0.73	Coef. = -0.21<br>SE = 0.08<br>P = 0.0058<br>Adj. P = 0.03	Coef. = 0.02<br>SE = 0.06<br>P = 0.73<br>Adj. P = 0.88	Coef. = 0.05<br>SE = 0.08<br>P = 0.52<br>Adj. P = 0.70
	IT	Coef. = -0.02<br>SE = 0.04<br>P = 0.57<br>Adj. P = 0.72	Coef. = 0.00<br>SE = 0.06<br>P = 0.97<br>Adj. P = 0.99	Coef. = 0.07<br>SE = 0.06<br>P = 0.22<br>Adj. P = 0.39	Coef. = 0.10<br>SE = 0.12<br>P = 0.41<br>Adj. P = 0.59	Coef. = -0.11<br>SE = 0.06<br>P = 0.047<br>Adj. P = 0.15	Coef. = 0.00<br>SE = 0.06<br>P = 0.96<br>Adj. P = 0.99	Coef. = -0.02<br>SE = 0.09<br>P = 0.81<br>Adj. P = 0.94
			**5 km buffer**	**10 km buffer**	**25 km buffer**	**50 km buffer**	**75 km buffer**	**100 km buffer**
Mining buffers (Ext. Data Fig. 11c)	SPA	Coef. = -0.02<br>SE = 0.02<br>P = 0.17	Coef. = 0.00<br>SE = 0.02<br>P = 0.93	Coef. = 0.00<br>SE = 0.02<br>P = 0.86	Coef. = -0.02<br>SE = 0.02<br>P = 0.23	NA	NA	NA
	SUPA	Coef. = -0.01<br>SE = 0.01<br>P = 0.45	Coef. = 0.00<br>SE = 0.01<br>P = 0.65	Coef. = 0.00<br>SE = 0.01<br>P = 0.67	Coef. = 0.00<br>SE = 0.01<br>P = 0.64	Coef. = 0.01<br>SE = 0.03<br>P = 0.79	Coef. = 0.00<br>SE = 0.01<br>P = 0.83	Coef. = 0.00<br>SE = 0.01<br>P = 0.99
	IT	Coef. = -0.01<br>SE = 0.02<br>P = 0.72	Coef. = 0.02<br>SE = 0.02<br>P = 0.21	Coef. = 0.02<br>SE = 0.02<br>P = 0.27	Coef. = 0.01<br>SE = 0.02<br>P = 0.46	Coef. = -0.02<br>SE = 0.01<br>P = 0.15	Coef. = -0.02<br>SE = 0.02<br>P = 0.26	Coef. = 0.00<br>SE = 0.02<br>P = 0.85

Supplementary Table 20. Regression results (two-sided) of robustness tests for the effect of treatment on literacy rate. Adjusted P-values use the false discovery rate to correct for multiple testing. Please note that we only show adjusted P-values for the fixed effects panel model to mimic our main analysis.

Robustness test (Figure)	PA type	Control						
		**Non-PA**	**Sparsely populated**	**Very small**	**Small**	**Medium**	**Large**	**Mining**
1.5 calipers (Ext. Data Fig. 5d)	SPA	Coef. = 0.03<br>SE = 0.01<br>P = 0.90	Coef. = 0.04<br>SE = 0.02<br>P = 0.73	Coef. = -0.24<br>S.E. = 0.26<br>P = 0.23	Coef. = -0.03<br>SE = 0.02<br>P = 0.22	Coef. = -0.03<br>SE = 0.02<br>P = 0.12	Coef. = -0.04<br>SE = 0.02<br>P = 0.13	Coef. = -0.01<br>SE = 0.02<br>P = 0.70
	SUPA	Coef. = 0.03<br>SE = 0.01 P = 0.026	Coef. = 0.04<br>SE = 0.02<br>P = 0.023	Coef. = 0.01<br>SE = 0.03<br>P = 0.59	Coef. = -0.03<br>SE = 0.02 P = 0.22	Coef. = -0.03<br>SE = 0.02<br>P = 0.12	Coef. = -0.04<br>SE = 0.02<br>P = 0.13	Coef. = -0.01<br>SE = 0.02<br>P = 0.70
	IT	Coef. = 0.01<br>SE = 0.02<br>P = 0.80	Coef. = 0.03<br>SE = 0.03<br>P = 0.26	Coef. = 0.02<br>SE = 0.02<br>P = 0.33	Coef. = -0.11<br>SE = 0.03<br>P = 1.9E-4	Coef. = 0.02<br>SE = 0.03<br>P = 0.51	Coef. = -0.03<br>SE = 0.03<br>P = 0.36	Coef. = 0.02<br>SE = 0.03<br>P = 0.52
50% threshold (Ext. Data Fig. 6d)	SPA	Coef. = -0.027<br>SE = 0.027<br>P = 0.31	NA	NA	NA	NA	NA	NA
	SUPA	Coef. = 0.027<br>SE = 0.015<br>P = 0.068	Coef. = 0.025<br>SE = 0.016<br>P = 0.13	Coef. = 0.054<br>SE = 0.023<br>P = 0.025	Coef. = -0.042<br>SE = 0.022<br>P = 0.061	Coef. = -0.053<br>SE = 0.023<br>P = 0.014	Coef. = -0.011<br>SE = 0.032<br>P = 0.87	Coef. = -0.014<br>SE = 0.023<br>P = 0.83
	IT	Coef. = -0.0090<br>SE = 0.028<br>P = 0.75	Coef. = 0.003<br>SE = 0.037<br>P = 0.93	Coef. = 0.025<br>SE = 0.037<br>P = 0.53	Coef. = -0.12<br>SE = 0.036<br>P = 1.2E-3	Coef. = 0.026<br>SE = 0.031<br>P = 0.40	Coef. = -0.054<br>SE = 0.059<br>P = 0.36	Coef. = 0.045<br>SE = 0.030<br>P = 0.13
Est. after 2006 (Ext. Data Fig. 7d)	SPA	NA	Coef. = 0.00<br>SE = 0.02<br>P = 0.95	Coef. = -0.06<br>SE = 0.15<br>P = 0.72	Coef. = -0.03<br>SE = 0.04<br>P = 0.38	Coef. = -0.05<br>SE = 0.03<br>P = 0.039	Coef. = -0.11<br>SE = 0.11<br>P = 0.32	NA
	SUPA	NA	Coef. = 0.02<br>SE = 0.02<br>P = 0.30	Coef. = 0.03<br>SE = 0.02<br>P = 0.18	Coef. = -0.02<br>SE = 0.03<br>P = 0.47	Coef. = -0.05<br>SE = 0.03<br>P = 0.10	Coef. = -0.03<br>SE = 0.05<br>P = 0.59	NA
	IT	NA	Coef. = 0.00<br>SE = 0.02<br>P = 0.94	Coef. = 0.03<br>SE = 0.02<br>P = 0.21	Coef. = -0.11<br>SE = 0.03<br>P = 0.00	Coef. = 0.02<br>SE = 0.03<br>P = 0.41	Coef. =-0.01<br>SE = 0.04<br>P = 0.90	NA
Dominated by pasture (Ext. Data Fig. 8d)	SPA	NA	NA	NA	Coef. = -0.01<br>SE = 0.04<br>P = 0.90	Coef. = -0.03<br>SE = 0.03<br>P = 0.40	Coef. = -0.21<br>SE = 0.11<br>P = 0.080	NA
	SUPA	NA	NA	Coef. = -0.03<br>SE = 0.04<br>P = 0.51	Coef. = -0.03<br>SE = 0.03<br>P = 0.22	Coef. = 0.02<br>SE = 0.02<br>P = 0.44	Coef. = 0.01<br>SE = 0.02<br>P = 0.81	NA
	IT	NA	NA	Coef. = 0.04<br>SE = 0.04<br>P = 0.32	Coef. = -0.05<br>SE = 0.02<br>P = 0.031	Coef. = 0.04<br>SE = 0.04<br>P = 0.26	Coef. = -0.03<br>SE = 0.04<br>P = 0.48	NA
Fixed effects panel model (Ext. Data Fig. 10d)	SPA	Coef. = 0.03<br>SE = 0.03<br>P = 0.29<br>Adj. P = 0.46	Coef. = -0.06<br>SE = 0.05<br>P = 0.21<br>Adj. P = 0.39	Coef. = 0.14<br>SE = 0.05<br>P = 0.014<br>Adj. P = 0.06	Coef. = -0.10<br>SE = 0.17<br>P = 0.56<br>Adj. P = 0.72	Coef. = 0.04<br>SE = 0.03<br>P = 0.098<br>Adj. P = 0.24	Coef. = -0.03<br>SE = 0.04<br>P = 0.51<br>Adj. P = 0.70	Coef. = 0.04<br>SE = 0.04<br>P = 0.33<br>Adj. P = 0.51
	SUPA	Coef. = 0.01<br>SE = 0.01<br>P = 0.35<br>Adj. P = 0.54	Coef. = 0.00<br>SE = 0.02<br>P = 0.92<br>Adj. P = 0.98	Coef. = 0.07<br>SE = 0.03<br>P = 0.0062<br>Adj. P = 0.03	Coef. = -0.26<br>SE = 0.08<br>P = 5.9 E-4<br>Adj. P = 0.01	Coef. = -0.06<br>SE = 0.05<br>P = 0.17<br>Adj. P = 0.34	Coef. = -0.03<br>SE = 0.02<br>P = 0.12<br>Adj. P = 0.26	Coef. = -0.04<br>SE = 0.03<br>P = 0.13<br>Adj. P = 0.28
	IT	Coef. = 0.03<br>SE = 0.04<br>P = 0.40<br>Adj. P = 0.59	Coef. = 0.04<br>SE = 0.04<br>P = 0.27<br>Adj. P = 0.46	Coef. = 0.13<br>SE = 0.03<br>P = 3.9E-4<br>Adj. P = 0.01	Coef. = -0.18<br>SE = 0.06<br>P = 0.0030<br>Adj. P = 0.02	Coef. = 0.06<br>SE = 0.03<br>P = 0.048<br>Adj. P = 0.15	Coef. = 0.08<br>SE = 0.02<br>P = 6.1E-4<br>Adj. P = 0.01	Coef. = 0.01<br>SE = 0.03<br>P = 0.82<br>Adj. P = 0.94
			**5 km buffer**	**10 km buffer**	**25 km buffer**	**50km buffer**	**75 km buffer**	**100km buffer**
Mining buffers (Ext. Data Fig. 11d)	SPA	Coef. = 0.02<br>SE = 0.02<br>P = 0.42	Coef. = 0.01<br>SE = 0.02<br>P = 0.49	Coef. = 0.00<br>SE = 0.02<br>P = 0.85	Coef. = -0.02<br>SE = 0.03<br>P = 0.37	NA	NA	NA
	SUPA	Coef. = -0.02<br>SE = 0.02<br>P = 0.14	Coef. = 0.02<br>SE = 0.02<br>P = 0.34	Coef. = -0.05<br>SE = 0.02<br>P = 0.017	Coef. = -0.03<br>SE = 0.02<br>P = 0.15	Coef. = -0.06<br>SE = 0.04<br>P = 0.094	Coef. = 0.02<br>SE = 0.02<br>P = 0.28	Coef. = 0.02<br>SE = 0.02<br>P = 0.34
	IT	Coef. = 0.04<br>SE = 0.03<br>P = 0.23	Coef. = -0.02<br>SE = 0.02<br>P = 0.22	Coef. = -0.02<br>SE = 0.02<br>P = 0.18	Coef. = -0.01<br>SE = 0.02<br>P = 0.76	Coef. = 0.04<br>SE = 0.03<br>P = 0.15	Coef. = 0.07<br>SE = 0.03<br>P = 0.014	Coef. = 0.11<br>SE = 0.04<br>P = 0.014

Supplementary Figures

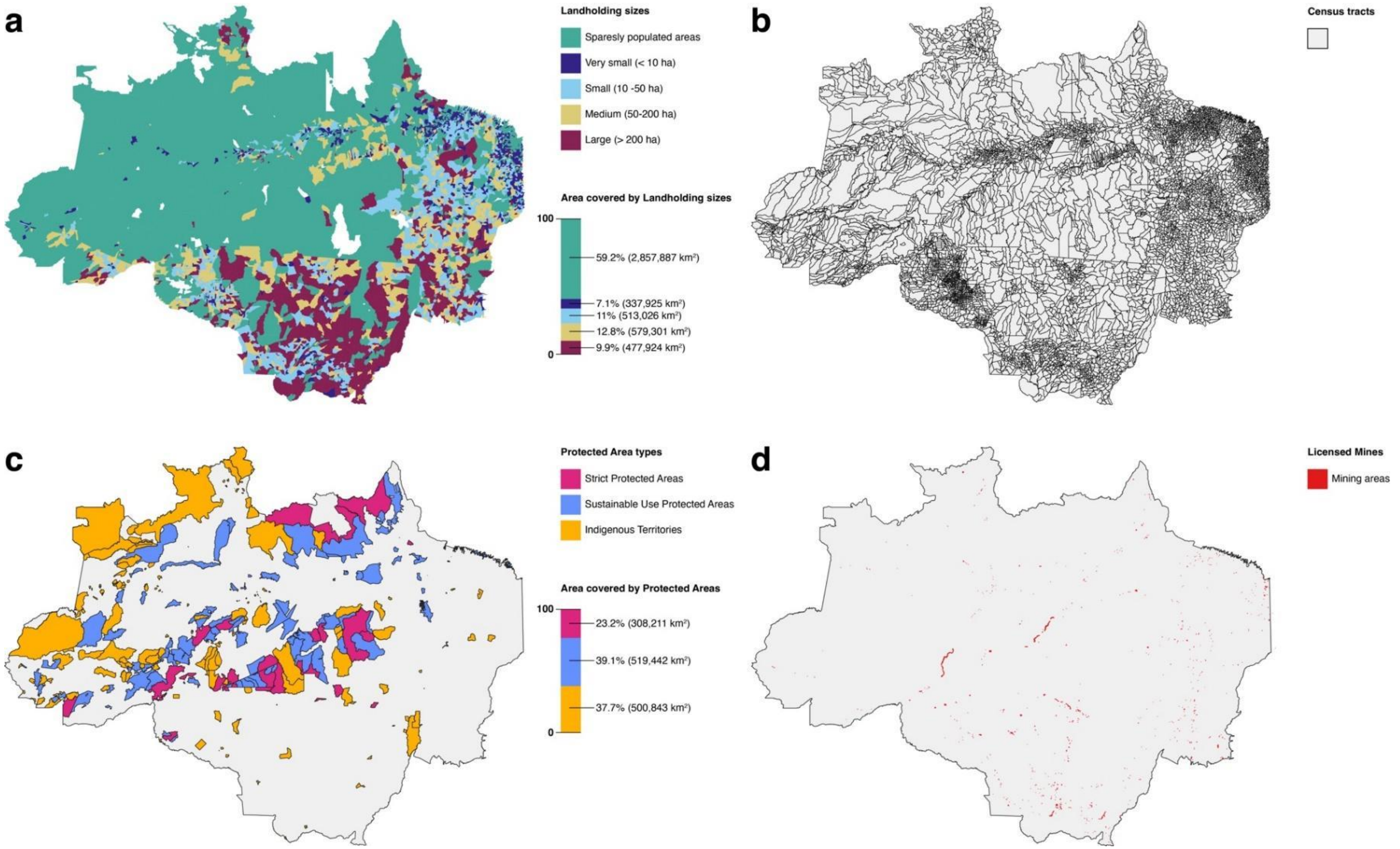

Supplementary Figure 1. **Maps of the Brazilian Legal Amazon**. **a** Landholding sizes based on the agricultural census in census tracts included in our analysis. The top legend shows landholding sizes by colour and the bottom legend shows the cumulative area covered by different landholding sizes (adapted from ref. 45). **b** All census tracts in the Brazilian Legal Amazon (adapted from ref. 41). **c** Protected area types established between 2000 and 2010 (adapted from ref. 56). **d** Licensed mining areas (adapted from ref. 47)

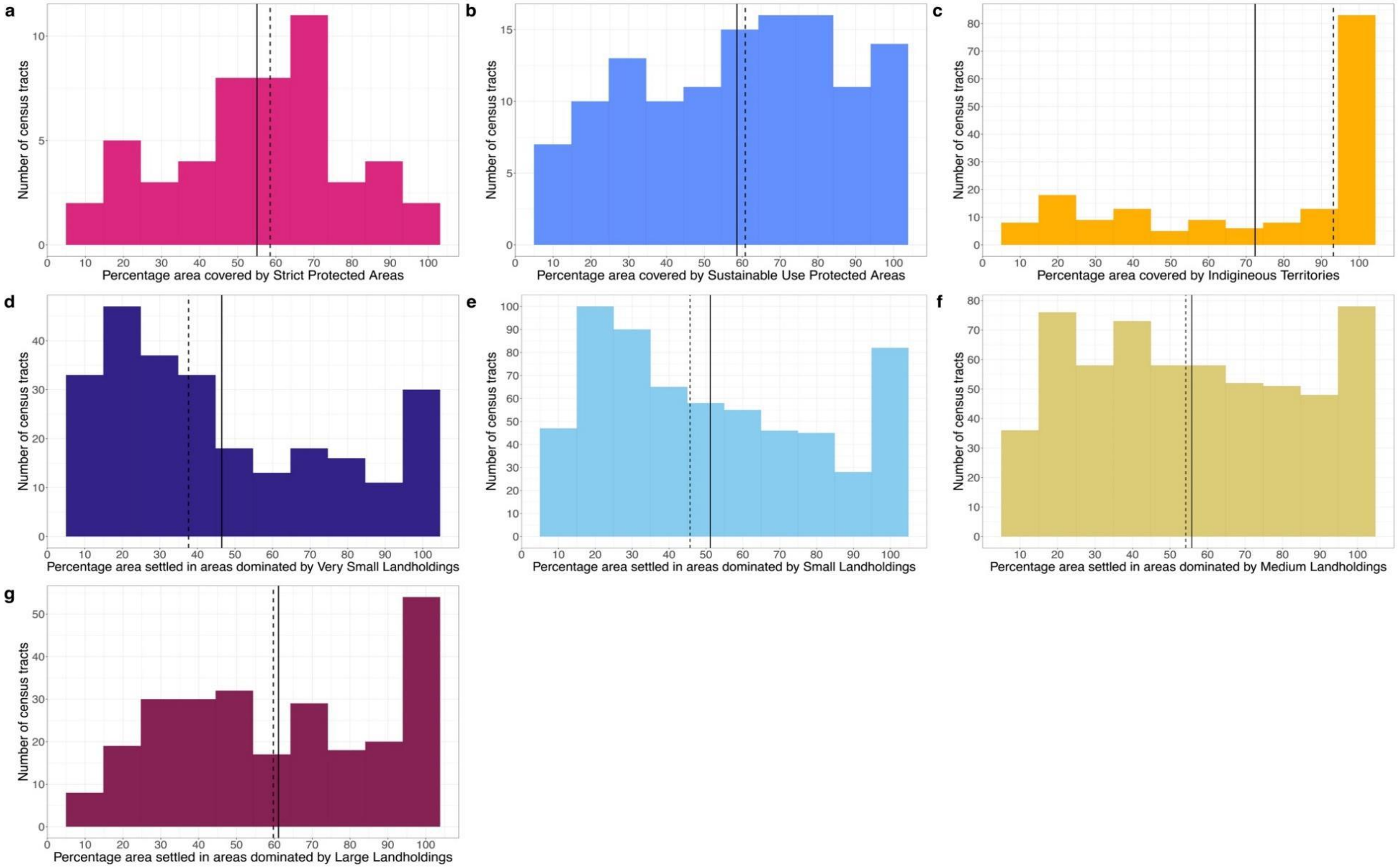

Supplementary Figure 2. Distribution of census tract area covered by protection categories and agricultural controls. a Strict Protected Areas. **b** Sustainable Use Protected Areas. **c** Indigenous Territories. **d** Very small landholdings (<10 ha). **e** Small landholdings (10-50 ha). **f** Medium landholdings (50-200 ha). **g** Large landholdings (>200 ha)**.** Dominant agricultural landholder size is determined by the category that contains the most properties per CT. Black solid lines represent distribution means and dashed lines distribution medians.

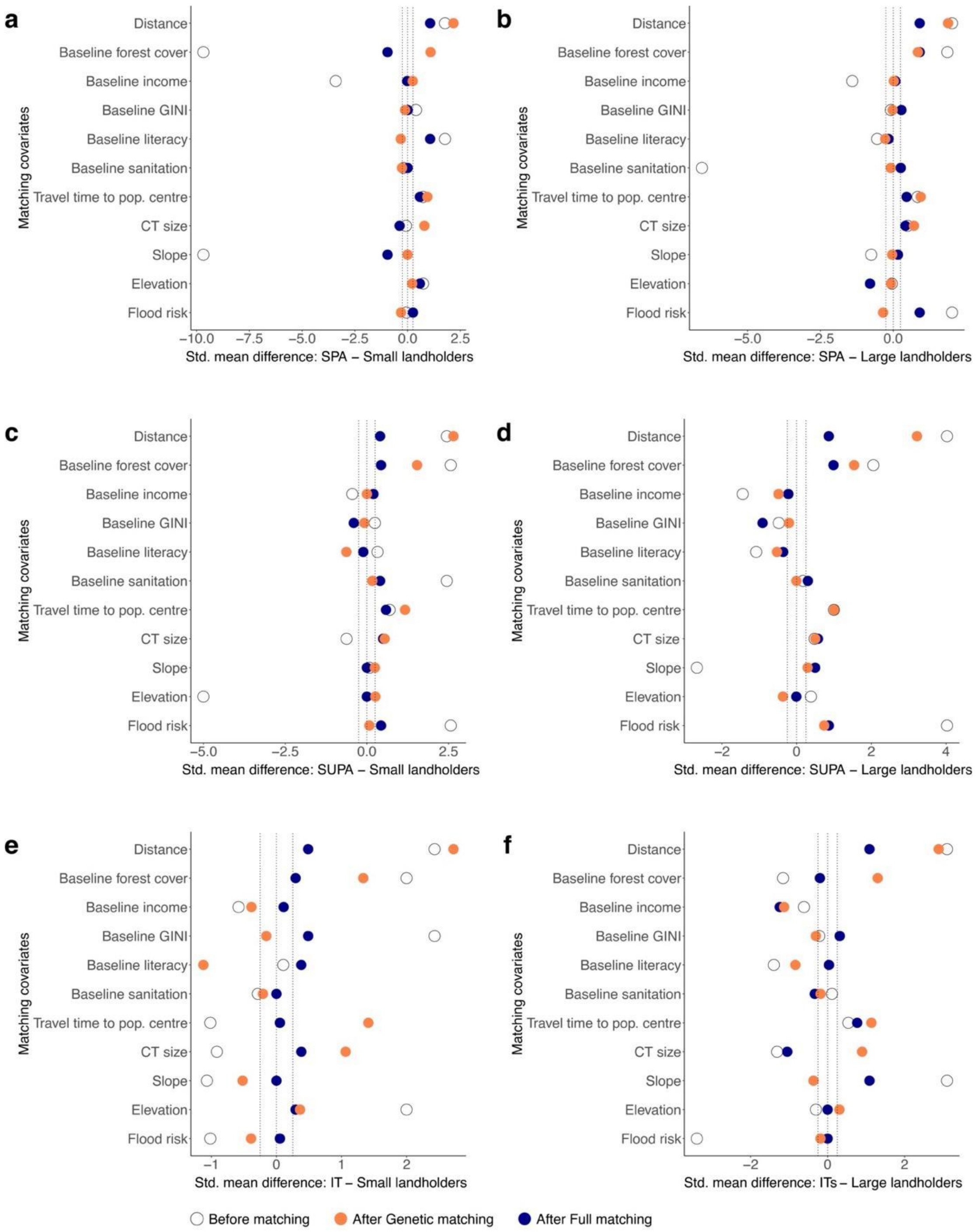

Supplementary Figure 3. Balance of covariates before and after full matching and after genetic matching for the worst performing matches. a Strict Protected Areas (SPAs) compared to small landholders. **b** SPAs compared to large landholders. **c** Sustainable Use Protected Areas (SUPAs) compared to small landholders. **d** SUPAs compared to large landholders. **e** Indigenous Territories (ITs) compared to small landholders. **f** ITs compared to large landholders. Standardized mean difference for confounding covariates before (open circles) and after full matching (blue circles) and after genetic matching (orange circles). Dotted lines indicate acceptable standard mean difference after matching to reach balance (Stuart 2010). Note, we exclude variables from these figures where we used exact matching (state, mines established before 2000 and mines established after 2000). See Supplementary Table 7 for an overview of which variables we included in each comparison.

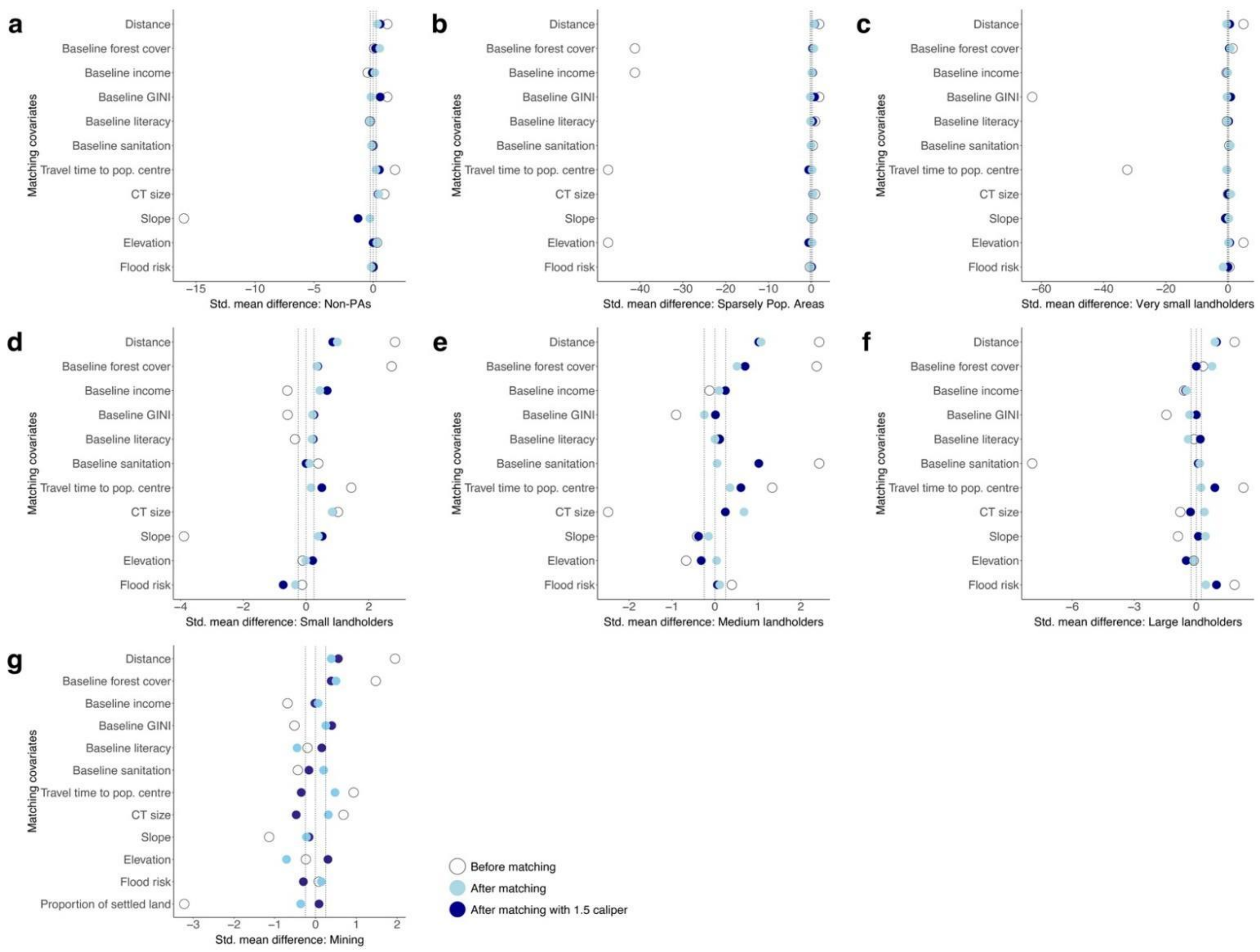

Supplementary Figure 4. Covariate balance between Strict Protected Areas (SPAs) and control units before and after full matching and after full matching using 1.5 calipers. a Non-protected controls. **b** Sparsely populated areas. **c** Very small landholders. **d** Small landholders. **e** Medium landholders. **f** Large landholders. **g** Licensed mining areas. Note, we exclude variables from these figures where we used exact matching (state, mines established before 2000 and mines established after 2000). See Supplementary Table 7 for an overview of which variables we included in each comparison.

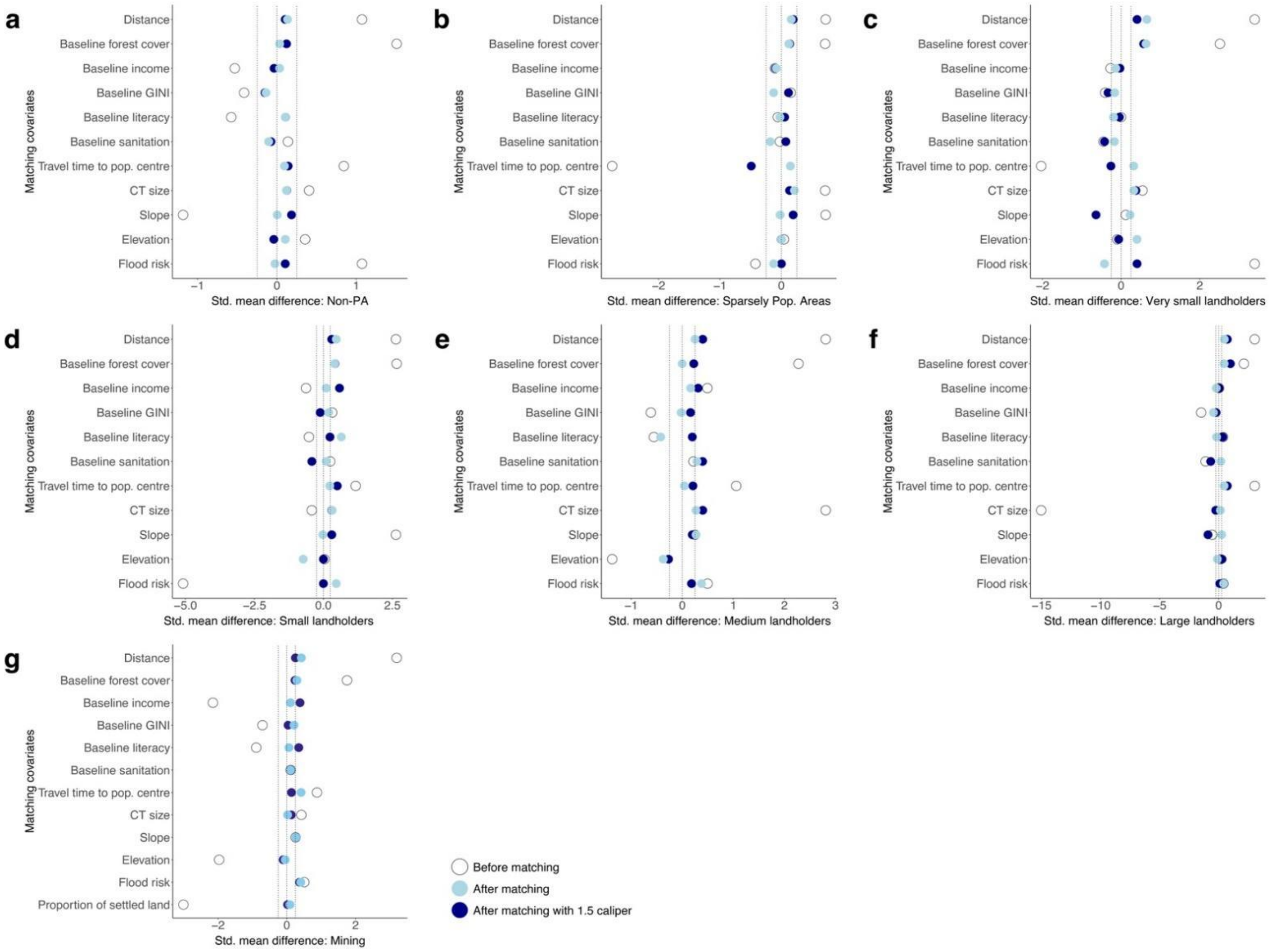

Supplementary Figure 5. Covariate balance between Sustainable Use Protected Areas (SUPAs) and control units before and after full matching and after full matching using 1.5 calipers. a Non-protected controls. **b** Sparsely populated areas. **c** Very small landholders. **d** Small landholders. **e** Medium landholders. **f** Large landholders. **g** Licensed mining areas. Note, we exclude variables from these figures where we used exact matching (state, mines established before 2000 and mines established after 2000). See Supplementary Table 7 for an overview of which variables we included in each comparison.

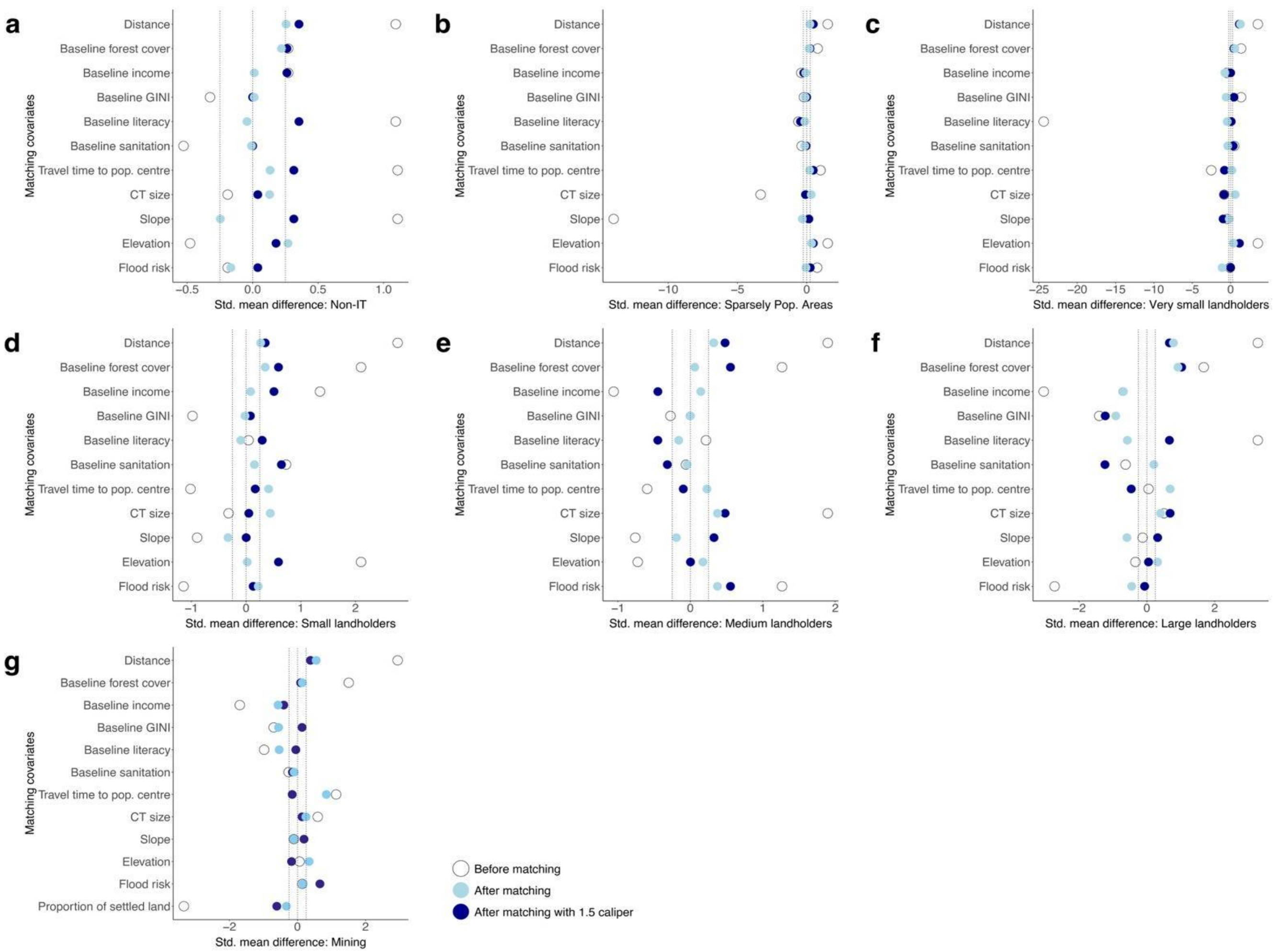

Supplementary Figure 6. Covariate balance between Indigenous Territories (ITs) and control units before and after full matching and after full matching using 1.5 calipers. a Non-protected controls. **b** Sparsely populated areas. **c** Very small landholders. **d** Small landholders. **e** Medium landholders. **f** Large landholders. **g** Licensed mining areas. Note, we exclude variables from these figures where we used exact matching (state, mines established before 2000 and mines established after 2000). See Supplementary Table 7 for an overview of which variables we included in each comparison.

References for Supplementary Information

1. Garrett, R. D. *et al.* Forests and Sustainable Development in the Brazilian Amazon: History, Trends, and Future Prospects. *https://doi.org/10.1146/annurev-environ-012220-010228* **46**, 625–652 (2021).
2. Joppa, L. N., Loarie, S. R. & Pimm, S. L. On the protection of "protected areas". *Proceedings of the National Academy of Sciences* **105**, 6673–6678 (2008).
3. Áreas Protegidas na Amazônia Brasileira: avanços e desafios. *Imazon* https://imazon.org.br/areas-protegidas-na-amazonia-brasileira-avancos-e-desafios-2/ (2012).
4. Ministério do Meio Ambiente. Categorias Unidades de Conservaçao. http://antigo.mma.gov.br/areas-protegidas/unidades-de-conservacao/categorias.html.
5. Marques, A. A. B. de, Schneider, M. & Peres, C. A. Human population and socioeconomic modulators of conservation performance in 788 Amazonian and Atlantic Forest reserves. *PeerJ* **4**, e2206 (2016).
6. Barbosa, L. C. The World-System and the Destruction of the Brazilian Amazon Rain Forest. *Review (Fernand Braudel Center)* **16**, 215–240 (1993).
7. Hoefle, S. W. Beyond carbon colonialism: Frontier peasant livelihoods, spatial mobility and deforestation in the Brazilian Amazon. *Critique of Anthropology* **33**, 193–213 (2013).
8. Lobo, F. D. L., Souza-Filho, P. W. M., Novo, E. M. L. de M., Carlos, F. M. & Barbosa, C. C. F. Mapping Mining Areas in the Brazilian Amazon Using MSI/Sentinel-2 Imagery (2017). *Remote Sensing* **10**, 1178 (2018).
9. Lloyd, T. J. *et al.* Multiple facets of biodiversity are threatened by mining-induced land-use change in the Brazilian Amazon. *Diversity and Distributions* **29**, 1190–1204 (2023).
10. Villén-Pérez, S., Anaya-Valenzuela, L., Conrado da Cruz, D. & Fearnside, P. M. Mining threatens isolated indigenous peoples in the Brazilian Amazon. *Global Environmental Change* **72**, 102398 (2022).
11. Situação jurídica das TIs no Brasil hoje. https://pib.socioambiental.org/pt/Situa%C3%A7%C3%A3o_jur%C3%ADdica_das_TIs_no_Brasil_hoje.
12. Vliet, N. van, Adams, C., Vieira, I. C. G. & Mertz, O. "Slash and Burn" and "Shifting" Cultivation Systems in Forest Agriculture Frontiers from the Brazilian Amazon. *Society & Natural Resources* (2013).

13. INPE. PRODES — Monitoramento da Floresta Amazônica Brasileira por Satélite. http://www.obt.inpe.br/prodes (2017).
14. Ministério do Meio Ambiente. Municípios Prioritários - PREVENÇÃO E CONTROLE DO DESMATAMENTO. http://combateaodesmatamento.mma.gov.br/municipios-prioritarios.
15. Heilmayr, R., Rausch, L. L., Munger, J. & Gibbs, H. K. Brazil's Amazon Soy Moratorium reduced deforestation. *Nat Food* **1**, 801–810 (2020).
16. Jusys, T. A confirmation of the indirect impact of sugarcane on deforestation in the Amazon. *Journal of Land Use Science* **12**, 125–137 (2017).
17. Gibbs, H. K. *et al.* Did Ranchers and Slaughterhouses Respond to Zero-Deforestation Agreements in the Brazilian Amazon? *Conservation Letters* **9**, 32–42 (2016).
18. Dyngeland, C., Oldekop, J. A. & Evans, K. L. Assessing multidimensional sustainability: Lessons from Brazil's social protection programs. *Proceedings of the National Academy of Sciences of the United States of America* **117**, 20511–20519 (2020).
19. Godar, J., Gardner, T. A., Tizado, E. J. & Pacheco, P. Actor-specific contributions to the deforestation slowdown in the Brazilian Amazon. *Proceedings of the National Academy of Sciences of the United States of America* **111**, 15591–6 (2014).
20. Alix-Garcia, J., Rausch, L. L., L'Roe, J., Gibbs, H. K. & Munger, J. Avoided Deforestation Linked to Environmental Registration of Properties in the Brazilian Amazon. *Conservation Letters* **11**, e12414 (2018).
21. Brancalion, P. H. S. *et al.* Fake legal logging in the Brazilian Amazon. *Science Advances* **4**, eaat1192 (2018).
22. Qin, Y. *et al.* Forest conservation in Indigenous territories and protected areas in the Brazilian Amazon. *Nat Sustain* **6**, 295–305 (2023).
23. Khanna, J., Medvigy, D., Fueglistaler, S. & Walko, R. Regional dry-season climate changes due to three decades of Amazonian deforestation. *Nature Clim Change* **7**, 200–204 (2017).
24. Oldekop, J. A. *et al.* Forest-linked livelihoods in a globalized world. *Nat. Plants* **6**, 1400–1407 (2020).
25. dos Reis, M., Graça, P. M. L. de A., Yanai, A. M., Ramos, C. J. P. & Fearnside, P. M. Forest fires and deforestation in the central Amazon: Effects of landscape and climate on spatial and temporal dynamics. *Journal of Environmental Management* **288**, 112310 (2021).
26. Brienen, R. J. W. *et al.* Long-term decline of the Amazon carbon sink. *Nature* **519**, 344–348 (2015).

27. Lovejoy, T. E. & Nobre, C. Amazon Tipping Point. *Science Advances* **4**, eaat2340 (2018).
28. Andam, K. S., Ferraro, P. J., Sims, K. R. E., Healy, A. & Holland, M. B. Protected areas reduced poverty in Costa Rica and Thailand. *Proceedings of the National Academy of Sciences of the United States of America* **107**, 9996–10001 (2010).
29. Canavire-Bacarreza, G. & Hanauer, M. M. Estimating the Impacts of Bolivia's Protected Areas on Poverty. *World Development* **41**, 265–285 (2013).
30. Assunção, J., Gandour, C. C. & Rocha, R. *Deforestation Slowdown in the Legal Amazon: Prices or Policies ?* (Climate Policy Initiative, Rio de Janeiro, Brazil, 2012).
31. Pailler, S. Re-election incentives and deforestation cycles in the Brazilian Amazon. *Journal of Environmental Economics and Management* **88**, 345–365 (2018).
32. zu Ermgassen, E. K. H. J. *et al.* The origin, supply chain, and deforestation risk of Brazil's beef exports. *Proceedings of the National Academy of Sciences* 202003270 (2020) doi:10.1073/pnas.2003270117.
33. Esquivel-Muelbert, A. *et al.* Tree mode of death and mortality risk factors across Amazon forests. *Nat Commun* **11**, 5515 (2020).
34. Bivand, R. Spatial Dependence: Weighting Schemes, Statistics and Models R package spdep. *Comprehensive R Archive Network* **version 0.**, (2015).
35. Oster, E. Unobservable Selection and Coefficient Stability: Theory and Evidence. *Journal of Business & Economic Statistics* **37**, 187–204 (2019).
36. Nepstad, D. *et al.* Slowing Amazon deforestation through public policy and interventions in beef and soy supply chains. *Science (New York, N.Y.)* **344**, 1118–23 (2014).
37. Hansen, M. C. *et al.* High-Resolution Global Maps of 21st-Century Forest Cover Change. *Science* **342**, 850–853 (2013).
38. Kalamandeen, M. *et al.* Pervasive Rise of Small-scale Deforestation in Amazonia. *Scientific Reports* **8**, 1600 (2018).
39. Benjamini, Y. & Hochberg, Y. Controlling the False Discovery Rate: A Practical and Powerful Approach to Multiple Testing. *Journal of the Royal Statistical Society: Series B (Methodological)* **57**, 289–300 (1995).
40. Sunderlin, W. D. *et al.* Livelihoods, forests, and conservation in developing countries: An Overview. *World Development* **33**, 1383–1402 (2005).
41. IBGE. Censo Demográfico. https://www.ibge.gov.br/estatisticas/sociais/populacao/9663-censo-demografico-2000.html.

42. Hassfurter, K. Safely Managed Drinking Water - Joint Monitoring Programme (JMP) report. *UNICEF DATA* https://data.unicef.org/resources/safely-managed-drinking-water/ (2017).
43. IBGE. Downloads | Geociências. https://www.ibge.gov.br/geociencias/downloads-geociencias.html.
44. Wittemyer, G., Elsen, P., Bean, W. T., Burton, A. C. O. & Brashares, J. S. Accelerated human population growth at protected area edges. *Science* **321**, 123–126 (2008).
45. IBGE. Censo Agropecuário. https://www.ibge.gov.br/estatisticas/economicas/agricultura-e-pecuaria/9827-censo-agropecuario.html?=&t=downloads.
46. Hecht, S. B. Environment, development and politics: Capital accumulation and the livestock sector in Eastern Amazonia. World Development 13, 663–684 (1985).
47. DNPM. Processos Minerarios: Sistema de Informações Geográficas da Mineração (SIGMINE). http://sigmine.dnpm.gov.br/ (2012).
48. Sonter, L. J., Ali, S. H. & Watson, J. E. M. Mining and biodiversity: key issues and research needs in conservation science. *Proceedings of the Royal Society B: Biological Sciences* **285**, 20181926 (2018).
49. Ali, S. H. *et al.* Mineral supply for sustainable development requires resource governance. *Nature* **543**, 367–372 (2017).
50. Nolte, C., Agrawal, A., Silvius, K. M. & Soares-Filho, B. S. Governance regime and location influence avoided deforestation success of protected areas in the Brazilian Amazon. *Proceedings of the National Academy of Sciences of the United States of America* **110**, 4956–4961 (2013).
51. Soares-Filho, B. S. *et al.* Modelling conservation in the Amazon basin. *Nature* **440**, 520–523 (2006).
52. Angelsen, A. Policies for reduced deforestation and their impact on agricultural production. *Proceedings of the National Academy of Sciences of the United States of America* **107**, 19639–19644 (2010).
53. SRTM 90m Digital Elevation Database v4.1. *CGIAR-CSI* https://cgiarcsi.community/data/srtm-90m-digital-elevation-database-v4-1/ (2017).
54. Werner, T. T. *et al.* Global-scale remote sensing of mine areas and analysis of factors explaining their extent. *Global Environmental Change* **60**, 102007 (2020).
55. ESA. GlobCover. http://due.esrin.esa.int/page_globcover.php.

56. Brasil. CNUC: Cadastro Nacional de Unidades de Conservação. https://dados.gov.br/dataset/unidadesdeconservacao/resource/c0babb3e-ec4e-4db5-a2b6-b79477260b0f (2022).
