# Supplementary material for: Socio-economic and environmental trade-offs in Amazonian protected areas and Indigenous territories revealed by assessing competing land uses
Source: Nat Ecol Evol. 2024 Jul 15;8(8):1482–92. doi: 10.1038/s41559-024-02458-w (PMC11310078; doi:10.1038/s41559-024-02458-w)
Supplement: Supplementary file 2 — Reporting Summary [file 41559_2024_2458_MOESM2_ESM.pdf]

nature portfolio

Corresponding author(s): Bowy den Braber

Last updated by author(s): 20-05-2024

Reporting Summary

Nature Portfolio wishes to improve the reproducibility of the work that we publish. This form provides structure for consistency and transparency in reporting. For further information on Nature Portfolio policies, see our Editorial Policies and the Editorial Policy Checklist.

Statistics

For all statistical analyses, confirm that the following items are present in the figure legend, table legend, main text, or Methods section.

n/a	Confirmed	
☐	☒	The exact sample size (*n*) for each experimental group/condition, given as a discrete number and unit of measurement
☐	☒	A statement on whether measurements were taken from distinct samples or whether the same sample was measured repeatedly
☐	☒	The statistical test(s) used AND whether they are one- or two-sided<br>*Only common tests should be described solely by name; describe more complex techniques in the Methods section.*
☐	☒	A description of all covariates tested
☐	☒	A description of any assumptions or corrections, such as tests of normality and adjustment for multiple comparisons
☐	☒	A full description of the statistical parameters including central tendency (e.g. means) or other basic estimates (e.g. regression coefficient) AND variation (e.g. standard deviation) or associated estimates of uncertainty (e.g. confidence intervals)
☐	☒	For null hypothesis testing, the test statistic (e.g. *F*, *t*, *r*) with confidence intervals, effect sizes, degrees of freedom and *P* value noted<br>*Give P values as exact values whenever suitable.*
☒	☐	For Bayesian analysis, information on the choice of priors and Markov chain Monte Carlo settings
☐	☒	For hierarchical and complex designs, identification of the appropriate level for tests and full reporting of outcomes
☐	☒	Estimates of effect sizes (e.g. Cohen's *d*, Pearson's *r*), indicating how they were calculated

Our web collection on statistics for biologists contains articles on many of the points above.

Software and code

Policy information about availability of computer code

Data collection	No software was used for data collection
Data analysis	All statistical analyses were conducted in R version 4.2.1. The R codes are deposited in a community repository.

For manuscripts utilizing custom algorithms or software that are central to the research but not yet described in published literature, software must be made available to editors and reviewers. We strongly encourage code deposition in a community repository (e.g. GitHub). See the Nature Portfolio guidelines for submitting code & software for further information.

Data

Policy information about availability of data

All manuscripts must include a data availability statement. This statement should provide the following information, where applicable:

- Accession codes, unique identifiers, or web links for publicly available datasets
- A description of any restrictions on data availability
- For clinical datasets or third party data, please ensure that the statement adheres to our policy

Our dataset are deposited in an online repository

Research involving human participants, their data, or biological material

Policy information about studies with human participants or human data. See also policy information about sex, gender (identity/presentation), and sexual orientation and race, ethnicity and racism.

Reporting on sex and gender	We did not include any variables related to sex and/or gender
Reporting on race, ethnicity, or other socially relevant groupings	We did not include any variables related to race nor ethnicity.
Population characteristics	Our analysis uses data from the population censuses of 2000 and 2010. Specific information on each census is available from https://www.ibge.gov.br/en/statistics/social/labor/18391-2010-population-census.html?=&t=o-que-e
Recruitment	NA
Ethics oversight	NA

Note that full information on the approval of the study protocol must also be provided in the manuscript.

Field-specific reporting

Please select the one below that is the best fit for your research. If you are not sure, read the appropriate sections before making your selection.

☐ Life sciences ☒ Behavioural & social sciences ☐ Ecological, evolutionary & environmental sciences

For a reference copy of the document with all sections, see nature.com/documents/nr-reporting-summary-flat.pdf

Behavioural & social sciences study design

All studies must disclose on these points even when the disclosure is negative.

Study description	Our study is an assessment of deforestation and poverty outcomes (fiscal income, income inequality, sanitation, and literacy) of Strict, Sustainable-Use Protected Areas and Indigenous Territories compared to different agricultural landholding sizes and legal mining across ~5,500 census tracts in the Brazilian Legal Amazon.
Research sample	The research sample constitutes households surveyed in the 2000 and 2010 population censuses in the Brazilian Legal Amazon.
Sampling strategy	Our study uses census data. Thus, we did not perform surveys ourselves. Specific information about each census is available from https://www.ibge.gov.br/en/statistics/social/labor/18391-2010-population-census.html?=&t=o-que-e
Data collection	Our study uses census data. Thus, we did not perform surveys ourselves. Specific information about each census is available from https://www.ibge.gov.br/en/statistics/social/labor/18391-2010-population-census.html?=&t=o-que-e
Timing	We used data from the 2000 and 2010 population censuses, and the 2006 agricultural census.
Data exclusions	We excluded census tracts with more than 1%, but less than 10%, overlap with a protection arrangement to ensure a clear distinction in the amount of protection between control and treatment census tracts. To further ensure a clear contrast between our different treatments we excluded protected census tracts that contained more than one type of protection.
Non-participation	NA
Randomization	NA

Reporting for specific materials, systems and methods

We require information from authors about some types of materials, experimental systems and methods used in many studies. Here, indicate whether each material, system or method listed is relevant to your study. If you are not sure if a list item applies to your research, read the appropriate section before selecting a response.

Materials & experimental systems

n/a	Involved in the study
☒	☐ Antibodies
☒	☐ Eukaryotic cell lines
☒	☐ Palaeontology and archaeology
☒	☐ Animals and other organisms
☒	☐ Clinical data
☒	☐ Dual use research of concern
☒	☐ Plants

Methods

n/a	Involved in the study
☒	☐ ChIP-seq
☒	☐ Flow cytometry
☒	☐ MRI-based neuroimaging

Plants

Seed stocks	NA
Novel plant genotypes	NA
Authentication	NA
